# Long non-coding RNA NORAD/miR-224-3p/MTDH axis contributes to CDDP resistance of esophageal squamous cell carcinoma by promoting nuclear accumulation of β-catenin

**DOI:** 10.1186/s12943-021-01455-y

**Published:** 2021-12-10

**Authors:** Yunlong Jia, Cong Tian, Hongyan Wang, Fan Yu, Wei Lv, Yuqing Duan, Zishuo Cheng, Xuexiao Wang, Yu Wang, Tianxu Liu, Jiali Wang, Lihua Liu

**Affiliations:** 1 Department of Tumor Immunotherapy, Hebei Medical University Fourth Affiliated Hospital and Hebei Provincial Tumor Hospital, Shijiazhuang, 050035 China; 2 Department of Thoracic Surgery, Hebei Medical University Fourth Affiliated Hospital and Hebei Provincial Tumor Hospital, Shijiazhuang, 050011 China; 3 Cancer Research Institute of Hebei Province, Shijiazhuang, 050011 China; 4 China International Cooperation Laboratory of Stem Cell Research, Hebei Medical University, Shijiazhuang, 050011 China

**Keywords:** ESCC, CDDP resistance, NORAD, miR-224-3p, MTDH

## Abstract

**Background:**

Cis-diamminedichloro-platinum (CDDP)-based chemotherapy regimens are the most predominant treatment strategies for patients with esophageal squamous cell carcinoma (ESCC). Dysregulated long non-coding RNAs (lncRNAs) contribute to CDDP resistance, which results in treatment failure in ESCC patients. However, the majority of lncRNAs involved in CDDP resistance in ESCC remain to be elucidated.

**Methods:**

The public Gene Expression Omnibus (GEO) dataset GSE45670 was analysed to reveal potential lncRNAs involved in CDDP resistance of ESCC. Candidate upregulated lncRNAs were detected in ESCC specimens by qRT-PCR to identify crucial lncRNAs. Non-coding RNA activated by DNA damage (NORAD) was selected for further study. Kaplan-Meier analysis and a COX proportional regression model were performed to analyse the potential of NORAD for predicting prognosis of ESCC patients. The role of NORAD in CDDP resistance were determined by conducting gain and loss-of-function experiments in vitro. Fluorescence in situ hybridization (FISH) was performed to determine the subcellular location of NORAD in ESCC cells. A public GEO dataset and bioinformatic algorithms were used to predict the microRNAs (miRNAs) that might be latently sponged by NORAD. qRT-PCR was conducted to verify the expression of candidate miRNAs. Luciferase reporter and Argonaute-2 (Ago2)-RNA immunoprecipitation (RIP) assays were conducted to evaluate the interaction between NORAD and candidate miRNAs. A miRNA rescue experiment was performed to authenticate the NORAD regulatory axis and its effects on CDDP resistance in ESCC cells. Western blotting was conducted to confirm the precise downstream signalling pathway of NORAD. A xenograft mouse model was established to reveal the effect of NORAD on CDDP resistance in vivo.

**Results:**

The expression of NORAD was higher in CDDP-resistant ESCC tissues and cells than in CDDP-sensitive tissues and cells. NORAD expression was negatively correlated with the postoperative prognosis of ESCC patients who underwent CDDP-based chemotherapy. NORAD knockdown partially arrested CDDP resistance of ESCC cells. FISH showed that NORAD was located in the cytoplasm in ESCC cells. Furthermore, overlapping results from bioinformatic algorithms analyses and qRT-PCR showed that NORAD could sponge miR-224-3p in ESCC cells. Ago2-RIP demonstrated that NORAD and miR-224-3p occupied the same Ago2 to form an RNA-induced silencing complex (RISC) and subsequently regulated the expression of metadherin (MTDH) in ESCC cells. The NORAD/miR-224-3p/MTDH axis promoted CDDP resistance and progression in ESCC cells by promoting nuclear accumulation of β-catenin in vitro and in vivo.

**Conclusions:**

NORAD upregulates MTDH to promote CDDP resistance and progression in ESCC by sponging miR-224-3p. Our results highlight the potential of NORAD as a therapeutic target in ESCC patients receiving CDDP-based chemotherapy.

**Supplementary Information:**

The online version contains supplementary material available at 10.1186/s12943-021-01455-y.

## Background

Esophageal cancer is one of the most common malignant tumours worldwide, constituting the sixth leading cause of cancer-related death [1]. Esophageal squamous cell carcinoma (ESCC) is the dominant subtype of esophageal cancer in China, accounting for more than 90% of cases [2]. Chemotherapy is of utmost importance for ESCC patients, as it occupies an irreplaceable position in both postoperative adjuvant therapy and first-line treatment [3]. At present, cis-diamminedichloro-platinum (CDDP) is the foremost agent for treating ESCC; CDDP-based chemotherapy regimens are still the most frequent treatment strategies for patients with unresectable or relapsed tumours [3]. Nevertheless, drug resistance limits the actual effects of CDDP and consequently worsens the prognosis of ESCC patients [4]. Therefore, there is a need to elucidate the molecular mechanisms underlying CDDP resistance to further improve the prognosis of ESCC patients.

CDDP resistance is a malignant biological property regulated by complicated mechanisms, especially activation of signalling pathways [5–8]. Aberrant activation of signalling pathways, including the Wnt/β-catenin, PI3K/AKT, MAPK and JNK1 pathways, can be induced by numerous carcinogenic factors, among which dysregulation of long non-coding RNAs (lncRNAs) was recently shown to be a pivotal regulator [9]. LncRNAs are a class of transcripts comprising more than 200 nucleotides that lack high cross-species conservation [10]. Although lncRNAs were initially regarded as “dark matter” or “noise” generated during transcription as they have no or limited protein-coding capacity, emerging studies have proven that they are involved in the regulation of numerous cellular biological processes [11]. Multiple lncRNAs have been identified to be involved in the initiation and progression of cancers by participating in transcriptional and posttranscriptional gene expression [10]. Acting as competing endogenous RNAs (ceRNAs) to sponge microRNAs (miRNAs) is one of the most well-studied biological roles of lncRNAs in cancers [12]. LncRNAs competitively sponge miRNAs to neutralize miRNA binding to the 3′-untranslated region (UTR) and thereby posttranscriptionally regulate the expression of corresponding target genes, among which there are numerous key regulators of signalling pathways involved in chemotherapy resistance [9, 11]. For instance, the lncRNA plasmacytoma variant translocation 1 (PVT1) activates the Wnt/β-catenin pathway to promote gemcitabine resistance of pancreatic cancer [13]. Additionally, the lncRNA actin filament-associated protein 1 antisense RNA 1 (AFAP1-AS1) activates AKT signalling by upregulating RRM2 and subsequently promotes CDDP resistance in non-small cell lung cancer (NSCLC) [14]. In addition, the lncRNA carbonyl reductase 3 antisense RNA 1 (CBR3-AS1) activates the JNK1/MEK4-mediated MAPK signalling pathway to promote adriamycin resistance [15]. However, the clinical significance and functional mechanisms of the majority of lncRNAs involved in CDDP resistance in ESCC remain to be elucidated.

In the present study, we analysed a public microarray dataset to screen lncRNAs possibly correlated with CDDP resistance of ESCC. Non-coding RNA activated by DNA damage (NORAD, also known as linc00657) was verified to be a key lncRNA contributing to CDDP resistance in ESCC. NORAD exhibited the potential to be a biomarker for predicting CDDP resistance in ESCC patients. Furthermore, we verified that NORAD acted as a ceRNA to sponge miR-224-3p and thus upregulated metadherin (MTDH, also known as astrocyte elevated gene-1) which promoted nuclear accumulation of β-catenin, a key molecule in CDDP resistance promotion. Our findings unveiled a novel role for NORAD as a therapeutic target to overcome CDDP resistance in ESCC patients.

## Materials and methods

### Patients and specimens

In total, 93 pairs of ESCC and matched paracarcinoma tissue specimens were obtained from the patients who underwent radical resection surgery at the Fourth Hospital of Hebei Medical University between March 2015 and March 2016. None of these ESCC patients received any treatment including radiotherapy, chemotherapy, and immunotherapy. All patients received treatment with standard CDDP-based therapeutic regimens as postoperative adjuvant chemotherapy. Clinical staging was performed according to the Union for International Cancer Control (UICC) Classification of 2010 (seventh edition). CDDP resistance was defined as tumour relapse during CDDP-based chemotherapy after R0 excision, and CDDP sensitivity was defined as no tumour recurrence during CDDP-based therapy; both definitions followed published standard CDDP-response definitions [7, 16]. Patients’ clinical information were collected and stored in a database, which was updated every 2 months by telephone follow-up. Complete follow-up data were updated until death or 5 years after surgery.

### Bioinformatic analysis

A microarray data profile (GSE45670) was downloaded from the Gene Expression Omnibus (GEO) database (http://www.ncbi.nlm.nih.gov/geo/) to screen lncRNAs involved in CDDP resistance in ESCC. The microarray data were analysed by using the limma package in R. RNA-sequencing (RNA-seq) data for an ESCC cohort were downloaded from The Cancer Genome Atlas (TCGA) (http://cancergenome.nih.gov/) to screen the expression of NORAD. The RNA-seq data were analysed by using DESeq2 package in R.

### RNA isolation and quantitative RT-PCR (qRT-PCR) assay

In brief, total RNA was extracted from ESCC cells or tissues by using TRIzol reagent (Thermo Fisher Scientific, Waltham, MA, USA). cDNA was synthesized according to the manufacturer’s instructions by using the RevertAid™ First Strand cDNA Synthesis Kit (Thermo Fisher Scientific). qRT-PCR was conducted with SYBR Green PCR kit (Takara Bio, Otsu, Japan) on a StepOne Real-Time PCR system (Thermo Fisher Scientific). Relative gene expression levels were determined using the 2^-△△CT^ method [17]. Human GAPDH and U6 small nuclear RNA (snRNA) were used to be the internal control for calculating the relative expression of lncRNAs and miRNAs, respectively. The primer sequences were listed in Additional file 1: Table S1 and S2.

### Fluorescence in situ hybridization (FISH)

A double FISH assay was performed as previously described [18]. FISH probes were designed and synthesized by Servicebio (Wuhan, China). Dig-labelled probes specific for NOARD and biotinylated locked nucleic acid miR-224-3p probes were used during hybridization. The probe sequences were as follows: NORAD, 5′-GGGTTTCGTCGAGGCTTGGGTCGG-3′; and miR-224-3p, 5′- TGTAGTCACTAGGGCACCATTTT-3′. Samples were counterstained with 6-diamidino-2-phenylindole (DAPI) and observed by confocal microscopy. The FISH score for NORAD in ESCC tissues was determined by evaluating the proportion of positive cells detected within 5 random fields of view on every slide (400-fold magnification), as Huang et al. described [7]. In brief, scores were assigned as follows: < 10% = 0, 10–25% = 1, 26–50% = 2, 51–75% = 3, and > 75% = 4.

### Cell culture

The human ESCC cell lines KYSE30 and TE1 were obtained from Procell Life Science & Technology Co., Ltd. (Wuhan, Hubei, China). We referred to the protocols from the study of Toshimitsu et al. to establish CDDP-resistant strains of KYSE30 and TE1, referred to KYSE30/CDDP-R and TE1/CDDP-R, respectively [19]. The detailed methods are described in Additional file 3: Methods of Establishing CDDP-resistant cell line. All cells were maintained in RPMI-1640 medium (Servicebio) containing 5% fetal bovine serum (FBS) (Servicebio), 100 U/ml penicillin and 100 μg/ml phytomycin in an incubator with 5% CO_2_ at 37 °C.

### Vector construction and lentiviral infection

DNA fragments encoding NORAD were amplified from the genomic DNA of KYSE30/CDDP-R cells. Then, NORAD was subcloned into the lentiviral vector LV-EF1a-GFP-Puro (GenePharma, Shanghai, China). After transfection for 48 h, cells were treated with puromycin (Cayman Chemical, Ann Arbor, MI, USA) at a concentration of 2 μg/ml to screen out the cells successfully transfected with the lentiviral vectors. Then, the single clones were isolated by limiting dilution in the presence of puromycin. The expression of NORAD was detected in different clones by qRT-PCR. The clone exhibiting lowest expression of NORAD was used in subsequent experiments. In addition, the lentiviral vector LV-3 pGLVU6/Puro (GenePharma) was used for packaging short hairpin RNAs (shRNAs) to knock down NORAD expression. shRNAs for knocking down NORAD were designed and synthesized by GenePharma and the sequences are listed in Additional file 1: Table S3. After transfection for 48 h, the cells were treated with puromycin followed by limiting dilution to screen out single clones. The clone exhibiting highest expression of NORAD was used in subsequent experiments.

### Colony formation assay

ESCC cells were cultured with CDDP at the indicated concentrations for 3 h. Then, the ESCC cells were harvested and seeded in 6-well plates (500 cells per well). and cultured in an incubator with 5% CO_2_ at 37 °C. After 14 days, the cells were fixed with methanol for 15 min and stained with crystal violet for 15 min. Colonies (≥ 50 cells) were counted by using ImageJ. Experiments were performed in triplicate.

### Luciferase reporter assay

Sequences for wild-type (WT) or mutant (Mut) NORAD or the 3′-UTR of MTDH containing the predicted binding sites for miR-224-3p were subcloned into a psiCHECK-2 vector (Hanbio Biotechnology, Shanghai, China). The luciferase reporter plasmids were co-transfected into ESCC cells with a miR-224-3p mimic or negative control (NC). After 48 h, the cells were collected and counted. A total of 5 × 10^5^ cells were used to measure luciferase activity. Relative luciferase activity was measured by using an HBLumi Dual-luciferase reporter assay kit (Hanbio Biotechnology) according to the manufacturer’s instructions. Renilla luciferase activity was normalized to firefly luciferase activity. To evaluate the effect of the miR-224-3p mimic on firefly luciferase activity, the miRNA mimic NC group was used as control. All assays were carried out in triplicate. The mutant sequences of NORAD and the MTDH-3′-UTR were created by using the FAST Site-Directed Mutagenesis Kit (Tiangen, Beijing, China).

### Western blotting

For western blotting analysis, proteins were extracted by using RIPA lysis buffer (Beyotime, Shanghai, China). Proteins were added to 4–20% gels, subjected to 160 V for separation, and then transferred to PVDF membranes (Millipore, Billerica, MA, USA). The membranes were blocked with 5% BSA for 1 h and incubated at 4 °C overnight with the following primary antibodies: anti-MTDH (ab227981, Abcam, Cambridge, UK), anti-NF-κB p65 (#8242, Cell Signaling Technology, Danvers, MA, USA), anti-phospho-NF-κB p65 (Ser536) (#3033, Cell Signaling Technology), anti-p38 MAPK (#8690, Cell Signaling Technology), anti-phospho-p38 MAPK (#4511, Cell Signaling Technology), anti-Akt (#9272, Cell Signaling Technology), anti-phospho-Akt (Thr308) (#13038, Cell Signaling Technology), anti-β-catenin (GB11015, Servicebio), anti-phospho-β-catenin (Ser675) (#8480, Cell Signaling Technology), anti-γH2AX (GB111841, Servicebio), anti-caspase-3 (GB11767C, Servicebio), anti-cleaved caspase-3 (ab32042, Abcam), anti-E-cadherin (GB11868, Servicebio), anti-N-cadherin (GB11135, Servicebio), anti-MMP9 (GB11132, Servicebio), anti-Histone H3 (ab176842, Abcam) and anti-GAPDH (GB11002, Servicebio). The next day, the membranes were washed 3 times for 15 min in TBST and incubated with secondary antibodies for 2 h at room temperature. Then, the protein bands were developed with an ECL chemiluminescent substrate kit (Biosharp Life Sciences, Hefei, China). Band intensities were quantified using ImageQuant LAS 500 (GE, Boston, MA, USA). To evaluate the expression of β-catenin located in cytoplasm and nucleus, GAPDH and Histone H3 were used as the respective internal controls.

### Cell viability assay

ESCC cells (2 × 10^4^/mL) were incubated with CDDP (PHR1624, Energy Chemical, Shanghai, China) at different concentrations (1–10 μg/mL, diluted with RPMI-1640 medium) for 48 h. Then, cell viability was measured by using a cell counting kit-8 (CCK-8) assay according to the manufacturer’s instructions. Briefly, approximately 2 × 10^3^ cells were plated in 96-well plates. Following cell adhesion, 10 μl of CCK-8 reagent (Servicebio) was added to each well, and the plates were incubated for 2 h in a humidified incubator containing 5% CO_2_ at 37 °C. The absorbance of each well was detected at a wavelength of 450 nm.

### Flow cytometry (FCM)

The FCM assay was performed as described in our previous study [20]. The Annexin V-FITC/PI kit (Servicebio) and Cell Cycle and Apoptosis Analysis Kit (Servicebio) were used to assay the apoptosis and cell cycle, respectively.

### miRNA inhibitor and mimic transfection

A miR-224-3p mimic, miR-224-3p inhibitor, miRNA mimic NC and miRNA inhibitor NC were purchased from Thermo Fisher Scientific. The sequences used are listed in Additional file 1: Table S4. Transfection was carried out using Lipofectamine 3000 (Thermo Fisher Scientific) according to the manufacturer’s instructions.

### Immunohistochemistry (IHC)

IHC staining was performed and analysed according to previously described protocols as previously described [21]. Primary antibodies were listed as follow: anti-MTDH (ab227981, Abcam), anti-β-catenin (GB11015, Servicebio), anti-γH2AX (GB111841, Servicebio) and anti-cleaved caspase-3 (GB11532, Servicebio). Staining was evaluated by using IHC Profiler in ImageJ. Staining scores of 0 and 1+ were regarded as negative expression, and scores of 2+ and 3+ were regarded as positive expression.

### Wound healing assay

To detect the migratory abilities, ESCC cells were seeded and cultured in 6-well plates with serum-free RMPI-1640 medium, and the cell monolayer were scraped linearly to introduce an artificial wound that was imaged at 0 h and 48 h.

### Transwell invasion assay

To detect the invasive ability of ESCC cells, the inserts of Transwell chambers (Corning, New York, USA) were coated with 50 μl of 1 mg/ml Matrigel matrix (Solarbio), according to the manufacturer’s instructions. ESCC cells (5 × 10^4^) in 200 μl of FBS-free medium were plated in the upper chamber, while 600 μl of medium containing 10% FBS was added to the lower chamber. After an incubation for 24 h at 37 °C with 5% CO_2_, the cells that did not penetrate the membrane were removed with a cotton swab, and the invading cells were fixed and stained with 0.1% crystal violet.

### RNA immunoprecipitation (RIP) assay

RIP was performed using a Magna RIP RNA-Binding Protein Immunoprecipitation kit (Millipore, Billerica, MA, USA) according to the manufacturer’s instructions with slight modifications as previously described [22]. An anti-Argonaute-2 (Ago2) (ab186733, Abcam) antibody was used in the RIP assay.

### Isolation of nuclear and cytoplasmic fractions

The cytosolic and nuclear proteins of ESCC cells were collected using the Nuclear and Cytoplasmic Protein Extraction Kit (Beyotime). The cytosolic and nuclear proteins of ESCC cells were extracted by using PARIS™ Kit (Thermo Fisher Scientific). The detailed experimental procedures were performed according to the manufacturer’s instructions.

### Nude mouse xenograft model

The animal experiment was approved by the Animal Research Committee of the Fourth Hospital of Hebei Medical University. Female BALB/c nude mice (4 weeks old, 5 per group) were purchased from HFK Bioscience (Beijing, China) and maintained under pathogen-free conditions. A total of 5 × 10^6^ ESCC cells in 100 μl of PBS were subcutaneously injected into the flank of mice. One week after injection, we intraperitoneally injected mice with CDDP (3 mg/kg) in PBS or PBS alone once every 2 days. Tumour size (length: L, and width: W) was measured every 4 days. Tumour volume was calculated by using the formula: 1/2 × L × W^2^. The xenograft tumours were harvested after 5 weeks and then weighed.

### Statistical analysis

Statistical analysis was performed by using IBM SPSS Statistics 26.0. The figures were drawn in GraphPad Prism 9.2 and Origin 2021. All measurement data are presented as the mean ± SD. Correlations between NORAD expression and clinical characteristics were evaluated by using the Kendall rank test. Survival analysis was carried out using the log-rank test in association with Kaplan-Meier analysis and the Cox proportional hazards model. Student’s t-test was used to analyse data with equal variance, and the Mann-Whitney U test was used for those without equal variance. Spearman rank correlation was used to analyse the associations between NORAD and miR-224-3p or among MTDH, NORAD and miR-224-3p in ESCC tissue specimens. Pearson correlation was used to analyse the correlation between NORAD and miR-224-3p in cell lines. A *P* value < 0.05 was considered statistically significant, and all *P* values were two-tailed.

## Results

### High expression of NORAD is associated with CDDP resistance in ESCC

**Fig. 1:**
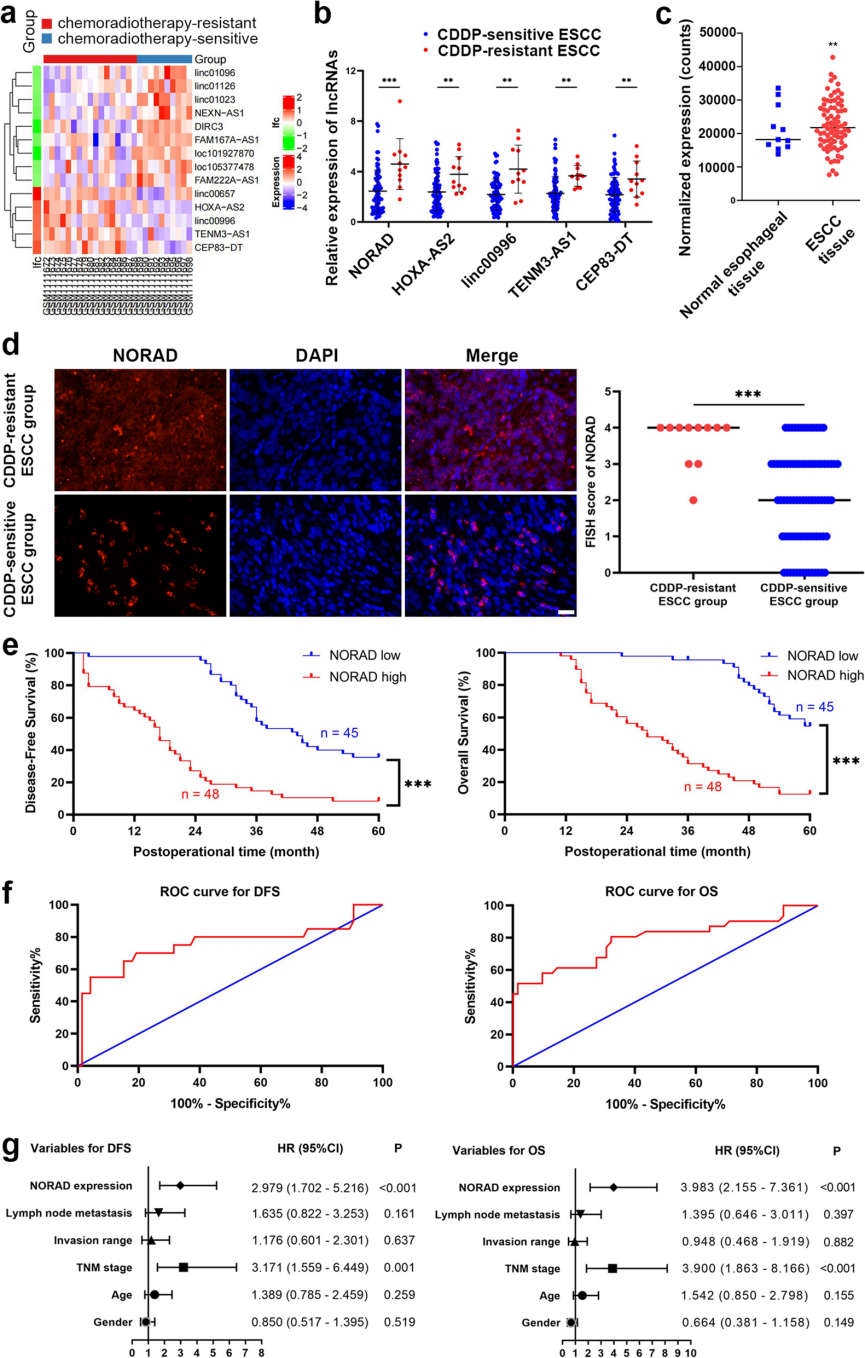
NORAD expression is increased in CDDP-resistant ESCC tissues and correlated with poor prognosis of patients. **a** The differential lncRNAs between chemoradiotherapy-sensitive and chemoradiotherapy-resistant ESCC in public GSE45670 dataset. **b** Validated expression of 5 upregulated lncRNAs in the tissues from 93 ESCC patients by using qRT-PCR. Error bars indicate SD. *** *P* < 0.001. **c** The expression of NORAD in ESCC tissues and normal esophageal tissues in TCGA database. ** *P* < 0.01. **d** Representative images of RNA FISH of NORAD in ESCC tissues (× 400), which show that NORAD is predominantly located in the cytoplasm. Nuclei are stained with DAPI. FISH score of NORAD in CDDP-resistant ESCC group was higher, compared to CDDP-sensitive ESCC group. **e** Kaplan-Meier analysis curves of DFS and OS for ESCC patients with high (*n* = 48) or low (*n* = 45) NORAD expression. The cut-off value was calculated by using X-tile. **f** ROC curves of NORAD for predicting DFS and OS in ESCC patients. **g** Multivariate analyses of hazard ratios for DFS and OS in ESCC patients

To characterize the key lncRNAs involved in CDDP resistance in ESCC, we first analysed the GEO dataset GSE45670 (https://www.ncbi.nlm.nih.gov/geo/query/acc.cgi?acc=gse45670), which contained the differential lncRNA and mRNA profiles between tissues from CDDP-based chemoradiotherapy-sensitive and CDDP-based chemoradiotherapy-resistant ESCC patients. The analysis identified 5 upregulated lncRNAs in chemoradiotherapy-resistant ESCC tissues by setting the cut-off value as a fold-change of 2, chemoradiotherapy-sensitive tissues were used as a control (Fig. 1a). Then we detected these 5 candidate lncRNAs in 93 ESCC tissue samples including 82 cases sensitive to CDDP and 11 resistant to CDDP. Consistent with the results from the analysis of GSE45670, the expression of these 5 lncRNAs was upregulated in the CDDP-resistant ESCC tissues, compared to the CDDP-sensitive tissues (Fig. 1b). Among these 5 candidate lncRNAs, NORAD exhibited the most significant difference between CDDP-resistant and CDDP-sensitive ESCC tissues (*P* <  0.001, Fig. 1b). Furthermore, we analysed the RNA-seq data of an ESCC cohort in the TCGA database and found that the expression of NORAD was higher in ESCC tissues than in normal esophageal esophageal tissues, indicating that NORAD might promote ESCC progression (*P* = 0.005; Fig. 1c).

To further elucidate the correlation between NORAD and CDDP resistance, we detected the expression of NORAD in ESCC tissues by FISH. The FISH score data demonstrated that the expression of NORAD was significantly higher in CDDP-resistant ESCC tissues than in CDDP-sensitive tissues (*P* = 0.002, Fig. 1d). Then, to reveal the potential clinical significance of NORAD, we classified ESCC patients into two groups according to their NORAD expression level determined by qRT-PCR. The NORAD cut-off value used for analyzing disease-free survival (DFS) and overall survival (OS) was 3.34, which was calculated with expression level of NORAD in ESCC tissues, DFS/OS time and recurrence/survival status by using X-tile [23]. Afterwards, we analysed the correlations between NORAD expression and clinical characteristics of ESCC patients. We found that NORAD expression was significantly correlated with CDDP sensitivity, TNM stage, invasion range and lymph node metastasis but not correlated with sex or age (Table 1). Subsequently, we performed Kaplan-Meier analysis to evaluate the clinical significance of NORAD in CDDP resistance in ESCC. Compared to ESCC patients exhibiting low expression of NORAD, patients exhibiting high expression of NORAD had shorter DFS and OS (both *P* <  0.001, Fig. 1e). To further verify the predictive potential of NORAD in CDDP resistance in ESCC, we drew receiver operating characteristic (ROC) curves for the expression levels of NORAD and then calculated the area under the ROC curves (AUC). The AUCs of NORAD for the DFS and OS of ESCC patients were 75.82 and 78.77%, respectively (*P* = 0.004, *P* < 0.001; Fig. 1f), suggesting that NORAD might be a predictive biomarker of CDDP resistance in ESCC patients. Then, we performed a Cox proportional hazards model and found that NORAD expression and TNM stage were both independent risk factors for DFS and OS in ESCC patients (Fig. 1g). These results demonstrated that NORAD was significantly correlated with CDDP resistance and might be a potential biomarker for predicting the prognosis of ESCC patients.

**Table 1:** Correlations between NORAD expression with clinical characteristics of ESCC patients

		n (%)		*P*
	Total	High expression of NORAD	Low expression of NORAD	
Gender				
Male	53	31 (58.49%)	22 (41.51%)	0.129
Female	40	17 (42.50%)	23 (57.50%)	
Age				
< 60	71	39 (54.93%)	32 (45.07%)	0.255
≥ 60	22	9 (40.91%)	13 (59.09%)	
TNM stage				
I + II	63	24 (38.10%)	39 (61.90%)	< 0.001
III	30	24 (80.00%)	6 (20.00%)	
Invasion range				
T1 + T2	62	27 (43.55%)	35 (56.45%)	0.021
T3 + T4	31	21 (67.74%)	10 (32.26%)	
Lymph node metastasis				
Negative	31	9 (29.03%)	22 (70.97%)	0.002
Positive	62	39 (62.90%)	23 (37.10%)	
CDDP sensitivity				
Sensitive	82	38 (46.34%)	44 (53.66%)	0.005
Resistant	11	10 (90.91%)	1 (9.09%)	

### NORAD facilitates CDDP resistance in ESCC in vitro

**Fig. 2:**
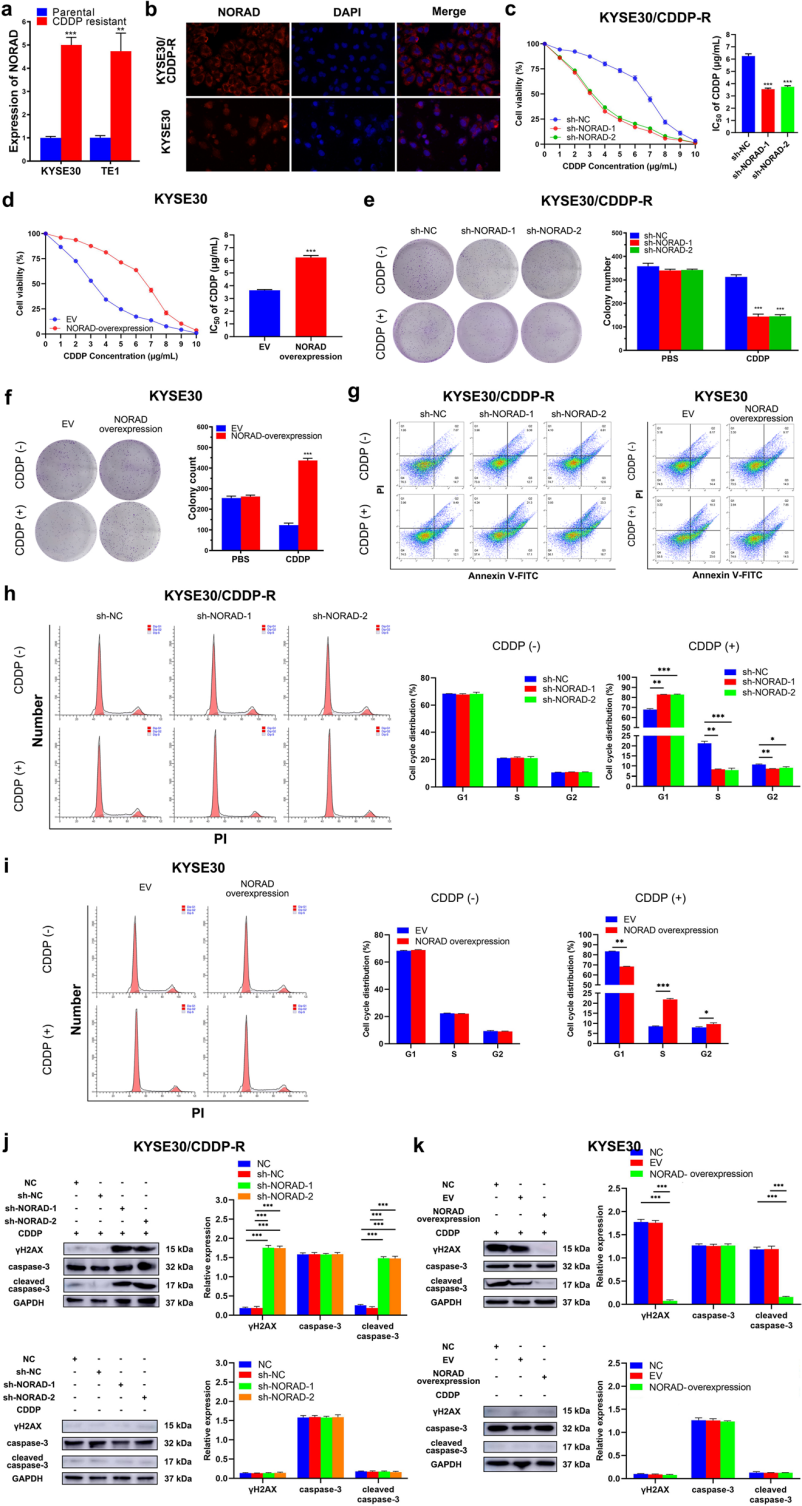
NORAD contributes to CDDP resistance of ESCC. **a** The expression of NORAD in CDDP resistant and matched parental ESCC cells, normalized to GAPDH expression. **b** Representative images of RNA FISH of NORAD in KYSE30/CDDP-R and KYSE30 cells (× 400), which show that NORAD is predominantly located in the cytoplasm. Nuclei are stained with DAPI. **c** NORAD knockdown increases the sensitivity of KYSE30/CDDP-R cells to CDDP, detected by CCK-8 assay. **d** Overexpression of NORAD decreases the sensitivity of KYSE30 cells to CDDP, detected by CCK-8 assay. **e** NORAD knockdown decreases the colony formation ability in KYSE30/CDDP-R cells in the presence of CDDP. **f** Overexpression of NORAD increases the colony formation ability of KYSE30 in the presence of CDDP. **g** NORAD knockdown increases the CDDP-induced apoptosis rate in KYSE30/CDDP-R cells, and overexpression of NORAD decreases this rate in KYSE30 cells, detected by FCM. **h** NORAD knockdown facilitates CDDP to induce cell cycle arrest in KYSE30/CDDP-R cells. i Overexpression of NORAD suppresses CDDP-induced cell cycle arrest in KYSE30 cells. **j** NORAD knockdown increases the CDDP-induced γH2AX and cleaved caspase-3 in KYSE30/CDDP-R cells. **k** Overexpression of NORAD decreases the CDDP-induced γH2AX and cleaved caspase-3 in KYSE30 cells. In all cases, error bars denote SD of triplicates. **P < 0.01, ***P < 0.001

To further study the functional mechanism of CDDP resistance in ESCC, we used CDDP-sensitive, parental ESCC cell lines (KYSE30 and TE1) and their isogenic CDDP-resistant counterparts (KYSE30/CDDP-R and TE1/CDDP-R) as experimental cell lines. The TE1 and KYSE30 cell lines were established by Nishihira et al. and Shimada et al. in 1979 and 1992, respectively [24, 25]. As KYSE30 and TE1 cells are relatively sensitive to CDDP, they are frequently used to establish CDDP-resistant strains for studying mechanisms involved in CDDP resistance in ESCC [26, 27]. The half maximal inhibitory concentration (IC_50_) values of CDDP for KYSE30/CDDP-R and TE1/CDDP-R cells were 5.74 and 5.83 μg/mL, and the IC_50_ values of CDDP for KYSE30 and TE1 were 2.89 and 2.95 μg/mL, respectively (Additional file 2: Fig. S1a-b). Thus, these IC_50_ values were chosen to be the concentrations of CDDP used in subsequent experiments. Next, we detected the expression of NORAD in KYSE30/CDDP-R and TE1/CDDP-R cells. Consistent with the results from the GSE45670 dataset, the expression of NORAD in KYSE30/CDDP-R and TE1/CDDP-R cells was significantly higher than that in the corresponding parental cells (fold-change: 4.70 ± 0.38 and 5.02 ± 0.59, both *P* < 0.001; Fig. 2a). Subsequently, we detected the subcellular location of NORAD by FISH, and found that NORAD was located mainly in the cytoplasm of ESCC cells (Fig. 2b & Additional file 2: Fig. S2). Furthermore, to confirm the effect of NORAD on ESCC cells, we constructed shRNAs to knock down NORAD expression in CDDP-resistant ESCC cells, and plasmids to overexpress NORAD in parental ESCC cells (Additional file 2: Fig. S3a-b). A CCK-8 assay was performed to reveal the effect of NORAD on the proliferative ability of ESCC cells. Altering the expression of NORAD had no significant effect on the proliferative ability of KYSE30/CDDP-R and TE1/CDDP-R cells (Additional file 2: Fig. S3c-d). Then, we treated ESCC cells with CDDP and assayed cell viability to calculate the IC_50_ value. The results showed that knocking down NORAD expression significantly reduced the IC_50_ value of CDDP, indicating that this change rescued CDDP resistance in KYSE30/CDDP-R and TE1/CDDP-R cells (Fig. 2c & Additional file 2: Fig. S4a). Moreover, overexpression of NORAD remarkably decreased the sensitivity of KYSE30 and TE1 cells to CDDP (Fig. 2d & Additional file 2: Fig. S4b). More importantly, NORAD knockdown reduced the colony formation abilities of KYSE30/CDDP-R and TE1/CDDP-R cells only when they were treated with CDDP, not PBS, indicating that NORAD knockdown partly restored the sensitivity of these cells to CDDP (Fig. 2e & Additional file 2: Fig. S4c). Moreover, the colony formation abilities of KYSE30 and TE1 cells were enhanced by NORAD overexpression and this enhancement was more remarkable when the cells were treated with CDDP at the indicated concentration, compared to empty vector (EV) group (Fig. 2f & Additional file 2: Fig. S4d). Then, we assayed the apoptosis rate to further determine the effect of NORAD on CDDP resistance in ESCC cells. Knocking down NORAD significantly increased CDDP-induced apoptosis in KYSE30/CDDP-R and TE1/CDDP-R cells (Fig. 2g & Additional file 2: Fig. S4e). Likewise, NORAD overexpression significantly reduced CDDP-induced apoptosis in parental KYSE30 and TE1 cells (Fig. 2g & Additional file 2: Fig. S4e). To further elucidate the effect of NORAD on the CDDP-induced DNA damage in ESCC cells, we assayed the cell cycle by conducting FCM. As shown in Fig. 2h, CDDP induced a G1 phase arrest in KYSE30/CDDP-R and TE1/CDDP-R cells in which NORAD was knocked down (Fig. 2h & Additional file 2: Fig. S4f). Similarly, CDDP-induced G1 phase arrest was partly neutralized by NORAD overexpression in KYSE30 and TE1 cells (Fig. 2i & Additional file 2: Fig. S4g). Additionally, we detected the DNA damage marker γH2AX and apoptosis marker cleaved caspase-3 by western blotting analysis. Knocking down NORAD resulted in significantly increased expression of γH2AX and cleaved caspase-3 in KYSE30/CDDP-R and TE1/CDDP-R cells when they were treated with CDDP (Fig. 2j & Additional file 2: Fig. S4h). Similarly, the levels of CDDP-induced γH2AX and cleaved caspase-3 in KYSE30 and TE1 cells were reduced by NORAD overexpression (Fig. 2k & Additional file 2: Fig. S4i). Overall, upregulated NORAD could promote CDDP resistance of ESCC cells.

### NORAD regulates the expression of MTDH in ESCC cells by sponging miR-224-3p

**Fig. 3:**
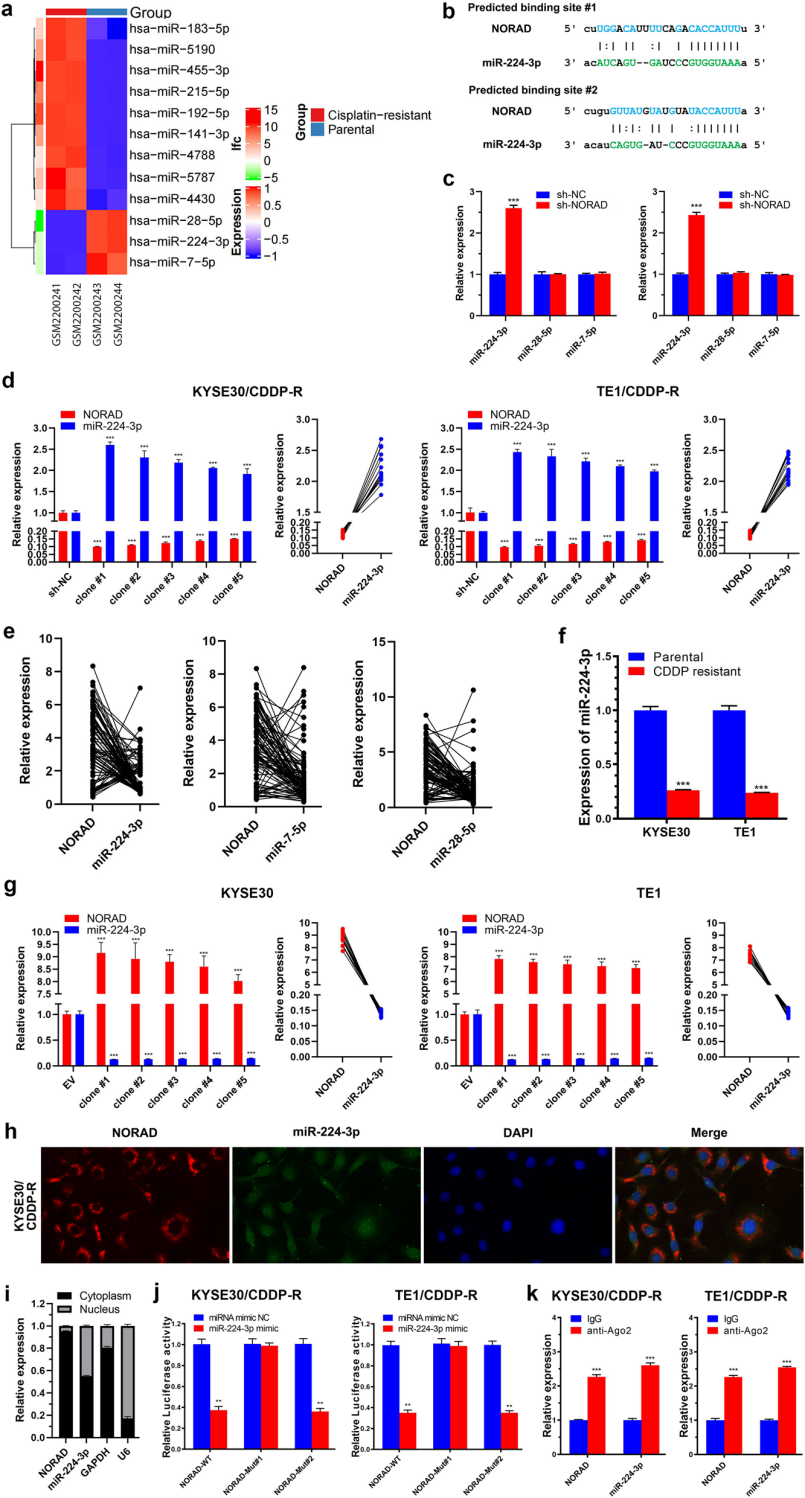
NORAD sponges miR-224-3p in ESCC. **a** The differential miRNAs between CDDP-resistant and CDDP-sensitive ESCC cells in public GSE83362 dataset. **b** Predicted binding sites on NORAD to sponge miR-224-3p. **c** The effect of sh-NORAD on the expressions of miR-224-3p, miR-28-5p and miR-7-5p in ESCC cells, normalized to U6 expression. **d** The expression of NORAD and miR-224-3p in multiple clones of KYSE30/CDDP-R and TE1/CDDP-R cells. NORAD and miR-224-3p was negatively correlated. **e** NORAD is negatively correlated with miR-224-3p in ESCC specimens while not with miR-28-5p and miR-7-5p. **f** The expression of miR-224-3p in CDDP-resistant and matched parental ESCC cells, normalized to U6 expression. **g** The expression of NORAD and miR-224-3p in multiple clones of KYSE30 and TE1 cells. NORAD and miR-224-3p was negatively correlated. **h** Representative images of RNA FISH of NORAD and miR-224-3p in KYSE30/CDDP-R cells (× 1000), which show that NORAD and miR-224-3p are co-located in the cytoplasm. Nuclei are stained with DAPI. **i** The nuclear and cytoplasmic expression of NORAD and miR-224-3p in KYSE30/CDDP-R cells. **j** Luciferase reporter assay showing the luciferase activity of NORAD-WT, NORAD-Mut#1 and NORAD-Mut#2 in KYSE30/CDDP-R and TE1/CDDP-R cells which are co-transfected with miR-224-3p mimic. **k** Ago2-RIP assay shows that NORAD and miR-224-3p occupy the same Ago2 protein. In all cases, error bars denote SD of triplicates. **P < 0.01, ***P < 0.001

One of the most well-studied functions of lncRNAs, especially cytoplasmic lncRNAs, is acting as ceRNAs to sponge miRNAs [12]. To explore miRNAs involved in CDDP resistance in ESCC, we analysed the GSE83362 dataset which contains the differential miRNAs between CDDP-resistant and CDDP-sensitive ESCC cells. After setting the cut-off at a fold change of 2 and *P* < 0.001, we found 3 miRNAs with lower expression in CDDP-resistant ESCC cells than in the corresponding parental cells (Fig. 3a). By using the bioinformatic algorithm starBase (http://starbase.sysu.edu.cn/), we found that only miR-224-3p exhibited the potential to bind to NORAD (Fig. 3b). To validate the starBase results, we knocked down NORAD in KYSE30/CDDP-R and TE1/CDDP-R cells and then detected the expression of the above 3 candidate miRNAs. By performing limited dilution, we screened out single clone, referred to clone #1, which exhibited lowest expression of NORAD and then assayed the expression of miR-224-3p by qRT-PCR. The results showed that only miR-224-3p expression was increased by knocking down NORAD in both KYSE30/CDDP-R and TE1/CDDP-R cells (Fig. 3c). Then, we assayed the expression of miR-224-3p in some other clones to further verify its correlation with NORAD in ESCC. Knocking down NORAD increased the expression of miR-224-3p in multiple clones of KYSE30/CDDP-R and TE1/CDDP-R cells, indicating that NORAD was negatively correlated with miR-224-3p (R = − 0.935, R = − 0.852, *P* < 0.001; Fig. 3d). Additionally, we detected the expression of these miRNAs in our collected ESCC tissue specimens and analysed their correlations with NORAD. The results showed that only miR-224-3p was negatively correlated with NORAD in the ESCC tissues (*r* = − 0.389, *P* < 0.001); miR-28-5p and miR-7-5p were not correlated (*r* = − 0.026, *P* = 0.808; *r* = 0.034, *P* = 0.747) (Fig. 3e). Moreover, we found that KYSE30/CDDP-R and TE1/CDDP-R cells exhibited lower expression of miR-224-3p than the corresponding parental cells (Fig. 3f). Furthermore, we overexpressed NORAD in KYSE30 and TE1 cells and screened out the single clones to detect the expression of miR-224-3p. The expression of miR-224-3p in KYSE30 and TE1 cells was decreased by overexpressing NORAD, and NORAD was negatively correlated with miR-224-3p (R = − 0.521, *P* = 0.47; R = − 0.768, *P* = 0.001; Fig. 3g). These results provided evidence that miR-224-3p might be a miRNA sponged by NORAD in ESCC.

Then, we detected the subcellular locations of NORAD and miR-224-3p in CDDP-resistant ESCC cells by performing FISH and nuclear and cytoplasmic fraction isolating experiment. The results showed that NORAD and miR-224-3p were co-localized in the cytoplasm of KYSE30/CDDP-R and TE1/CDDP-R cells (Fig. 3h-i & Additional file 2: Fig. S5a-b). To investigate whether NORAD could sponge miR-224-3p in ESCC cells, we constructed luciferase reporters harbouring NORAD with mutations in predicted sites #1 and #2 (i.e., NORAD-Mut#1 and NORAD-Mut#2) for binding to miR-224-3p (Additional file 2: Fig. S6a-b). Then, the luciferase reporter carrying NORAD-WT or NORAD-Mut was transfected into CDDP-resistant ESCC cells, followed by co-transfection of miR-224-3p mimics. The results showed that the miR-224-3p mimics reduced the luciferase activity of NORAD in KYSE30/CDDP-R and TE1/CDDP-R cells transfected with NORAD-WT or NORAD-Mut#2 (Fig. 3j). In contrast, the luciferase activity of NORAD in KYSE30/CDDP-R and TE1/CDDP-R cells transfected with NORAD-Mut#1 was not reduced by the miR-224-3p mimics (Fig. 3j). The above results demonstrated that NORAD could directly bind to miR-224-3p through its miRNA recognition site #1. Then, we performed Ago2-RIP and qRT-PCR assays to verify whether NORAD and miR-224-3p occupied the same RNA-induced silencing complex (RISC). The results showed that the expressions of NORAD and miR-224-3p were significantly higher in Ago2 pellets, than in IgG pellets, indicating that NORAD might act as a ceRNA to sponge miR-224-3p and form a RISC in ESCC cells (Fig. 3k).

**Fig. 4:**
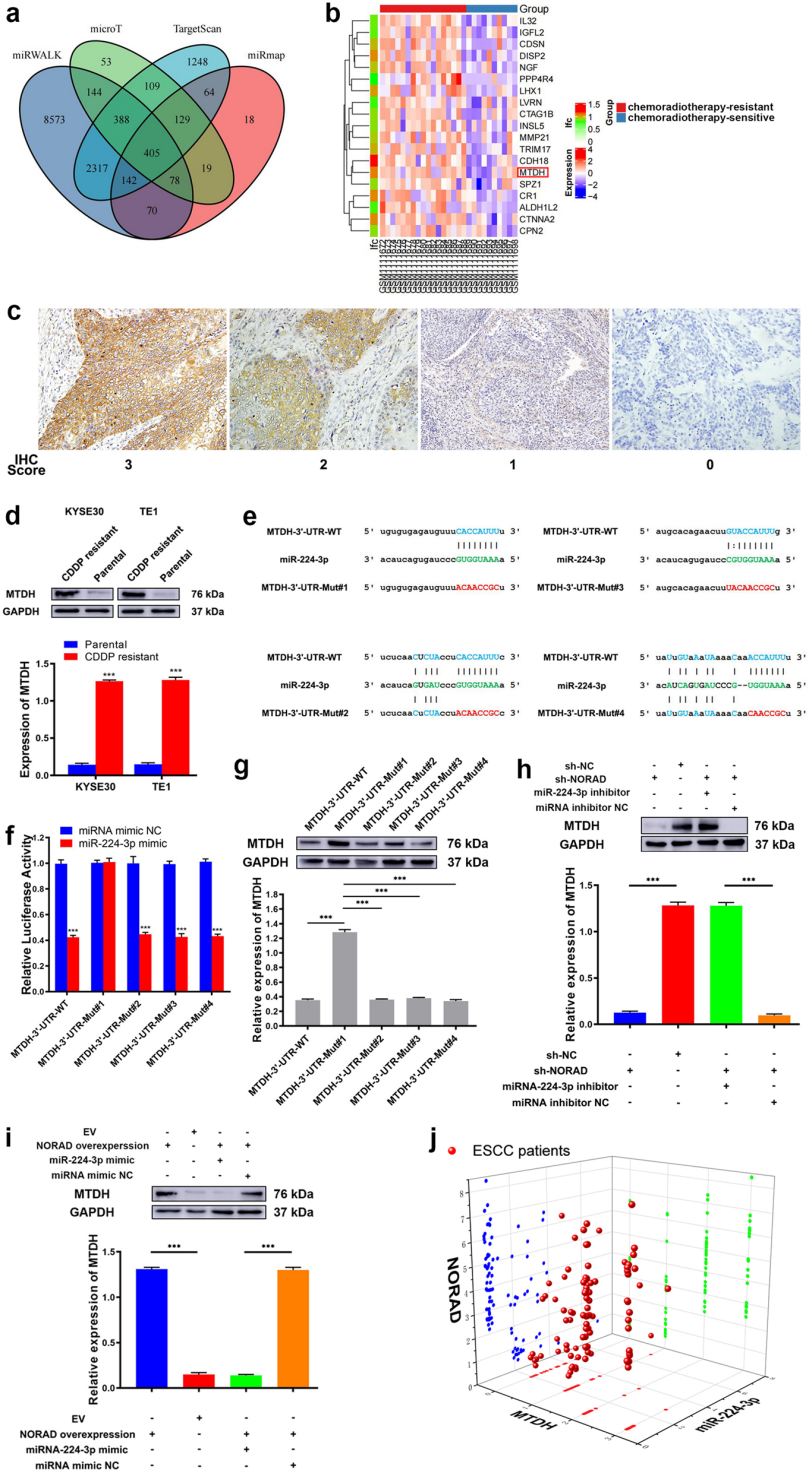
MTDH is a direct target of miR-224-3p. **a** Venn diagram showing the putative target genes of miR-224-3p computationally predicted by 4 algorithms (miRmap, TargetScan, miRWalk and DIANA microT). **b** The TOP20 upregulated genes in chemoradiotherapy-resistant ESCC, compared to chemoradiotherapy-sensitive ESCC, in public GSE45670 dataset. **c** Representative images of positive and negative IHC staining of MTDH in ESCC tissues (× 200). MTDH predominantly locates in cytoplasm. **d** The expression of MTDH is higher in CDDP-resistant ESCC cells than in matched parental cells. **e** Predicted binding sites on 3′-UTR of MTDH to sponge miR-224-3p and matched established mutant sequences. **f** Luciferase reporter assay shows the luciferase activity of MTDH-3′-UTR-WT, MTDH-3′-UTR-Mut#1, MTDH-3′-UTR-Mut#2, MTDH-3′-UTR-Mut#3 and MTDH-3′-UTR-Mut#4 in KYSE30/CDDP-R cells co-transfected with miR-224-3p mimic. **g** The effects of miR-224-3p mimic on the expression of MTDH in KYSE30/CDDP-R cells transfected with MTDH-3′-UTR-WT, MTDH-3′-UTR-Mut#1, MTDH-3′-UTR-Mut#2, MTDH-3′-UTR-Mut#3 or MTDH-3′-UTR-Mut#4. **h** NORAD knockdown downregulates MTDH expression in KYSE30/CDDP-R cells and miR-224-3p mimic rescues this downregulation. **i** Overexpression of NORAD upregulates MTDH expression in KYSE30 cells and miR-224-3p inhibitor rescues this downregulation. **j** Three-dimensional scatter plot of NORAD, miR-224-3p and NORAD expressions in 11 CDDP-resistant and 82 CDDP-sensitive ESCC tissues from patients. **g-i** Error bars denote SD of triplicates. ***P < 0.001

Then, we used 4 bioinformatics algorithms (miRmap, TargetScan, miRWalk and DIANA microT) to predict the potential regulatory target of miR-224-3p. The results generated with the bioinformatic algorithms were listed in Additional file 1: Table S5. After overlapping the results of the above algorithms, we found 405 mRNAs that showed an immense possibility of being regulated by miR-224-3p (Fig. 4a). Subsequently, we analysed the GSE45670 dataset again to find differential mRNAs between chemoradiotherapy-resistant and chemoradiotherapy-sensitive ESCC tissues. The top 20 upregulated genes are shown in Fig. 4b. Among the top 20 upregulated genes in the chemoradiotherapy-resistant ESCC tissues, only MTDH was found in the overlapping results of the bioinformatic algorithms. Thus, to investigate whether MTDH was a candidate factor involved in NORAD resistance in ESCC, we next detected its expression in our collected ESCC tissues by IHC. As Fig. 4c shows, MTDH staining was mainly located in the cytoplasm of ESCC cells. Among 11 CDDP-resistant ESCC tissue samples, 10 (90.91%) exhibited positive expression of MTDH. In contrast, only 46 of 82 samples (56.10%) of CDDP-sensitive ESCC tissues exhibited positive expression of MTDH. The positive expression rate of MTDH in CDDP-resistant ESCC tissues was significantly higher than that in CDDP-sensitive tissues (*P* = 0.024). Afterwards, we detected the expression of MTDH in CDDP-resistant and CDDP-sensitive ESCC cells. Consistent with the results for ESCC tissues, MTDH expression was significantly higher in KYSE30/CDDP-R and TE1/CDDP-R cells than in the corresponding parental cells (*P* < 0.001, Fig. 4d).

To verify whether miR-224-3p targets a site within 3′-UTR of MTDH in ESCC, we constructed luciferase reporters containing a WT or Mut MTDH-3′-UTR. As 4 sites in the 3′-UTR of MTDH were identified to be potentially capable of binding to miR-224-3p, we constructed 4 different mutant sequences (e.g. MTDH-3′-UTR-Mut#1, MTDH-3′-UTR-Mut#2, MTDH-3′-UTR-Mut#3 and MTDH-3′-UTR-Mut#4) (Fig. 4e & Additional file 2: Fig. S5c). Then, the reporters carrying MTDH-3′-UTR-WT or MTDH-3′-UTR-Mut were introduced into KYSE30/CDDP-R and TE1/CDDP-R cells and cotransfected with miR-224-3p mimics. Co-transfection of the miR-224-3p mimics significantly reduced the luciferase activity of MTDH in the cells transfected with the reporter carrying MTDH-3′-UTR-WT, MTDH-3′-UTR-Mut#2, MTDH-3′-UTR-Mut#3 or MTDH-3′-UTR-Mut#4, but did not reduce that in the cells transfected with the reporter carrying MTDH-3′-UTR-Mut#1 (Fig. 4f & Additional file 2: Fig. S5d). The above results indicated that miR-224-3p bound to site #1 in the 3′-UTR of MTDH in ESCC cells. Furthermore, we detected the expression of MTDH in above ESCC cells at the protein level by performing western blotting. The results showed that the miR-224-3p mimic significantly decreased the expression of MTDH in KYSE30/CDDP-R and TE1/CDDP-R cells transfected with a reporter carrying MTDH-3′-UTR-WT, MTDH-3′-UTR-Mut#2, MTDH-3′-UTR-Mut#3 or MTDH-3′-UTR-Mut#4, but it had no similar effect on the cells transfected with the reporter carrying MTDH-3′-UTR-Mut#1 (Fig. 4g & Additional file 2: Fig. S5e). These results indicated that miR-224-3p could regulate the expression of MTDH in ESCC cells. Thus, we hypothesized that NORAD sponged miR-224-3p to regulate MTDH in ESCC cells.

Next, we aimed to determine whether NORAD upregulated MTDH by sponging miR-224-3p in ESCC cells. Thus, we knocked down NORAD with shRNA and co-transfected a miR-224-3p inhibitor into KYSE30/CDDP-R and TE1/CDDP-R cells. NORAD knockdown significantly decreased the expression of MTDH in KYSE30/CDDP-R and TE1/CDDP-R cells, and the miR-224-3p inhibitor rescued this downregulation (Fig. 4h & Additional file 2: Fig. S5f). In addition, we overexpressed NORAD from a plasmid and co-transfected the miR-224-3p mimic into TE1 and KYSE30 cells simultaneously. Consequently, overexpression of NORAD significantly increased the expression of MTDH in KYSE30 and TE1 cells, and the miR-224-3p mimic partially rescued this upregulation (Fig. 4i & Additional file 2: Fig. S5g). Next, we analysed the correlations among NORAD, miR-224-3p and MTDH in our collected ESCC specimens. The results showed that miR-224-3p was negatively correlated with NORAD and MTDH (Pearson R = − 0.389 and − 0.706, both *P* < 0.001) and that NORAD was positively correlated with MTDH (Pearson R = 0.541, *P* < 0.001) in ESCC specimens (Fig. 4j). In summary, these results verified the existence of a NORAD/miR-224-3p/MTDH axis, which might contribute to CDDP resistance of ESCC cells.

### The NORAD/miR-224-3p/MTDH axis promotes CDDP resistance and progression in ESCC cells through regulating activation of β-catenin

**Fig. 5:**
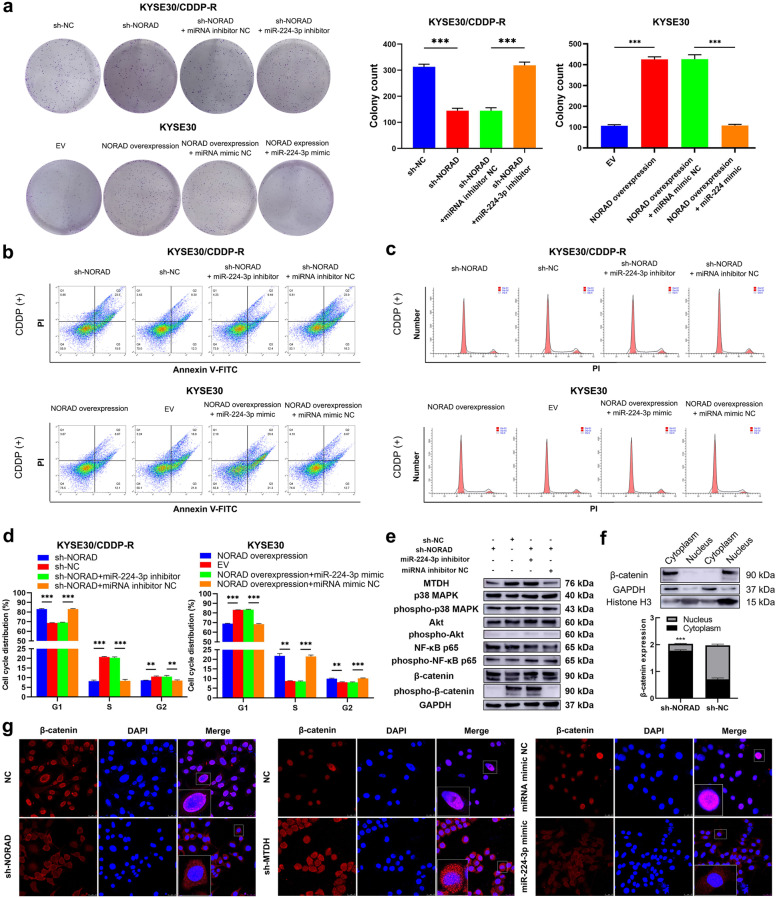
The NORAD/miR-224-3p/MTDH axis promotes CDDP resistance in ESCC cells by promoting nuclear accumulation of β-catenin. **a** The effects of NORAD/miR-224-3p/MTDH axis on the colony formation ability of KYSE30/CDDP-R and KYSE30 cells in the presence of CDDP. **b** The effects of NORAD/miR-224-3p/MTDH axis on CDDP induced apoptosis of KYSE30/CDDP-R and KYSE30 cells. **c** NORAD knockdown facilitates CDDP to induce cell cycle arrest in KYSE30/CDDP-R cells while miR-224-3p inhibitor rescued the arrest. **d** Overexpression of NORAD suppresses CDDP-induced cell cycle arrest in KYSE30 cells while miR-224-3p mimic neutralizes the suppression. **e** The effects of NORAD/miR-224-3p/MTDH axis on the expression and phosphorylation of MAPK, Akt, NF-κB and β-catenin in KYSE30/CDDP-R cells. **f** NORAD knockdown decreases the nuclear expression of MTDH in KYSE30/CDDP-R cells, detected by western blotting. **g** NORAD knockdown decreases the nuclear expression of MTDH in KYSE30/CDDP-R cells, detected by immunofluorescence. In all cases, error bars denote SD of triplicates. **P < 0.01, ***P < 0.001

Then, we treated KYSE30/CDDP-R and TE1/CDDP-R cells with CDDP to determine the effect of the NORAD/miR-224-3p/MTDH axis in ESCC cells. A colony formation assay showed that knocking down NORAD partially abolished the CDDP resistance of KYSE30/CDDP-R and TE1/CDDP-R cells while the miR-224-3p inhibitor rescued this effect (Fig. 5a & Additional file 2: Fig. S7a). Additionally, the sensitivity of KYSE30 and TE1 cells to CDDP was decreased by overexpression of NORAD, while the miR-224-3p mimic neutralized this phenomenon (Fig. 5a & Additional file 2: Fig. S7a). Furthermore, the CDDP-induced apoptosis rates of KYSE30/CDDP-R and TE1/CDDP-R cells were increased by knocking down NORAD and then rescued by co-transfection of the miR-224-3p inhibitor (Fig. 5b & Additional file 2: Fig. S7b). Similarly, the CDDP-induced apoptosis rates of TE1 and KYSE30 cells were decreased by overexpression of NORAD and then rescued by the miR-224-3p mimic (Fig. 5b & Additional file 2: Fig. S7b). In addition, knockdown of NORAD resulted in an increase of CDDP-induced G1 phase arrest in KYSE30/CDDP-R and TE1/CDDP-R cells while miR-224-3p inhibitor rescued the increase (Fig. 5c-d & Additional file 2: Fig. S7c-d). Similarly, miR-224-3p mimic partly neutralized the effect of NORAD overexpression on CDDP-induced G1 phase arrest in KYSE30 and TE1 cells (Fig. 5c-d & Additional file 2: Fig. S7c-d). These results demonstrated that the NORAD/miR-224-3p/MTDH axis promoted CDDP resistance in ESCC cells.

MTDH has been reported to be involved in regulating the activation of several key proteins involved in signalling pathways including Akt, MAPK, NF-κB and β-catenin, which possibly contribute to chemotherapy resistance in cancers [28, 29]. Thus, we detected the expression of Akt, MAPK, NF-κB, β-catenin and their phosphorylation status in the ESCC cells to identify the potential downstream signalling pathway of NORAD/miR-224-3p/MTDH axis. Western blotting analysis demonstrated that transfection of NORAD and the miR-224-3p mimic did not affect the expression of Akt, MAPK, or NF-κB or their phosphorylation status in KYSE30/CDDP-R and TE1/CDDP-R cells (Fig. 5e & Additional file 2: Fig. S7c). Additionally, knocking down of NORAD downregulated phospho-β-catenin without any effect on β-catenin, and the miR-224-3p inhibitor neutralized the above alteration in KYSE30/CDDP-R and TE1/CDDP-R cells (Fig. 5e & Additional file 2: Fig. S7c). These results indicated that the NORAD/miR-224-3p/MTDH axis might promote CDDP resistance in ESCC by activating β-catenin signalling rather than Akt, MAPK or NF-κB. Then, we studied the effect of MTDH on the activation of β-catenin to further reveal the precise mechanism involving the NORAD/miR-224-3p/MTDH axis in CDDP resistance in ESCC. As nuclear accumulation is essential for the activation of β-catenin and MTDH can promote this process [30], we next analysed the effect of NORAD on the subcellular location of β-catenin in ESCC cells. Western blotting analysis showed that knocking down NORAD significantly decreased the nuclear/cytoplasmic expression of β-catenin in KYSE30/CDDP-R (*P* < 0.001; Fig. 5f) and TE1/CDDP-R cells (*P* = 0.004; Additional file 2: Fig. S7d). In addition, we performed immunofluorescence staining to observe the effect of the NORAD/miR-224-3p/MTDH axis on the subcellular location of β-catenin in ESCC cells. Consistent with the western blotting results, knocking down NORAD or MTDH and co-transfecting the miR-224-3p mimic decreased the nuclear accumulation of β-catenin in KYSE30/CDDP-R and TE1/CDDP-R cells (Fig. 5g & Additional file 2: Fig. S7e). Thus, NORAD/miR-224-3p/MTDH axis might contribute to CDDP resistance in ESCC cells by promoting nuclear accumulation of β-catenin.

**Fig. 6:**
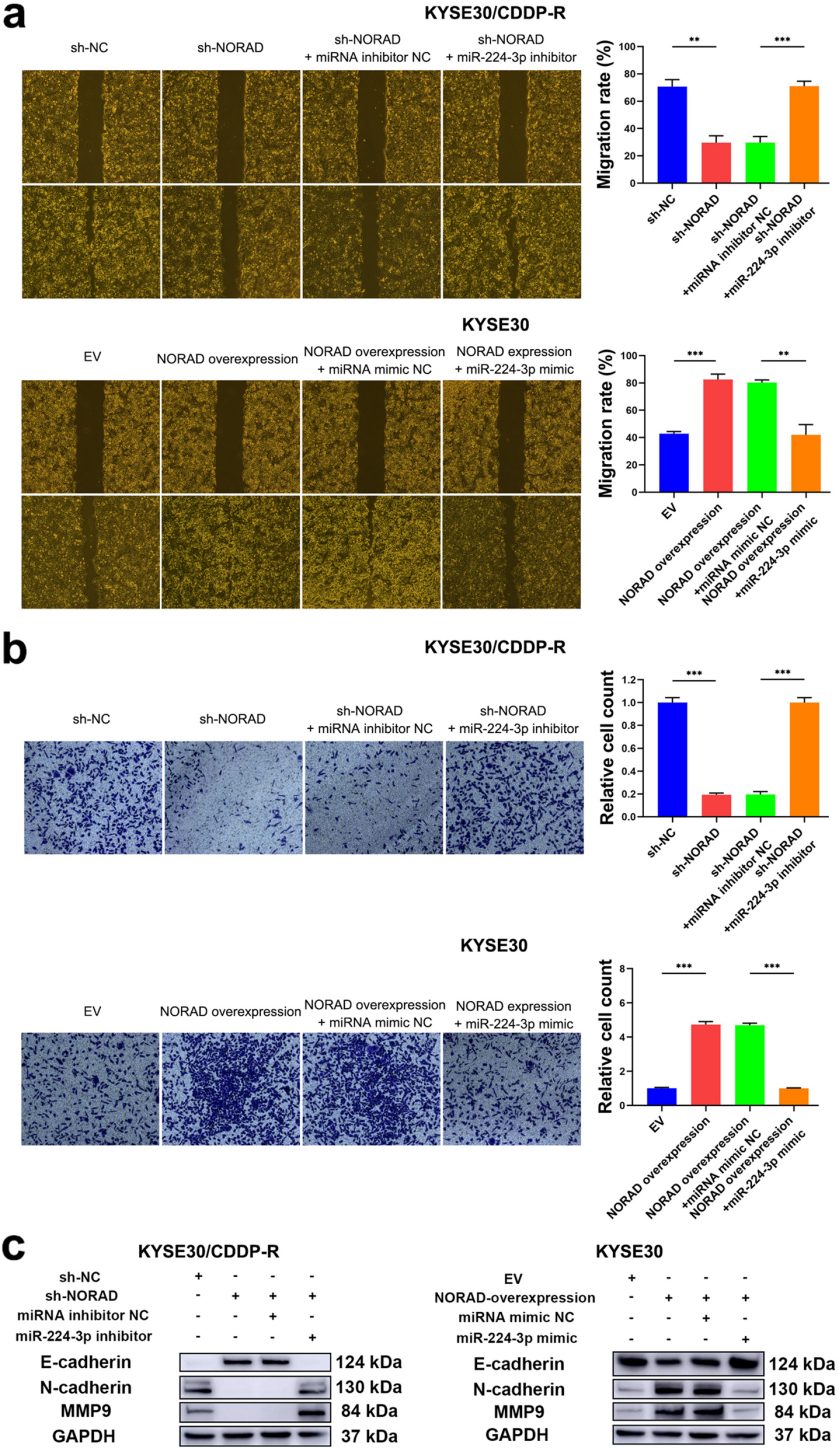
The NORAD/miR-224-3p/MTDH axis promotes progression of ESCC cells. **a** The effects of the NORAD/miR-224-3p/MTDH axis on migratory ability of KYSE30/CDDP-R and KYSE30 cells. **b** The effects of the NORAD/miR-224-3p/MTDH axis on invasive ability of KYSE30/CDDP-R and KYSE30 cells. **c** The effects of the NORAD/miR-224-3p/MTDH axis on the expression of E-cadherin, N-cadherin and MMP9 in KYSE30/CDDP-R and KYSE30 cells. In all cases, error bars denote SD of triplicates. **P < 0.01, ***P < 0.001

As β-catenin can promote the migration and invasion of ESCC cells [31], we then determined the effect of the NORAD/miR-224-3p/MTDH axis on malignant behaviours involved in tumorigenesis. As expected, the wound healing assay demonstrated that knocking down NORAD suppressed the migration of KYSE30/CDDP-R and TE1/CDDP-R cells, while the miR-224-3p inhibitor attenuated this suppression (Fig. 6a & Additional file 2: Fig. S8a). Moreover, the Transwell Matrigel assay showed that knocking down NORAD reduced the invasive ability of KYSE30/CDDP-R and TE1/CDDP-R cells, and this reduction could also be neutralized by the miR-224-3p inhibitor (Fig. 6b & Additional file 2: Fig. S8b). In addition, overexpression of NORAD in KYSE30 and TE1 cells further enhanced their migratory and invasive abilities (Fig. 6a-b & Additional file: Fig. S8a-b). Furthermore, we assayed the effect of NORAD on the expression of migration and invasion-related factors including E-cadherin, N-cadherin and matrix metalloproteinase 9 (MMP9). As a result, knocking down NORAD increased the expression of E-cadherin and decreased the expression of N-cadherin and MMP9 in KYSE30/CDDP-R and TE1/CDDP-R cells, while the miR-224-3p inhibitor neutralized these alterations (Fig. 6c & Additional file: Fig. S8c). In addition, overexpression of NORAD decreased the expression of E-cadherin and increased the expression of N-cadherin and MMP9 in KYSE30 and TE1 cells, while the miR-224-3p mimic abolished these changes (Fig. 6c & Additional file: Fig. S8c). Taken together, these results demonstrated that the NORAD/miR-224-3p/MTDH axis promoted nuclear accumulation of β-catenin to activate this protein, and thus contributed to CDDP resistance and progression in ESCC cells.

### NORAD promotes CDDP resistance and growth in ESCC in vivo

**Fig. 7:**
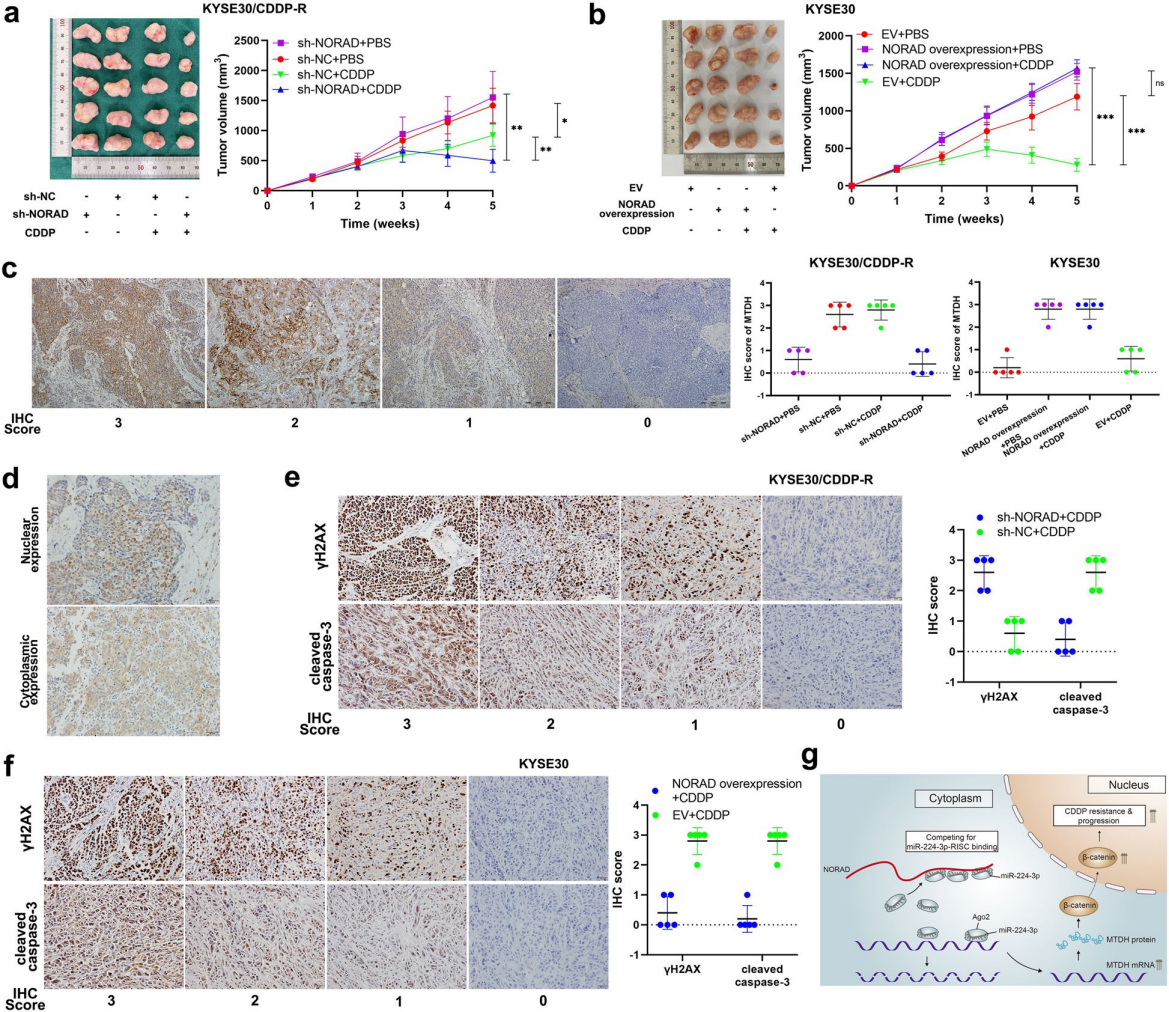
The NORAD/miR-224-3p/MTDH axis contributes to CDDP resistance of ESCC in vivo. **a** KYSE30/CDDP-R cell-formed xenograft tumors of sacrificed mice with or without CDDP treatment (3 mg/kg, three times a week) at the end of the experiment and their growth curves. **b** KYSE30 cell-formed xenograft tumors of sacrificed mice with or without CDDP treatment (3 mg/kg, three times a week) at the end of the experiment and their growth curves. **c** Representative images of IHC staining of MTDH in xenograft tumors (× 100). Scale bars, 200 μm. **d** Representative images of nuclear and cytoplasmic expression of MTDH in xenograft tumors, detected by IHC (× 200). Scale bar, 20 μm. **e** Representative images of CDDP-induced γH2AX and cleaved caspase-3 in KYSE30/CDDP-R cell-formed xenograft tumors, detected by IHC (× 200). Scale bar, 20 μm. **f** IHC scores of γH2AX and cleaved caspase-3 in KYSE30/CDDP-R cell-formed xenograft tumors. **g** The summary figure showing the effect of the NORAD/miR-224-3p/MTDH axis in ESCC. Error bars indicate SD. *P < 0.05, **P < 0.01, ***P < 0.001

To determine the effect of NORAD on CDDP resistance and tumour growth in ESCC in vivo, 40 female BALB/c nude mice were used to establish a xenograft tumour model. The 40 nude mice were randomly divided into 8 groups, among which 4 groups were injected with KYSE30/CDDP-R cells and the other 4 groups were injected with KYSE30 cells. The KYSE30/CDDP-R cells used for injection were previously treated with sh-NORAD or sh-NC, and the KYSE30 cells were previously treated with a NORAD overexpression vector or EV. ESCC cells were injected into the dorsal flank of the mice to form xenograft tumours. One week later, we intraperitoneally injected the tumour-bearing mice with the indicated concentration of CDDP or PBS. The xenograft tumours derived from KYSE30/CDDP-R cells grew continuously when they were treated with CDDP, although the tumour volumes were smaller than those of KYSE30/CDDP-R cells-derived tumours treated with PBS. In contrast, CDDP induced significant regression in tumours derived from KYSE30/CDDP-R cells pretreated with sh-NORAD beginning in the 4th week, indicating that NORAD knockdown partly reversed CDDP resistance (Fig. 7a). Furthermore, KYSE30 cells pretreated with the NORAD overexpression vector formed continuously growing tumours even when they were treated with CDDP. The xenograft tumour volumes of KYSE30 cells with NORAD overexpression were significantly larger than those of KYSE30 cells pretreated with the EV (Fig. 7b). Notably, although NORAD knockdown did not affect the volume of KYSE30/CDDP-R tumours, NORAD overexpression increased that of KYSE30 tumours (Fig. 7a-b). The above results indicated that NORAD promoted CDDP resistance and progression in KYSE30/CDDP-R cells in vivo. Then, we detected the expression of MTDH in xenograft tumours by IHC. Consistent with the IHC results for ESCC tissues, MTDH staining was predominantly found in the cytoplasm (Fig. 7c). The expressions of MTDH in xenograft tumours derived from KYSE30/CDDP-R cells with sh-NORAD were lower than those cells with sh-NC (Fig. 7c). Similarly, the expressions of MTDH in xenograft tumours derived from KYSE30 cells with NORAD overexpression were higher than those cells with EV (Fig. 7c). Furthermore, we used IHC to detect the subcellular location of β-catenin in xenograft tumours to analyse its correlation with NORAD. We found that the nuclear expression of β-catenin was positively correlated with MTDH in ESCC cells (Fig. 7d). Furthermore, the expression of CDDP-induced γH2AX and cleaved caspase-3 were negatively correlated with MTDH in xenograft tumours (Fig. 7e-f). Taken together, these results demonstrated that NORAD contributed to progression and CDDP resistance in ESCC cells by promoting nuclear accumulation of β-catenin (Fig. 7g).

## Discussion

CDDP is a widely applied chemotherapeutic agent that exerts cytotoxic activity by binding to nuclear DNA of cancer cells with high affinity to form DNA adducts [7]. Currently, CDDP-based regimens are the most predominant chemotherapeutic strategies for treating ESCC [32]. However, CDDP does not achieve satisfactory therapeutic effects in a large proportion of ESCC patients due to primary or acquired drug resistance [33]. Thus, identifying the key factors driving drug resistance, especially epigenetic modifications, can provide pivotal information for overcoming CDDP resistance in ESCC. In this study, we analysed a public GEO dataset to verify the key lncRNAs involved in CDDP resistance in ESCC. After assaying the candidate upregulated lncRNAs in collected ESCC specimens, we ultimately focused on NORAD, which is a highly conserved and abundant cytoplasmic lncRNA [34]. NORAD, mainly induced by DNA damage, was initially identified as a lncRNA that played crucial roles in DNA protection and chromosomal stability [35]. Recent studies have demonstrated that NORAD acts as an oncogene in multiple cancers including malignant melanoma, pancreatic cancer, NSCLC and glioblastoma [36–39]. Importantly, NORAD has been reported to be correlated with chemotherapy resistance in cancer cells, such as resistance to doxorubicin, CDDP and oxaliplatin in neuroblastoma, NSCLC and gastric cancer, respectively [40–42]. Regarding ESCC, although NORAD expression has been reported to be significantly related with a poor post-operative prognosis [43], whether and how NORAD contributes to CDDP resistance remain to be elucidated.

In this study, we detected the expression of NORAD in ESCC specimens and analysed the correlation of NORAD with CDDP resistance. The expression of NORAD was significantly higher in CDDP-resistant ESCC patients than in CDDP-sensitive patients, indicating that NORAD was potentially correlated with CDDP resistance. Furthermore, the expression of NORAD was positively correlated with the postoperative DFS and OS of ESCC patients. Since these patients all received CDDP-based regimens as their postoperative adjuvant therapy, this result provided further evidence that NORAD was associated with CDDP resistance. Based on the above clinical significance, we established CDDP-resistant ESCC cell lines to further investigate the possible functional mechanisms of NORAD. As expected, the expression of NORAD was significantly upregulated in CDDP-resistant ESCC cells, compared to matched parental cells. Through gain and loss-of-function experiments performed in vivo and in vitro, we found that NORAD indeed promoted CDDP resistance in ESCC. To our knowledge, this study is the first to show the key role of NORAD in CDDP resistance in ESCC.

The functions of lncRNAs partially depend on their precise subcellular location [44]. Some published studies have revealed that NORAD is mainly located in the cytoplasm [45, 46]. Aiming to determine the functional mechanisms of NORAD, we first detected its subcellular location in ESCC cells and found that it was dominantly located in the cytoplasm, which was consistent with previous studies. For cytoplasmic lncRNAs, acting as ceRNAs to sponge miRNAs is the most frequent and important posttranscriptional mechanism through which lncRNAs can regulate chemotherapy-related target genes [44, 47]. For instance, the lncRNA growth arrest-specific 5 (GAS5) can promote the expression of programmed cell death 4 (PDCD4) by sponging miR-21 and thereby enhance the CDDP sensitivity of cervical cancer cells [48]. In this study, we analysed another public GEO dataset and found 3 downregulated miRNAs possibly involved in CDDP resistance, among which only miR-224-3p exhibited the potential to bind to NORAD. MiR-224-3p can act as either an oncogene or a tumour-suppressor in different cancers, due to its precise target genes. In NSCLC, miR-224-3p promotes stem cell-like properties by downregulating TP53 and TET1 [49]. In contrast, miR-224-3p abolishes the tumour-promoting effects of the lncRNA small nucleolar RNA host gene 4 (SNHG4) in osteosarcoma [50]. In ESCC, the expression, biological function and clinical significance of miR-224-3p have not been reported. Herein, we found that miR-224-3p was negatively correlated with NORAD in ESCC specimens, indicating possible binding between NORAD and miR-224-3p. Furthermore, we confirmed the interaction between NORAD and miR-224-3p in CDDP-resistant ESCC cells by conducting qRT-PCR and luciferase reporter assays. Moreover, NORAD and miR-224-3p occupied the same Ago2 site to form a RISC in ESCC cells, suggesting that their interaction could affect the expression of downstream mRNAs. Therefore, we performed analyses with bioinformatic algorithms and a public GEO dataset to predict the latent downstream genes. After overlapping the results, only MTDH exhibited the potential to be a miR-224-3p-regulated gene involved in CDDP-resistance in ESCC.

The MTDH gene is located on human chromosome 8q22 and contains 12 exons and 11 introns [51]. Since the functional domains have not been precisely defined due to the complicated crystal structure, the precise functions of MTDH remain elusive [52]. The predominant subcellular locations of MTDH differ according to tissue and cell type. Generally, MTDH is predominantly located in the nucleus in benign tissues and cells, while it is located mainly in the cytoplasm in cancer tissues and cells [52]. The expression of MTDH in cancer cells is regulated by diverse mechanisms including miRNA-mediated post-transcriptional processes [53]. For instance, downregulation of MTDH expression is induced by miR-375 and results in reduced growth of hepatocellular carcinoma cells [54]. In this study, we identified a NORAD/miR-224-3p/MTDH axis in ESCC by performing miRNA rescue experiments. Furthermore, we confirmed that the NORAD/miR-224-3p/MTDH axis promoted CDDP resistance in ESCC cells by conducting gain and loss-of-function experiments. MTDH has been confirmed to activate numerous cancer-related signalling pathways that potentially contribute to CDDP resistance [52]. In this study, we determined that MTDH mainly promoted the activation of β-catenin in ESCC whereas it had no significant effect on NF-κB, Akt or MAPK. Regarding the precise mechanism, MTDH promoted nuclear accumulation of β-catenin to sustain a sufficient phosphorylation status in ESCC cells. Therefore, our findings not only present a novel mechanism by which β-catenin was activated to promote CDDP resistance in ESCC, but also highlight the related upstream lncRNA-involved regulatory axis. Consequently, to explore the potential translational value of NORAD, we established a xenograft tumour mouse model to verify whether NORAD contributed to CDDP resistance in ESCC in vivo. As anticipated, NORAD knockdown and overexpression significantly reduced and enhanced, respectively, CDDP resistance in ESCC cells in vivo, indicating that NORAD might be a novel target for overcoming CDDP resistance in ESCC.

## Conclusion

In this study, we identified NORAD as a key lncRNA involved in CDDP resistance in ESCC both in vitro and in vivo. We found that NORAD was significantly correlated with poor DFS and OS in ESCC patients who received CDDP-based chemotherapy regimens as an adjuvant therapy. NORAD sponged miR-224-3p to upregulate MTDH in CDDP-resistant ESCC cells. The NORAD/miR-224-3p/MTDH axis contributed to CDDP resistance by promoting nuclear accumulation of β-catenin. NORAD knockdown significantly sensitized ESCC cells to CDDP. Our findings shed new light on an MTDH-targeted therapeutic approach that has clinical potential to reverse CDDP resistance and achieve better clinical outcomes in ESCC patients.

## Supplementary Information

**Additional file 1: Table S1.** Primer sequences for qRT-PCR. **Table S2.** Primer sequences for qRT-PCR. **Table S3.** The sequences of shRNA for NORAD. **Table S4.** Sequences of miR-224-3p mimic and inhibitor.

**Additional file 2: Figure S1.** The effect of CDDP on cell viability of ESCC cells. **a** The effect of CDDP on cell viability of KYSE30/CDDP-R and KYSE30 cells. **b** The effect of CDDP on cell viability of TE1/CDDP-R and TE1 cells. **Figure S2.** Representative images of RNA FISH of NORAD in TE1/CDDP-R and TE1 cells (× 1000), which show that NORAD is predominantly located in the cytoplasm. Nuclei are stained with DAPI. **Figure S3.** The effect of sh-NORAD and overexpression vector on the expression of NORAD and proliferation ability of ESCC cells. **a** The effect of sh-NORAD on NORAD expression in KYSE30/CDDP-R and TE1/CDDP-R cells. Error bars denote SD of triplicates. ***P < 0.001. **b** The effect of overexpression of NORAD on NORAD expression in KYSE30 and TE1 cells. Error bars denote SD of triplicates. ***P < 0.001. **c** The effect of sh-NORAD on proliferation ability of KYSE30/CDDP-R and TE1/CDDP-R cells. **d** The effect of overexpression of NORAD on proliferation ability of KYSE30 and TE1 cells. The results are presented as the mean ± SD. ***P < 0.001. **Figure S4.** NORAD contributes to CDDP resistance of TE1 cells. **a** NORAD knockdown increases the sensitivity of TE1/CDDP-R cells to CDDP, detected by CCK-8. **b** Overexpression of NORAD decreases the sensitivity of TE1/CDDP-R cells to CDDP, detected by CCK-8. **c** NORAD knockdown decreases the colony formation ability of TE1/CDDP-R cells in the presence of CDDP. **d** Overexpression of NORAD increases the colony formation ability of TE1 cells in the presence of CDDP. **e** NORAD knockdown increases the CDDP-induced apoptosis rate of TE1/CDDP-R cells, and overexpression of NORAD decreases the CDDP-induced apoptosis rate of TE1 cells, detected by FCM. **f** NORAD knockdown facilitates CDDP to induce cell cycle arrest in TE1/CDDP-R cells. **g** Overexpression of NORAD suppresses CDDP-induced cell cycle arrest in TE1 cells. **h** NORAD knockdown increases the CDDP-induced γH2AX and cleaved caspase-3 in TE1/CDDP-R cells. **i** Overexpression of NORAD decreases the CDDP-induced γH2AX and cleaved caspase-3 of TE1 cells. In all cases, error bars denote SD of triplicates. ***P < 0.001. **Figure S5.** MTDH is a direct target of miR-224-3p in TE1 cells. **a** Representative images of RNA FISH of NORAD and miR-224-3p in TE1/CDDP-R cells, which show that NORAD and miR-224-3p are co-located in the cytoplasm. Nuclei are stained with DAPI. **b** The nuclear and cytoplasmic expression of NORAD and miR-224-3p in TE1/CDDP-R cells. **c** The structure of the luciferase reporter plasmid. The sequences of MTDH-3′-UTR-WT or MTDH-3′-UTR-Mut were subcloned into the cloning region. **d** Luciferase reporter assay showing the luciferase activity of MTDH-3′-UTR-WT, MTDH-3′-UTR-Mut#1, MTDH-3′-UTR-Mut#2, MTDH-3′-UTR-Mut#3 and MTDH-3′-UTR-Mut#4 in TE1/CDDP-R cells co-transfected with miR-224-3p mimic. **e** The effects of miR-224-3p mimic on the expression of MTDH in TE1/CDDP-R cells transfected with MTDH-3′-UTR-WT, MTDH-3′-UTR-Mut#1, MTDH-3′-UTR-Mut#2, MTDH-3′-UTR-Mut#3 or MTDH-3′-UTR-Mut#4. **f** NORAD knockdown downregulates MTDH expression in TE1/CDDP-R cells and miR-224-3p mimic rescues this downregulation. **g** Overexpression of NORAD upregulates MTDH expression in TE1 cells and miR-224-3p inhibitor rescues this downregulation. **b-e** Error bars denote SD of triplicates. ***P < 0.001. **Figure S6. a** Predicted binding sites on NORAD to sponge miR-224-3p and matched established mutant sequences for luciferase reporter assay. **b** The structure of the luciferase reporter plasmid. The sequences of NORAD-WT or NORAD-Mut were subcloned into the cloning region. **Figure S7.** The NORAD/miR-224-3p/MTDH axis promotes CDDP resistance in TE1 cells by promoting nuclear accumulation of β-catenin. **a** The effects of NORAD/miR-224-3p/MTDH axis on the colony formation ability of TE1/CDDP-R and TE1 cells in the presence of CDDP. **b** The effects of NORAD/miR-224-3p/MTDH axis on CDDP induced apoptosis of TE1/CDDP-R and TE1 cells. **c** NORAD knockdown facilitates CDDP to induce cell cycle arrest in TE1/CDDP-R cells while miR-224-3p inhibitor rescued the arrest. **d** Overexpression of NORAD suppresses CDDP-induced cell cycle arrest in TE1 cells while miR-224-3p mimic neutralizes the suppression. **e** The effects of NORAD/miR-224-3p/MTDH axis on the expression and phosphorylation of MAPK, Akt, NF-κB and β-catenin in TE1/CDDP-R cells. **f** NORAD knockdown decreases the nuclear expression of MTDH in TE1/CDDP-R cells, detected by western blotting. **g** NORAD knockdown decreases the nuclear expression of MTDH in TE1/CDDP-R cells, detected by immunofluorescence. In all cases, error bars denote SD of triplicates. **P < 0.01, ***P < 0.001. **Figure S8**. The NORAD/miR-224-3p/MTDH axis promotes progression of TE1 cells. **a** The effects of the NORAD/miR-224-3p/MTDH axis on migratory ability of TE1/CDDP-R and TE1 cells. **b** The effects of the NORAD/miR-224-3p/MTDH axis on invasive ability of TE1/CDDP-R and TE1 cells. **c** The effects of the NORAD/miR-224-3p/MTDH axis on the expression of E-cadherin, N-cadherin and MMP9 in TE1/CDDP-R and TE1 cells. In all cases, error bars denote SD of triplicates. **P < 0.01, ***P < 0.001.

**Additional file 3.**

**Additional file 4.**

## Data Availability

All data and materials in our study are available upon reasonable request.

## Abbreviations

ESCC: Esophageal squamous cell carcinoma
CDDP: Cis–diamminedichloro-platinum
lncRNA: Long non-coding RNA
ceRNA: Competing endogenous RNA
miRNA: microRNA
UTR: Untranslated region
NSCLC: Non-small cell lung cancer
NORAD: Non-coding RNA activated by DNA damage
MTDH: Metadherin
UICC: Union for International Cancer Control
FBS: Fetal bovine serum
shRNA: Short hairpin RNA
FISH: Fluorescence in situ hybridization
EV: Empty vector
Ago2: Argonaute-2
IHC: Immunohistochemistry
CCK-8: Cell counting kit-8
DFS: Disease-free survival
OS: Overall survival
ROC: Receiver operating characteristic curve
AUC: Area under the ROC
IC_50_: half maximal inhibitory concentration
RISC: RNA-induced silencing complex
