# Supplementary material for: Long non-coding RNA NORAD/miR-224-3p/MTDH axis contributes to CDDP resistance of esophageal squamous cell carcinoma by promoting nuclear accumulation of β-catenin
Source: Mol Cancer. 2021 Dec 10;20:162. doi: 10.1186/s12943-021-01455-y (PMC8662861; doi:10.1186/s12943-021-01455-y)
Supplement: Supplementary file 1 — Additional file 1: Table S1. Primer sequences for qRT-PCR. Table S2. Primer sequences for qRT-PCR. Table S3. The sequences of shRNA for NORAD. Table S4. Sequences of miR-224-3p mimic and inhibitor. [file 12943_2021_1455_MOESM1_ESM.zip › Table S1.docx]

Table S1 Primer sequences for qRT-PCR

| Name | RefSeq Number in NCBI | Primer sequence | Product  Size (bp) |
| --- | --- | --- | --- |
| NORAD | NR_027451.1 | F: 5’-GTGACCACTCTGTCGCCATT-3’ | 144 |
|  |  | R: 5’-AGAATGAAGACCAACCGCCC-3’ |  |
| HOXA-AS2 | NR_122069.1 | F: 5’-AACCACGCTTTTCCCGTAGG-3’ | 176 |
|  |  | R: 5’-GGTAAGCGCTGCTCCAAAAC-3’ |  |
| linc00996 | NR_034033.1 | F: 5’-ACTCTCTGCCACATCGTTCG-3’ | 119 |
|  |  | R: 5’-TCCTTCCGCTTCTTACGCTG-3’ |  |
| TENM3-AS1 | NR_027107.2 | F: 5’-GATGCGCAGCTCTCCAAAAG-3’ | 198 |
|  |  | R: 5’-TGTTGGCCTTGCCTCAGTAG-3’ |  |
| CEP83-DT | NR_027035.1 | F: 5’-AACAGACACCTTCCCATCCC-3’ | 175 |
|  |  | R: 5’-TGGCCCGTTTTCCCTCATTT-3’ |  |
| GAPDH | NM_002046.7 | F: 5’-ACAACTTTGGTATCGTGGAAGG-3’ | 101 |
|  |  | R: 5’-GCCATCACGCCACAGTTTC-3’ |  |
| U6 | NR_138085.1 | F: 5’-CTCGCTTCGGCAGCACATA-3’ | 94 |
|  |  | R: 5’-AACGCTTCACGAATTTGCGT-3’ |  |
