# Supplementary material for: Long non-coding RNA NORAD/miR-224-3p/MTDH axis contributes to CDDP resistance of esophageal squamous cell carcinoma by promoting nuclear accumulation of β-catenin
Source: Mol Cancer. 2021 Dec 10;20:162. doi: 10.1186/s12943-021-01455-y (PMC8662861; doi:10.1186/s12943-021-01455-y)
Supplement: Supplementary file 1 — Additional file 1: Table S1. Primer sequences for qRT-PCR. Table S2. Primer sequences for qRT-PCR. Table S3. The sequences of shRNA for NORAD. Table S4. Sequences of miR-224-3p mimic and inhibitor. [file 12943_2021_1455_MOESM1_ESM.zip › Table S2.docx]

Table S2 Primer sequences for qRT-PCR

| Name | Accession Number in miRBase | Primer sequence | Product  Size (bp) |
| --- | --- | --- | --- |
| miR-224-3p | MIMAT0009198 | F: 5’-AAAATGGTGCCCTAGTGACTACA-3’ | 94 |
|  |  | R: 5’-GAGCAGGCTGGAGAA-3’ |  |
| miR-28-5p | MIMAT0000085 | F: 5’-TGGTGTCGTGGGTCGA-3’ | 93 |
|  |  | R: 5’-CTCGCTTCGGCAGCACA-3’ |  |
| miR-7-5p | MIMAT0000252 | F: 5’-GCGCTGGAAGACTAGTGATTTTGTTGTT-3’ | 87 |
|  |  | R: 5’-AACGCTTCACGAATTTGCGT-3’ |  |
