# Supplementary material for: Long non-coding RNA NORAD/miR-224-3p/MTDH axis contributes to CDDP resistance of esophageal squamous cell carcinoma by promoting nuclear accumulation of β-catenin
Source: Mol Cancer. 2021 Dec 10;20:162. doi: 10.1186/s12943-021-01455-y (PMC8662861; doi:10.1186/s12943-021-01455-y)
Supplement: Supplementary file 1 — Additional file 1: Table S1. Primer sequences for qRT-PCR. Table S2. Primer sequences for qRT-PCR. Table S3. The sequences of shRNA for NORAD. Table S4. Sequences of miR-224-3p mimic and inhibitor. [file 12943_2021_1455_MOESM1_ESM.zip › Table S3.docx]

Table S3 The sequences of shRNA for NORAD

|  |  | Sequence |  |
| --- | --- | --- | --- |
| shRNA-1 | Forward | 5’-CCGGGCCGACGTATGATAAAATAGACTCGAGTCTATTTTATCATACGTCGGCTTTTTG-3’ |  |
|  | Reverse | 5’-AATTCAAAAAGCCGACGTATGATAAAATAGACTCGAGTCTATTTTATCATACGTCGGC-3’ |  |
| shRNA-2 | Forward | 5’-CCGGGGAAGGCCTGTGTATATAACTCGAGTTATATACACAGGCCTTCCTTTTTG-3’ |  |
|  | Reverse | 5’-AATTCAAAAAGGAAGGCCTGTGTATATAACTCGAGTTATATACACAGGCCTTCC-3’ |  |
|  |  |  |  |
