# Supplementary figures and images for: Long non-coding RNA NORAD/miR-224-3p/MTDH axis contributes to CDDP resistance of esophageal squamous cell carcinoma by promoting nuclear accumulation of β-catenin
Source: Mol Cancer. 2021 Dec 10;20:162. doi: 10.1186/s12943-021-01455-y (PMC8662861; doi:10.1186/s12943-021-01455-y)

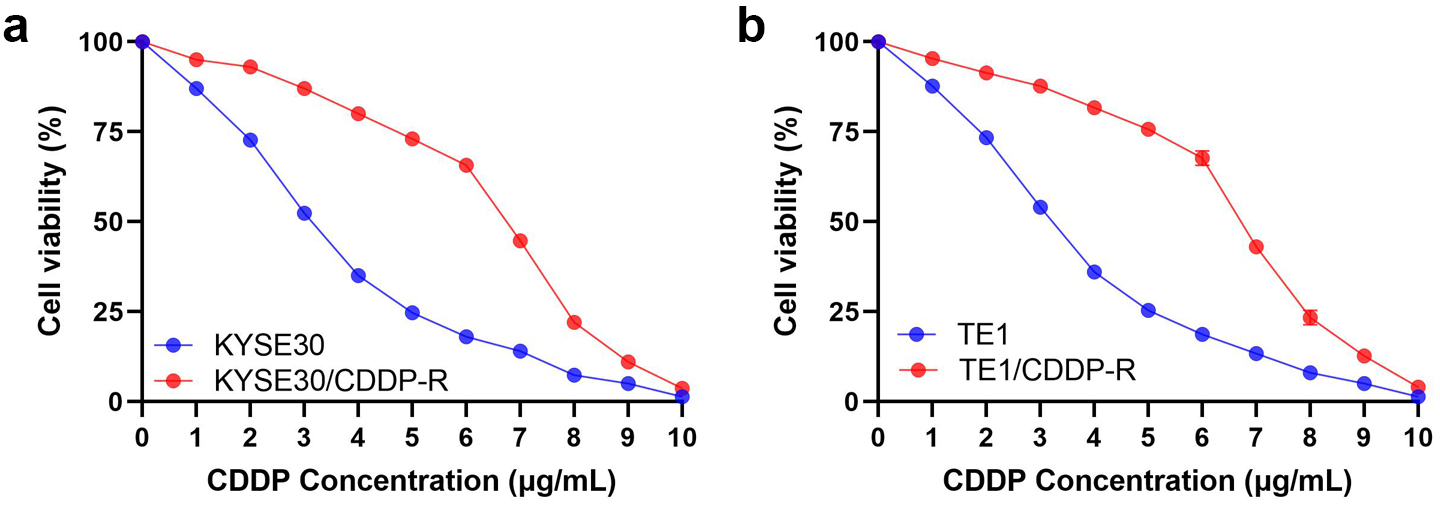

Supplement: Supplementary file 2 — Additional file 2: Figure S1. The effect of CDDP on cell viability of ESCC cells. a The effect of CDDP on cell viability of KYSE30/CDDP-R and KYSE30 cells. b The effect of CDDP on cell viability of TE1/CDDP-R and TE1 cells. Figure S2. Representative images of RNA FISH of NORAD in TE1/CDDP-R and TE1 cells (× 1000), which show that NORAD is predominantly located in the cytoplasm. Nuclei are stained with DAPI. Figure S3. The effect of sh-NORAD and overexpression vector on the expression of NORAD and proliferation ability of ESCC cells. a The effect of sh-NORAD on NORAD expression in KYSE30/CDDP-R and TE1/CDDP-R cells. Error bars denote SD of triplicates. ***P < 0.001. b The effect of overexpression of NORAD on NORAD expression in KYSE30 and TE1 cells. Error bars denote SD of triplicates. ***P < 0.001. c The effect of sh-NORAD on proliferation ability of KYSE30/CDDP-R and TE1/CDDP-R cells. d The effect of overexpression of NORAD on proliferation ability of KYSE30 and TE1 cells. The results are presented as the mean ± SD. ***P < 0.001. Figure S4. NORAD contributes to CDDP resistance of TE1 cells. a NORAD knockdown increases the sensitivity of TE1/CDDP-R cells to CDDP, detected by CCK-8. b Overexpression of NORAD decreases the sensitivity of TE1/CDDP-R cells to CDDP, detected by CCK-8. c NORAD knockdown decreases the colony formation ability of TE1/CDDP-R cells in the presence of CDDP. d Overexpression of NORAD increases the colony formation ability of TE1 cells in the presence of CDDP. e NORAD knockdown increases the CDDP-induced apoptosis rate of TE1/CDDP-R cells, and overexpression of NORAD decreases the CDDP-induced apoptosis rate of TE1 cells, detected by FCM. f NORAD knockdown facilitates CDDP to induce cell cycle arrest in TE1/CDDP-R cells. g Overexpression of NORAD suppresses CDDP-induced cell cycle arrest in TE1 cells. h NORAD knockdown increases the CDDP-induced γH2AX and cleaved caspase-3 in TE1/CDDP-R cells. i Overexpression of NORAD decreases the CDDP-induced [file 12943_2021_1455_MOESM2_ESM.zip › Figure S1.jpg]

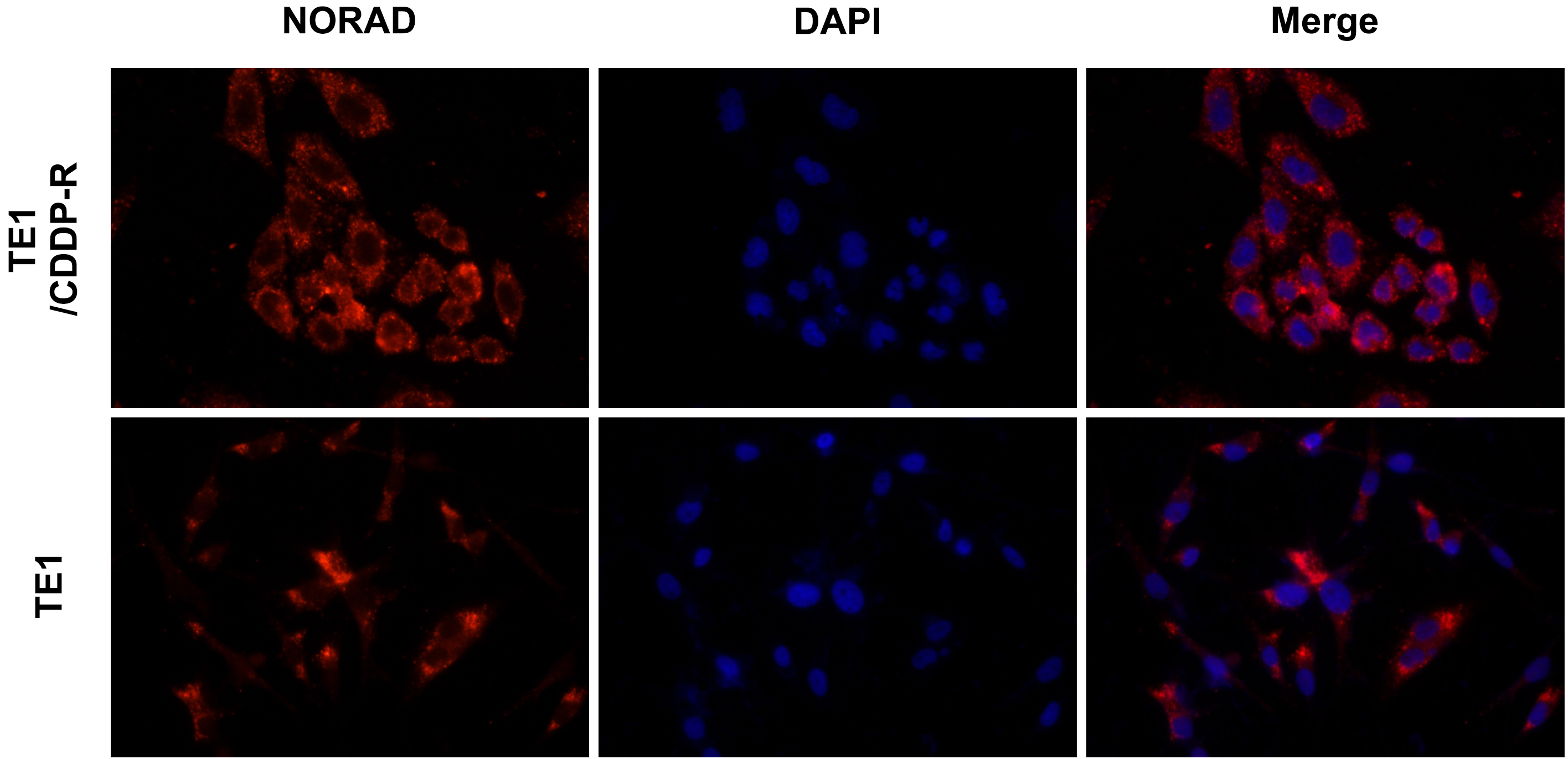

Supplement: Supplementary file 2 — Additional file 2: Figure S1. The effect of CDDP on cell viability of ESCC cells. a The effect of CDDP on cell viability of KYSE30/CDDP-R and KYSE30 cells. b The effect of CDDP on cell viability of TE1/CDDP-R and TE1 cells. Figure S2. Representative images of RNA FISH of NORAD in TE1/CDDP-R and TE1 cells (× 1000), which show that NORAD is predominantly located in the cytoplasm. Nuclei are stained with DAPI. Figure S3. The effect of sh-NORAD and overexpression vector on the expression of NORAD and proliferation ability of ESCC cells. a The effect of sh-NORAD on NORAD expression in KYSE30/CDDP-R and TE1/CDDP-R cells. Error bars denote SD of triplicates. ***P < 0.001. b The effect of overexpression of NORAD on NORAD expression in KYSE30 and TE1 cells. Error bars denote SD of triplicates. ***P < 0.001. c The effect of sh-NORAD on proliferation ability of KYSE30/CDDP-R and TE1/CDDP-R cells. d The effect of overexpression of NORAD on proliferation ability of KYSE30 and TE1 cells. The results are presented as the mean ± SD. ***P < 0.001. Figure S4. NORAD contributes to CDDP resistance of TE1 cells. a NORAD knockdown increases the sensitivity of TE1/CDDP-R cells to CDDP, detected by CCK-8. b Overexpression of NORAD decreases the sensitivity of TE1/CDDP-R cells to CDDP, detected by CCK-8. c NORAD knockdown decreases the colony formation ability of TE1/CDDP-R cells in the presence of CDDP. d Overexpression of NORAD increases the colony formation ability of TE1 cells in the presence of CDDP. e NORAD knockdown increases the CDDP-induced apoptosis rate of TE1/CDDP-R cells, and overexpression of NORAD decreases the CDDP-induced apoptosis rate of TE1 cells, detected by FCM. f NORAD knockdown facilitates CDDP to induce cell cycle arrest in TE1/CDDP-R cells. g Overexpression of NORAD suppresses CDDP-induced cell cycle arrest in TE1 cells. h NORAD knockdown increases the CDDP-induced γH2AX and cleaved caspase-3 in TE1/CDDP-R cells. i Overexpression of NORAD decreases the CDDP-induced [file 12943_2021_1455_MOESM2_ESM.zip › Figure S2.jpg]

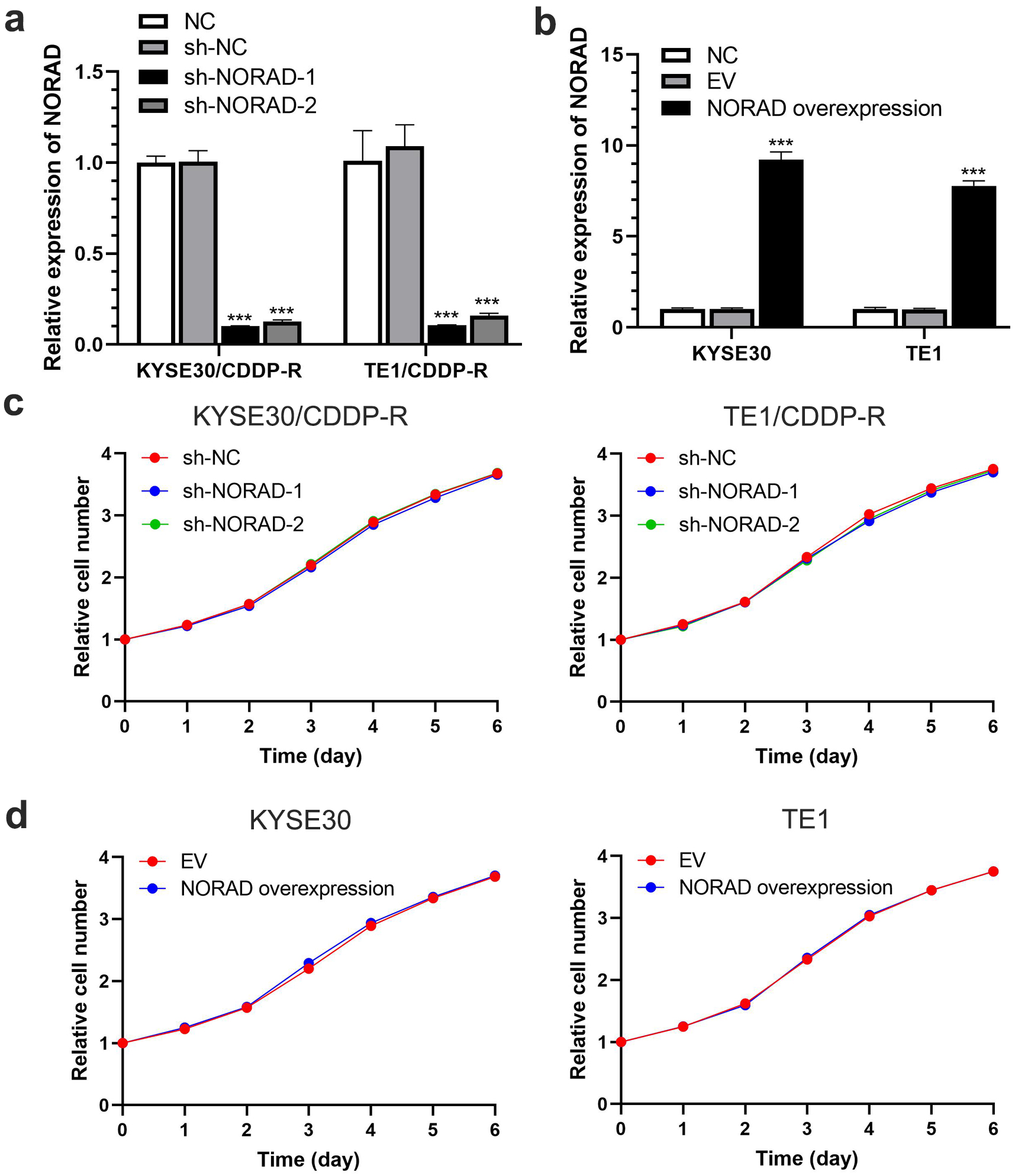

Supplement: Supplementary file 2 — Additional file 2: Figure S1. The effect of CDDP on cell viability of ESCC cells. a The effect of CDDP on cell viability of KYSE30/CDDP-R and KYSE30 cells. b The effect of CDDP on cell viability of TE1/CDDP-R and TE1 cells. Figure S2. Representative images of RNA FISH of NORAD in TE1/CDDP-R and TE1 cells (× 1000), which show that NORAD is predominantly located in the cytoplasm. Nuclei are stained with DAPI. Figure S3. The effect of sh-NORAD and overexpression vector on the expression of NORAD and proliferation ability of ESCC cells. a The effect of sh-NORAD on NORAD expression in KYSE30/CDDP-R and TE1/CDDP-R cells. Error bars denote SD of triplicates. ***P < 0.001. b The effect of overexpression of NORAD on NORAD expression in KYSE30 and TE1 cells. Error bars denote SD of triplicates. ***P < 0.001. c The effect of sh-NORAD on proliferation ability of KYSE30/CDDP-R and TE1/CDDP-R cells. d The effect of overexpression of NORAD on proliferation ability of KYSE30 and TE1 cells. The results are presented as the mean ± SD. ***P < 0.001. Figure S4. NORAD contributes to CDDP resistance of TE1 cells. a NORAD knockdown increases the sensitivity of TE1/CDDP-R cells to CDDP, detected by CCK-8. b Overexpression of NORAD decreases the sensitivity of TE1/CDDP-R cells to CDDP, detected by CCK-8. c NORAD knockdown decreases the colony formation ability of TE1/CDDP-R cells in the presence of CDDP. d Overexpression of NORAD increases the colony formation ability of TE1 cells in the presence of CDDP. e NORAD knockdown increases the CDDP-induced apoptosis rate of TE1/CDDP-R cells, and overexpression of NORAD decreases the CDDP-induced apoptosis rate of TE1 cells, detected by FCM. f NORAD knockdown facilitates CDDP to induce cell cycle arrest in TE1/CDDP-R cells. g Overexpression of NORAD suppresses CDDP-induced cell cycle arrest in TE1 cells. h NORAD knockdown increases the CDDP-induced γH2AX and cleaved caspase-3 in TE1/CDDP-R cells. i Overexpression of NORAD decreases the CDDP-induced [file 12943_2021_1455_MOESM2_ESM.zip › Figure S3.jpg]

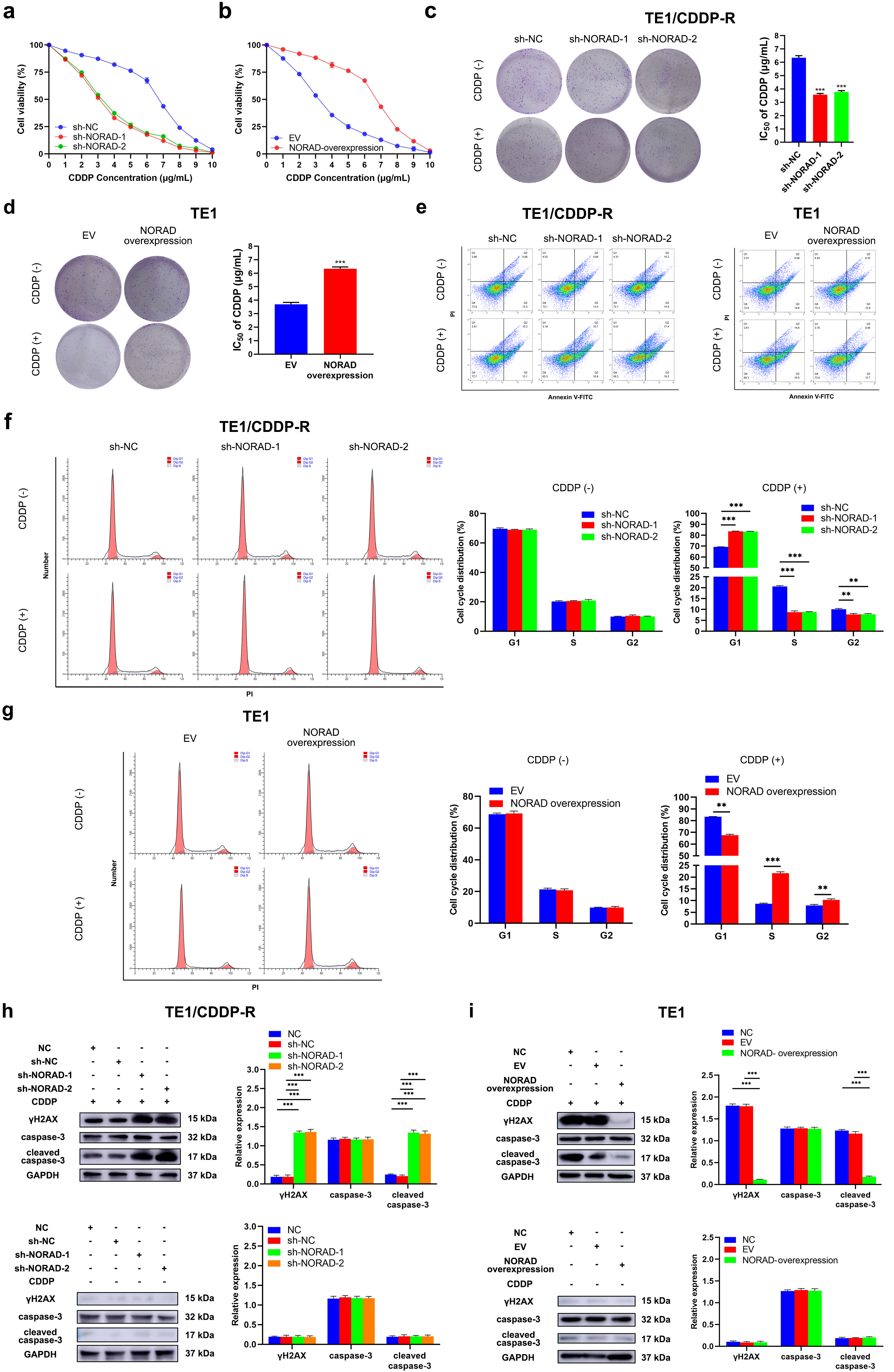

Supplement: Supplementary file 2 — Additional file 2: Figure S1. The effect of CDDP on cell viability of ESCC cells. a The effect of CDDP on cell viability of KYSE30/CDDP-R and KYSE30 cells. b The effect of CDDP on cell viability of TE1/CDDP-R and TE1 cells. Figure S2. Representative images of RNA FISH of NORAD in TE1/CDDP-R and TE1 cells (× 1000), which show that NORAD is predominantly located in the cytoplasm. Nuclei are stained with DAPI. Figure S3. The effect of sh-NORAD and overexpression vector on the expression of NORAD and proliferation ability of ESCC cells. a The effect of sh-NORAD on NORAD expression in KYSE30/CDDP-R and TE1/CDDP-R cells. Error bars denote SD of triplicates. ***P < 0.001. b The effect of overexpression of NORAD on NORAD expression in KYSE30 and TE1 cells. Error bars denote SD of triplicates. ***P < 0.001. c The effect of sh-NORAD on proliferation ability of KYSE30/CDDP-R and TE1/CDDP-R cells. d The effect of overexpression of NORAD on proliferation ability of KYSE30 and TE1 cells. The results are presented as the mean ± SD. ***P < 0.001. Figure S4. NORAD contributes to CDDP resistance of TE1 cells. a NORAD knockdown increases the sensitivity of TE1/CDDP-R cells to CDDP, detected by CCK-8. b Overexpression of NORAD decreases the sensitivity of TE1/CDDP-R cells to CDDP, detected by CCK-8. c NORAD knockdown decreases the colony formation ability of TE1/CDDP-R cells in the presence of CDDP. d Overexpression of NORAD increases the colony formation ability of TE1 cells in the presence of CDDP. e NORAD knockdown increases the CDDP-induced apoptosis rate of TE1/CDDP-R cells, and overexpression of NORAD decreases the CDDP-induced apoptosis rate of TE1 cells, detected by FCM. f NORAD knockdown facilitates CDDP to induce cell cycle arrest in TE1/CDDP-R cells. g Overexpression of NORAD suppresses CDDP-induced cell cycle arrest in TE1 cells. h NORAD knockdown increases the CDDP-induced γH2AX and cleaved caspase-3 in TE1/CDDP-R cells. i Overexpression of NORAD decreases the CDDP-induced [file 12943_2021_1455_MOESM2_ESM.zip › Figure S4.jpg]

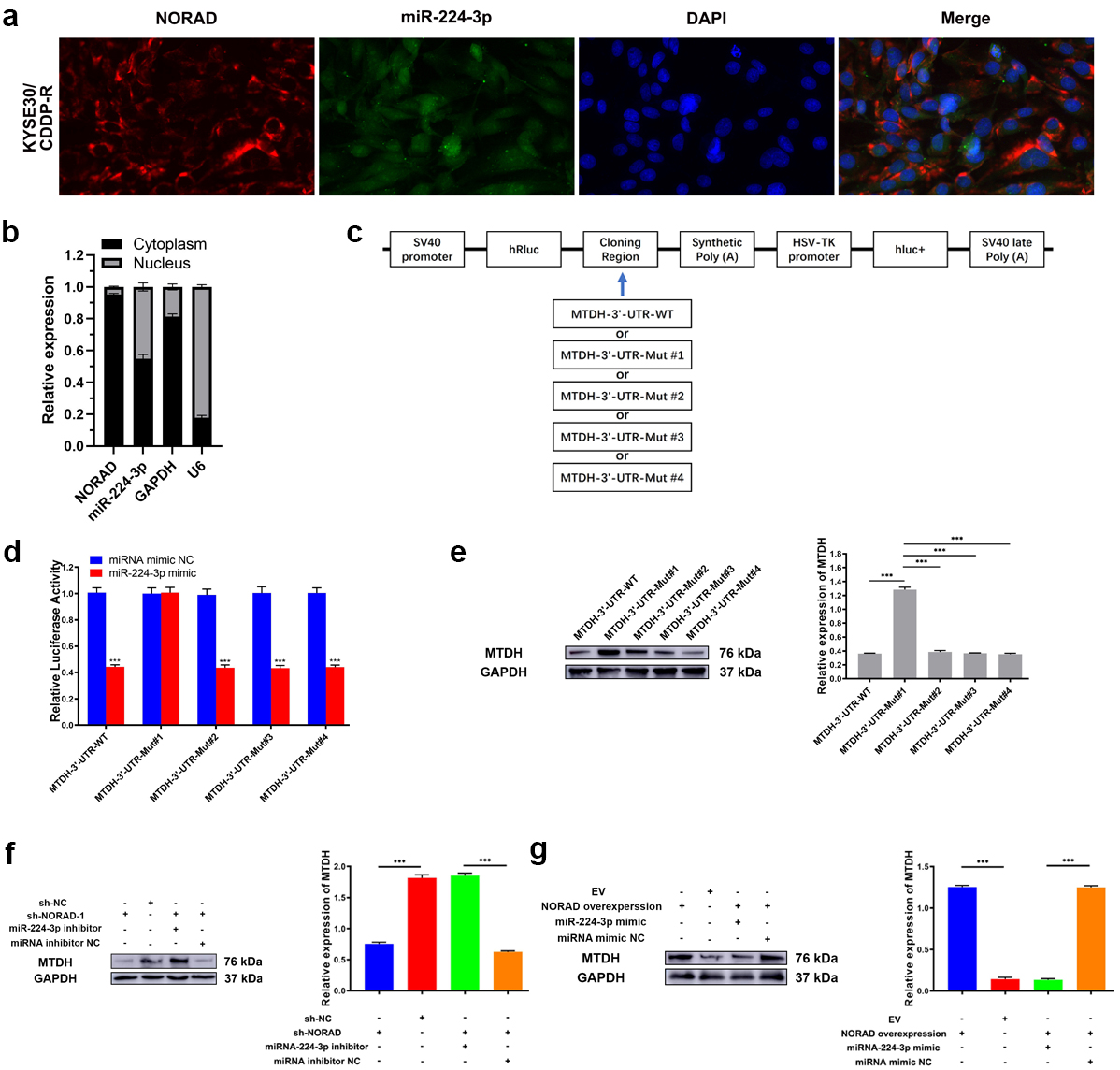

Supplement: Supplementary file 2 — Additional file 2: Figure S1. The effect of CDDP on cell viability of ESCC cells. a The effect of CDDP on cell viability of KYSE30/CDDP-R and KYSE30 cells. b The effect of CDDP on cell viability of TE1/CDDP-R and TE1 cells. Figure S2. Representative images of RNA FISH of NORAD in TE1/CDDP-R and TE1 cells (× 1000), which show that NORAD is predominantly located in the cytoplasm. Nuclei are stained with DAPI. Figure S3. The effect of sh-NORAD and overexpression vector on the expression of NORAD and proliferation ability of ESCC cells. a The effect of sh-NORAD on NORAD expression in KYSE30/CDDP-R and TE1/CDDP-R cells. Error bars denote SD of triplicates. ***P < 0.001. b The effect of overexpression of NORAD on NORAD expression in KYSE30 and TE1 cells. Error bars denote SD of triplicates. ***P < 0.001. c The effect of sh-NORAD on proliferation ability of KYSE30/CDDP-R and TE1/CDDP-R cells. d The effect of overexpression of NORAD on proliferation ability of KYSE30 and TE1 cells. The results are presented as the mean ± SD. ***P < 0.001. Figure S4. NORAD contributes to CDDP resistance of TE1 cells. a NORAD knockdown increases the sensitivity of TE1/CDDP-R cells to CDDP, detected by CCK-8. b Overexpression of NORAD decreases the sensitivity of TE1/CDDP-R cells to CDDP, detected by CCK-8. c NORAD knockdown decreases the colony formation ability of TE1/CDDP-R cells in the presence of CDDP. d Overexpression of NORAD increases the colony formation ability of TE1 cells in the presence of CDDP. e NORAD knockdown increases the CDDP-induced apoptosis rate of TE1/CDDP-R cells, and overexpression of NORAD decreases the CDDP-induced apoptosis rate of TE1 cells, detected by FCM. f NORAD knockdown facilitates CDDP to induce cell cycle arrest in TE1/CDDP-R cells. g Overexpression of NORAD suppresses CDDP-induced cell cycle arrest in TE1 cells. h NORAD knockdown increases the CDDP-induced γH2AX and cleaved caspase-3 in TE1/CDDP-R cells. i Overexpression of NORAD decreases the CDDP-induced [file 12943_2021_1455_MOESM2_ESM.zip › Figure S5.jpg]

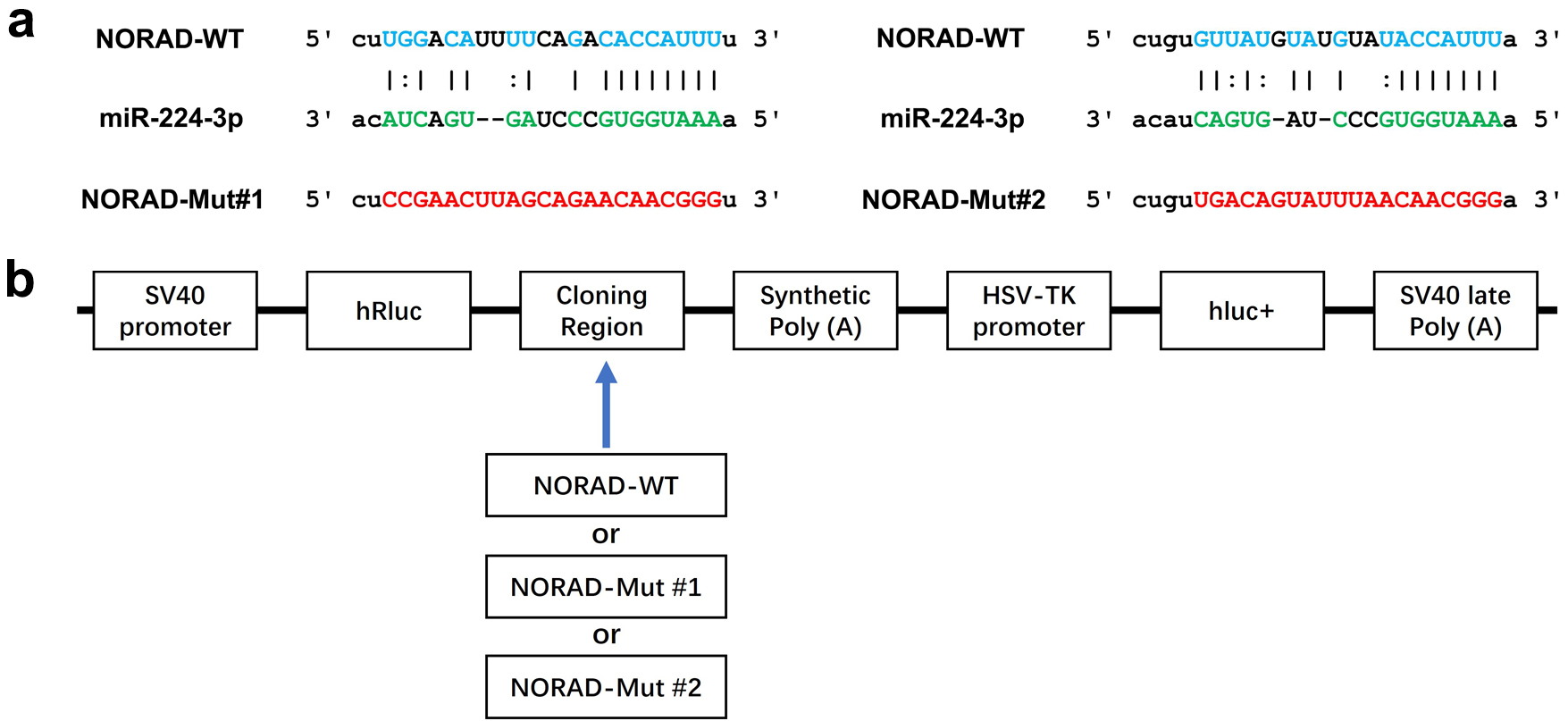

Supplement: Supplementary file 2 — Additional file 2: Figure S1. The effect of CDDP on cell viability of ESCC cells. a The effect of CDDP on cell viability of KYSE30/CDDP-R and KYSE30 cells. b The effect of CDDP on cell viability of TE1/CDDP-R and TE1 cells. Figure S2. Representative images of RNA FISH of NORAD in TE1/CDDP-R and TE1 cells (× 1000), which show that NORAD is predominantly located in the cytoplasm. Nuclei are stained with DAPI. Figure S3. The effect of sh-NORAD and overexpression vector on the expression of NORAD and proliferation ability of ESCC cells. a The effect of sh-NORAD on NORAD expression in KYSE30/CDDP-R and TE1/CDDP-R cells. Error bars denote SD of triplicates. ***P < 0.001. b The effect of overexpression of NORAD on NORAD expression in KYSE30 and TE1 cells. Error bars denote SD of triplicates. ***P < 0.001. c The effect of sh-NORAD on proliferation ability of KYSE30/CDDP-R and TE1/CDDP-R cells. d The effect of overexpression of NORAD on proliferation ability of KYSE30 and TE1 cells. The results are presented as the mean ± SD. ***P < 0.001. Figure S4. NORAD contributes to CDDP resistance of TE1 cells. a NORAD knockdown increases the sensitivity of TE1/CDDP-R cells to CDDP, detected by CCK-8. b Overexpression of NORAD decreases the sensitivity of TE1/CDDP-R cells to CDDP, detected by CCK-8. c NORAD knockdown decreases the colony formation ability of TE1/CDDP-R cells in the presence of CDDP. d Overexpression of NORAD increases the colony formation ability of TE1 cells in the presence of CDDP. e NORAD knockdown increases the CDDP-induced apoptosis rate of TE1/CDDP-R cells, and overexpression of NORAD decreases the CDDP-induced apoptosis rate of TE1 cells, detected by FCM. f NORAD knockdown facilitates CDDP to induce cell cycle arrest in TE1/CDDP-R cells. g Overexpression of NORAD suppresses CDDP-induced cell cycle arrest in TE1 cells. h NORAD knockdown increases the CDDP-induced γH2AX and cleaved caspase-3 in TE1/CDDP-R cells. i Overexpression of NORAD decreases the CDDP-induced [file 12943_2021_1455_MOESM2_ESM.zip › Figure S6.jpg]

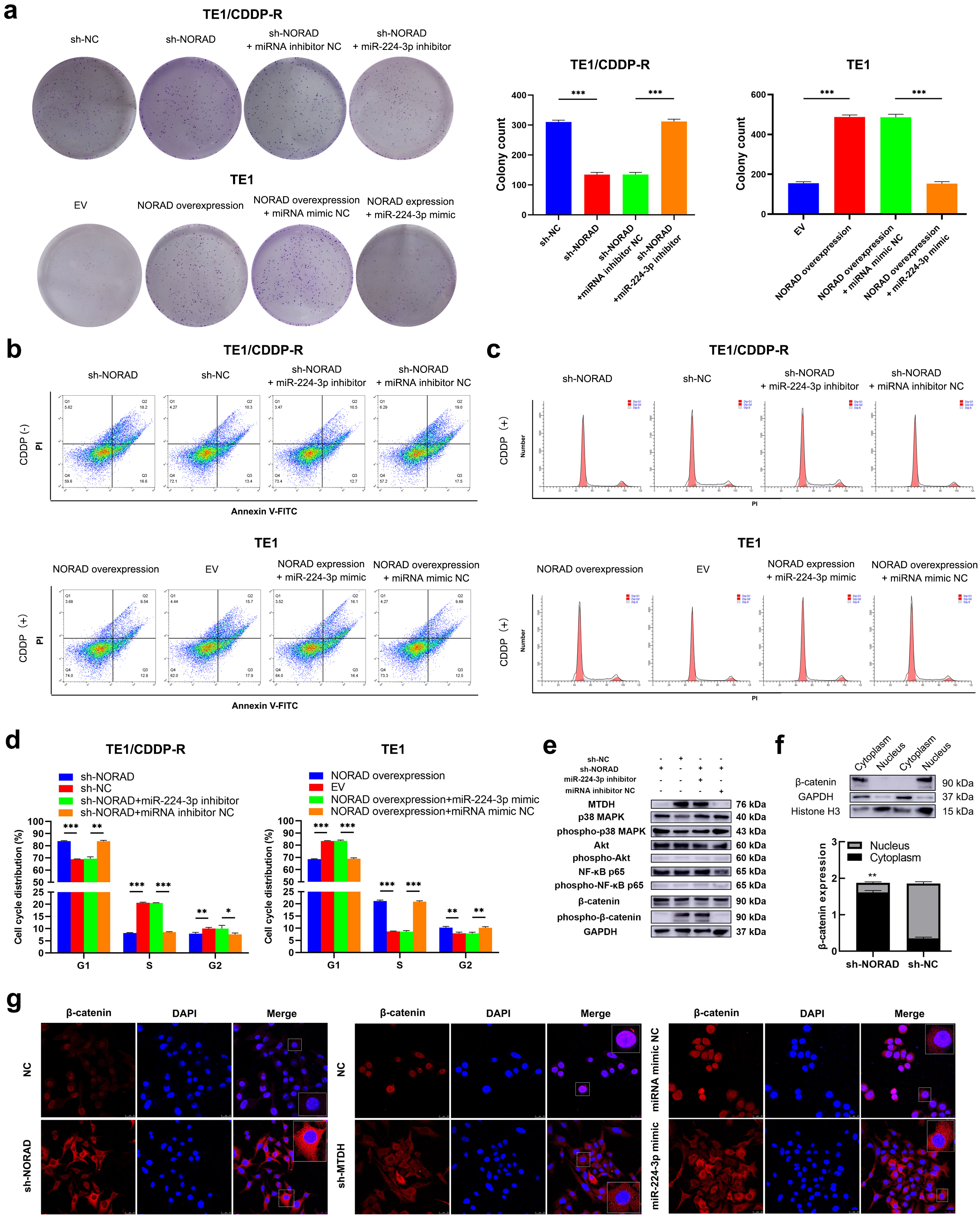

Supplement: Supplementary file 2 — Additional file 2: Figure S1. The effect of CDDP on cell viability of ESCC cells. a The effect of CDDP on cell viability of KYSE30/CDDP-R and KYSE30 cells. b The effect of CDDP on cell viability of TE1/CDDP-R and TE1 cells. Figure S2. Representative images of RNA FISH of NORAD in TE1/CDDP-R and TE1 cells (× 1000), which show that NORAD is predominantly located in the cytoplasm. Nuclei are stained with DAPI. Figure S3. The effect of sh-NORAD and overexpression vector on the expression of NORAD and proliferation ability of ESCC cells. a The effect of sh-NORAD on NORAD expression in KYSE30/CDDP-R and TE1/CDDP-R cells. Error bars denote SD of triplicates. ***P < 0.001. b The effect of overexpression of NORAD on NORAD expression in KYSE30 and TE1 cells. Error bars denote SD of triplicates. ***P < 0.001. c The effect of sh-NORAD on proliferation ability of KYSE30/CDDP-R and TE1/CDDP-R cells. d The effect of overexpression of NORAD on proliferation ability of KYSE30 and TE1 cells. The results are presented as the mean ± SD. ***P < 0.001. Figure S4. NORAD contributes to CDDP resistance of TE1 cells. a NORAD knockdown increases the sensitivity of TE1/CDDP-R cells to CDDP, detected by CCK-8. b Overexpression of NORAD decreases the sensitivity of TE1/CDDP-R cells to CDDP, detected by CCK-8. c NORAD knockdown decreases the colony formation ability of TE1/CDDP-R cells in the presence of CDDP. d Overexpression of NORAD increases the colony formation ability of TE1 cells in the presence of CDDP. e NORAD knockdown increases the CDDP-induced apoptosis rate of TE1/CDDP-R cells, and overexpression of NORAD decreases the CDDP-induced apoptosis rate of TE1 cells, detected by FCM. f NORAD knockdown facilitates CDDP to induce cell cycle arrest in TE1/CDDP-R cells. g Overexpression of NORAD suppresses CDDP-induced cell cycle arrest in TE1 cells. h NORAD knockdown increases the CDDP-induced γH2AX and cleaved caspase-3 in TE1/CDDP-R cells. i Overexpression of NORAD decreases the CDDP-induced [file 12943_2021_1455_MOESM2_ESM.zip › Figure S7.jpg]

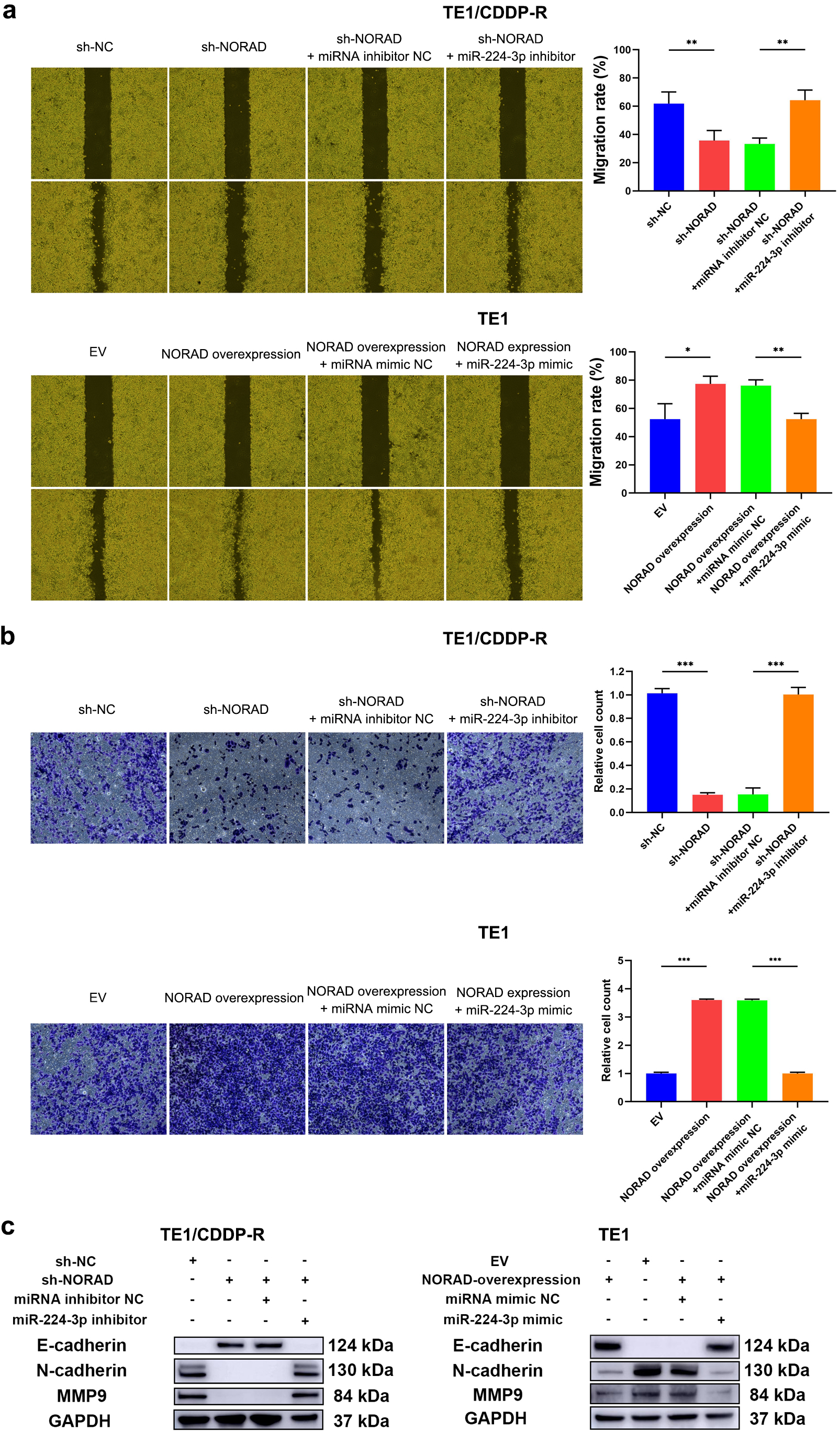

Supplement: Supplementary file 2 — Additional file 2: Figure S1. The effect of CDDP on cell viability of ESCC cells. a The effect of CDDP on cell viability of KYSE30/CDDP-R and KYSE30 cells. b The effect of CDDP on cell viability of TE1/CDDP-R and TE1 cells. Figure S2. Representative images of RNA FISH of NORAD in TE1/CDDP-R and TE1 cells (× 1000), which show that NORAD is predominantly located in the cytoplasm. Nuclei are stained with DAPI. Figure S3. The effect of sh-NORAD and overexpression vector on the expression of NORAD and proliferation ability of ESCC cells. a The effect of sh-NORAD on NORAD expression in KYSE30/CDDP-R and TE1/CDDP-R cells. Error bars denote SD of triplicates. ***P < 0.001. b The effect of overexpression of NORAD on NORAD expression in KYSE30 and TE1 cells. Error bars denote SD of triplicates. ***P < 0.001. c The effect of sh-NORAD on proliferation ability of KYSE30/CDDP-R and TE1/CDDP-R cells. d The effect of overexpression of NORAD on proliferation ability of KYSE30 and TE1 cells. The results are presented as the mean ± SD. ***P < 0.001. Figure S4. NORAD contributes to CDDP resistance of TE1 cells. a NORAD knockdown increases the sensitivity of TE1/CDDP-R cells to CDDP, detected by CCK-8. b Overexpression of NORAD decreases the sensitivity of TE1/CDDP-R cells to CDDP, detected by CCK-8. c NORAD knockdown decreases the colony formation ability of TE1/CDDP-R cells in the presence of CDDP. d Overexpression of NORAD increases the colony formation ability of TE1 cells in the presence of CDDP. e NORAD knockdown increases the CDDP-induced apoptosis rate of TE1/CDDP-R cells, and overexpression of NORAD decreases the CDDP-induced apoptosis rate of TE1 cells, detected by FCM. f NORAD knockdown facilitates CDDP to induce cell cycle arrest in TE1/CDDP-R cells. g Overexpression of NORAD suppresses CDDP-induced cell cycle arrest in TE1 cells. h NORAD knockdown increases the CDDP-induced γH2AX and cleaved caspase-3 in TE1/CDDP-R cells. i Overexpression of NORAD decreases the CDDP-induced [file 12943_2021_1455_MOESM2_ESM.zip › Figure S8.jpg]
