# Supplementary material for: Long non-coding RNA NORAD/miR-224-3p/MTDH axis contributes to CDDP resistance of esophageal squamous cell carcinoma by promoting nuclear accumulation of β-catenin
Source: Mol Cancer. 2021 Dec 10;20:162. doi: 10.1186/s12943-021-01455-y (PMC8662861; doi:10.1186/s12943-021-01455-y)
Supplement: Supplementary file 3 — Additional file 3. [file 12943_2021_1455_MOESM3_ESM.docx]

**Establishment of CDDP-Resistant Cell Lines**

The CDDP-resistant strains of KYSE30 and TE1, referred as KYSE30/CDDP-R and TE1/CDDP-R, were established by exposure to gradually increasing concentrations of CDDP (from 0.2 to 10 µM) over a period of 6 months. In detail, KYSE30 or TE1 cells at the logarithmic phase were exposed to CDDP at an initial concentration of 0.2 µM in RPMI-1640 medium plus 10% FBS. After 48 h, the treated KYSE30 or TE1 cells were washed three times with PBS and cultured in CDDP-free RPMI-1640 medium plus 10% FBS. Upon reaching of 70 - 80% confluence, the ESCC cells were exposed to CDDP at higher concentration (10 - 20% increase per passage). The above treatment was repeated until it reached a concentration of 10 µM, the KYSE30 and TE1 cells which were resistant to CDDP were obtained.
