# Supplementary material for: Long non-coding RNA NORAD/miR-224-3p/MTDH axis contributes to CDDP resistance of esophageal squamous cell carcinoma by promoting nuclear accumulation of β-catenin
Source: Mol Cancer. 2021 Dec 10;20:162. doi: 10.1186/s12943-021-01455-y (PMC8662861; doi:10.1186/s12943-021-01455-y)

γH2AX of CDDP-treated group in Fig. 2j


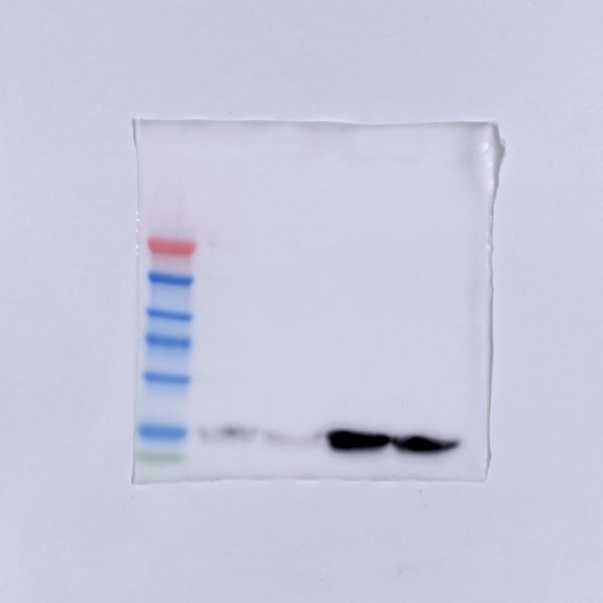


caspase-3 of CDDP-treated group in Fig. 2j


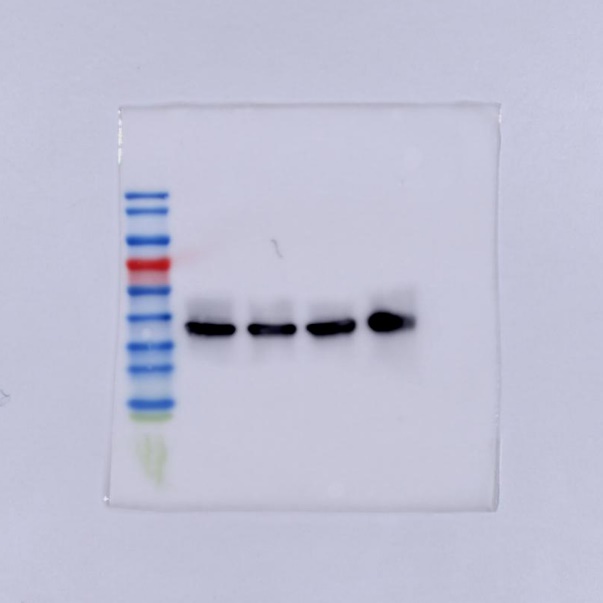


cleaved caspase-3 of CDDP-treated group in Fig. 2j


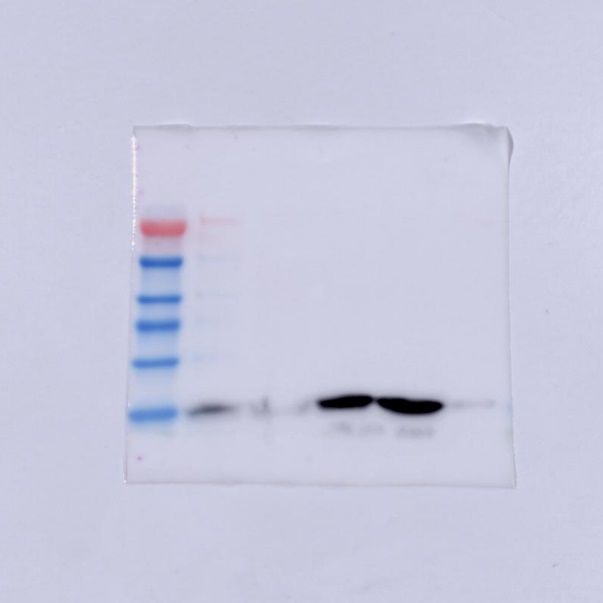


GAPDH of CDDP-treated group in Fig. 2j


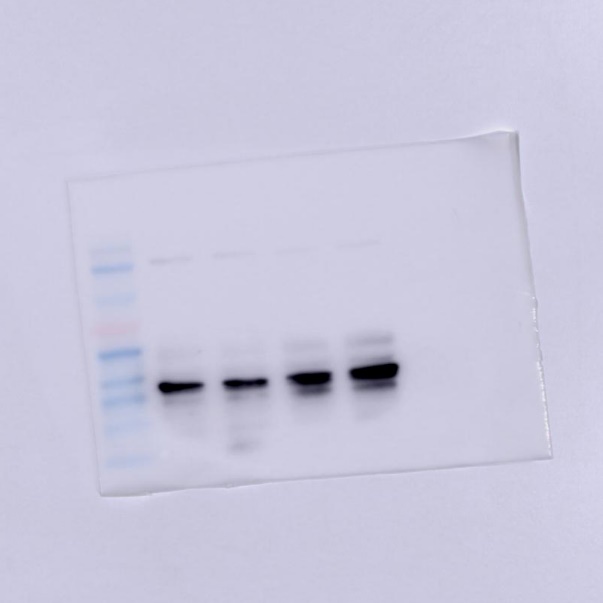


γH2AX of PBS-treated group in Fig. 2j


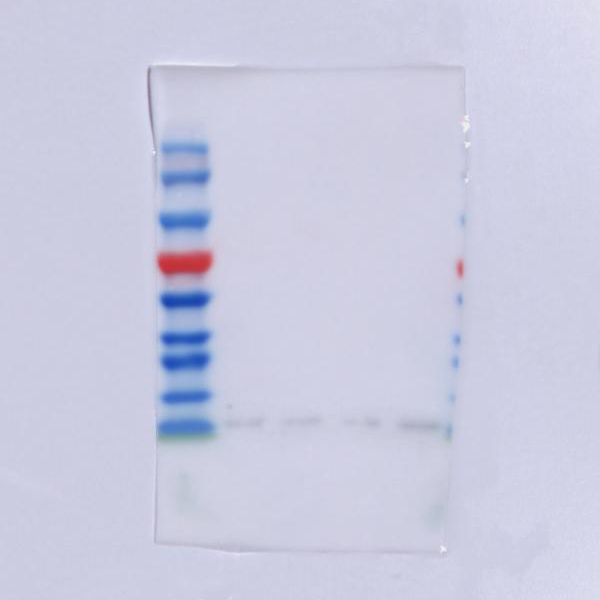


caspase-3 of PBS-treated group in Fig. 2j


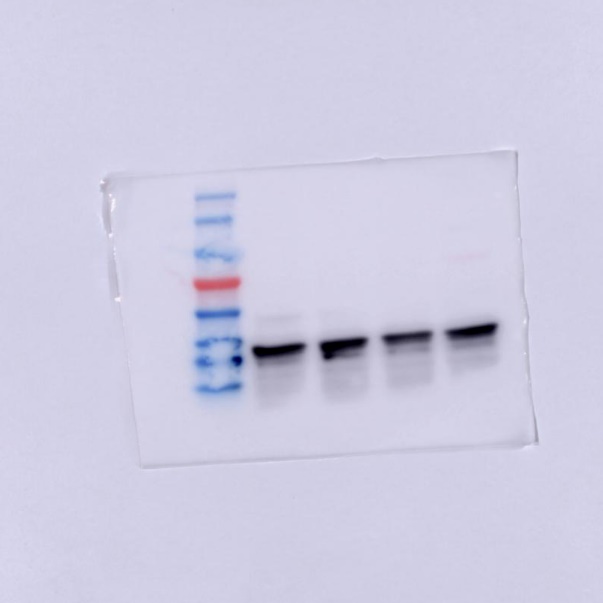


cleaved caspase-3 of PBS-treated group in Fig. 2j


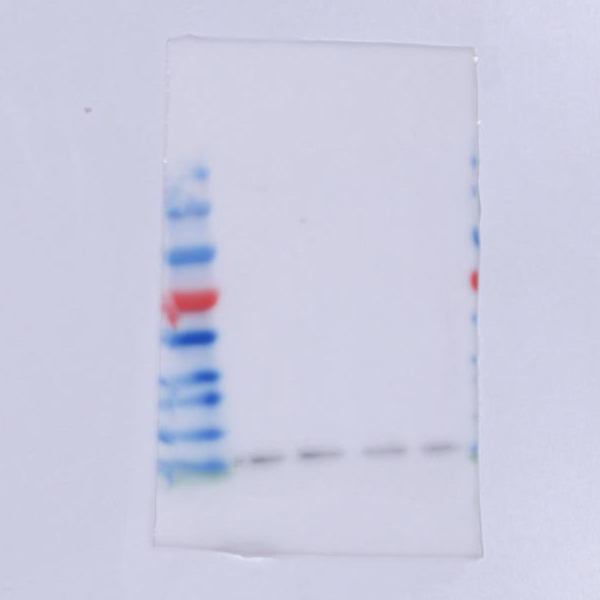


GAPDH of PBS-treated group in Fig. 2j


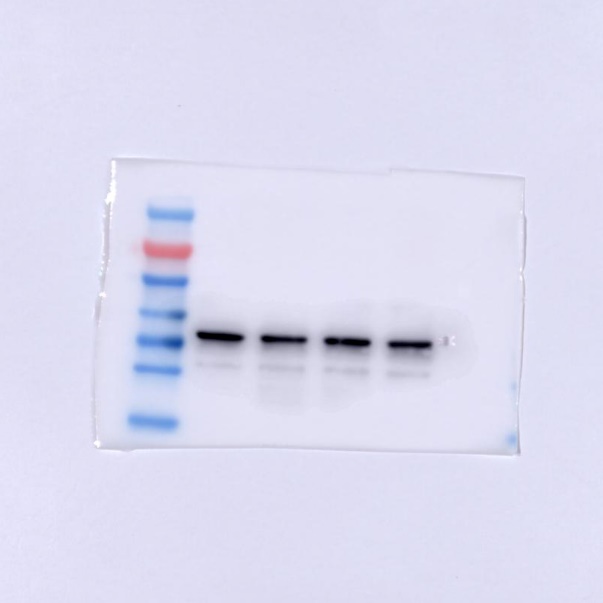


γH2AX of CDDP-treated group in Fig. 2k


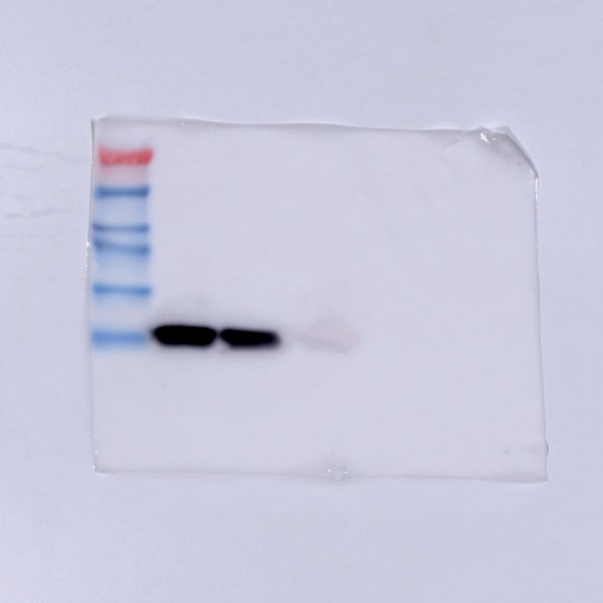


caspase-3 of CDDP-treated group in Fig. 2k (left 3 lanes) and Fig. S4i (right 3 lanes)


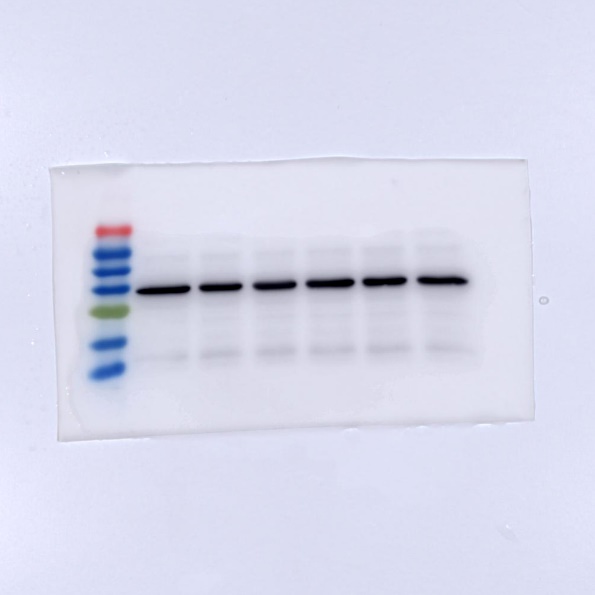


cleaved caspase-3 of CDDP-treated group in Fig. 2k


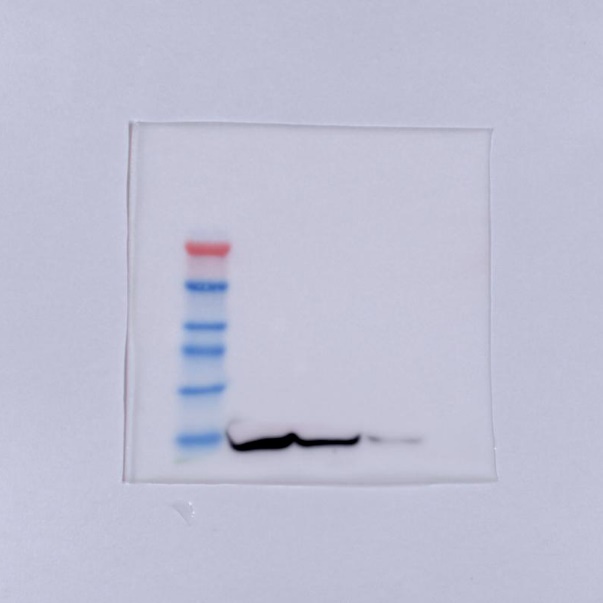


GAPDH of CDDP-treated group in Fig. 2k (left 3 lanes) and Fig. S4i (right 3 lanes)


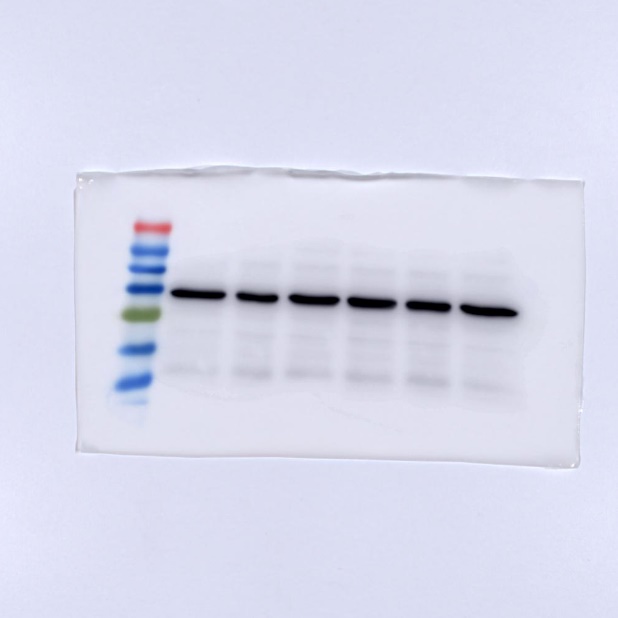


MTDH in Fig. 4d (left 2 lanes represent KYSE30/CDDP-R and KYSE30, right 2 lanes represent TE1/CDDP-R and TE1)


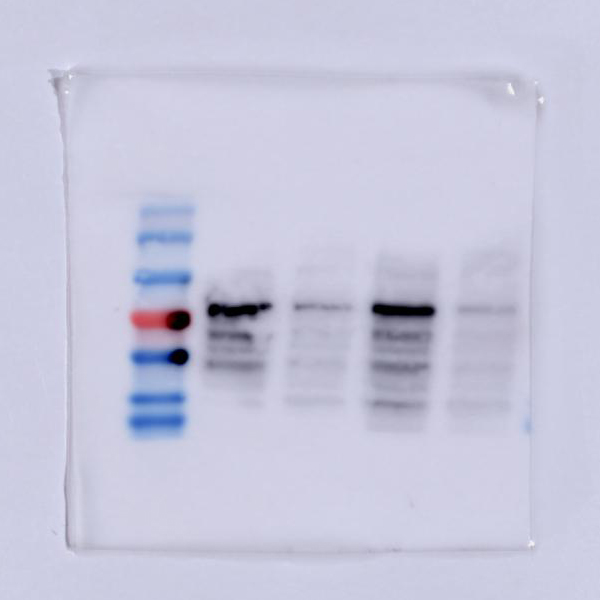


GAPDH of KYSE30/CDDP-R and KYSE30 in Fig. 4d


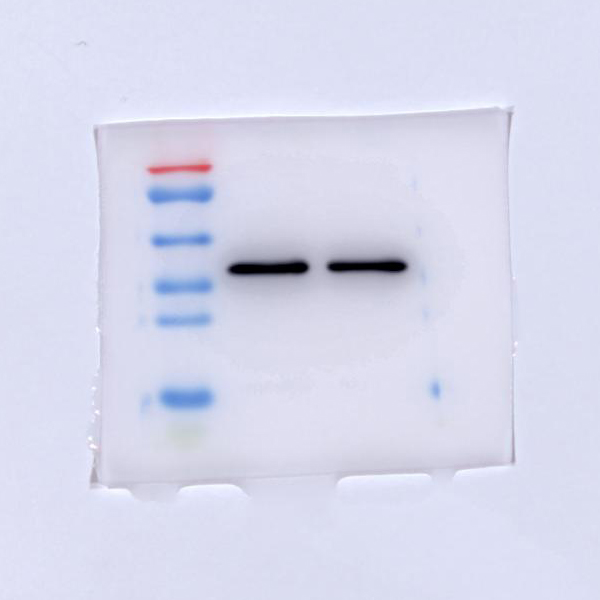


GAPDH of TE1/CDDP-R and TE1 in Fig. 4d


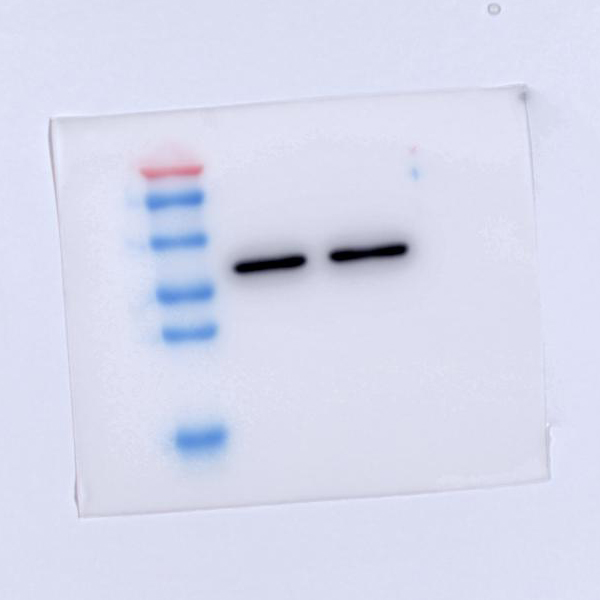


MTDH in Fig. 4g


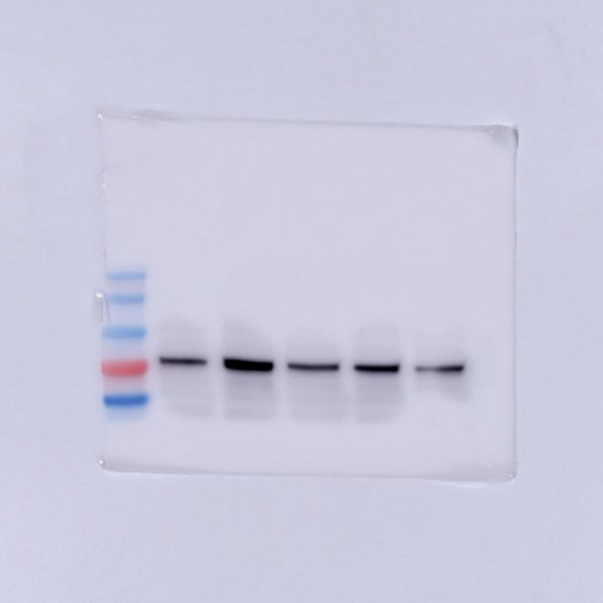


GAPDH in Fig. 4g


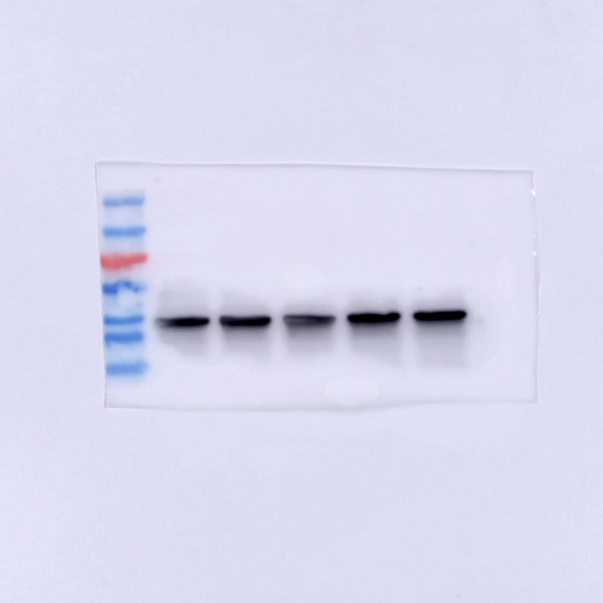


MTDH in Fig. 4h


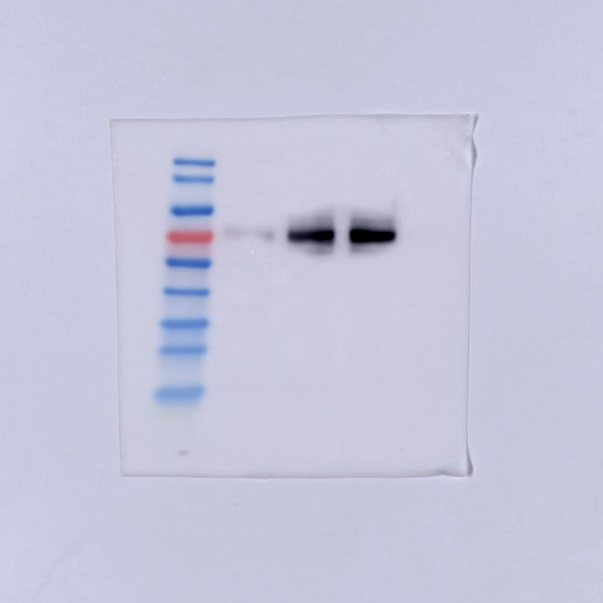


GAPDH in Fig. 4h and Fig. 5e


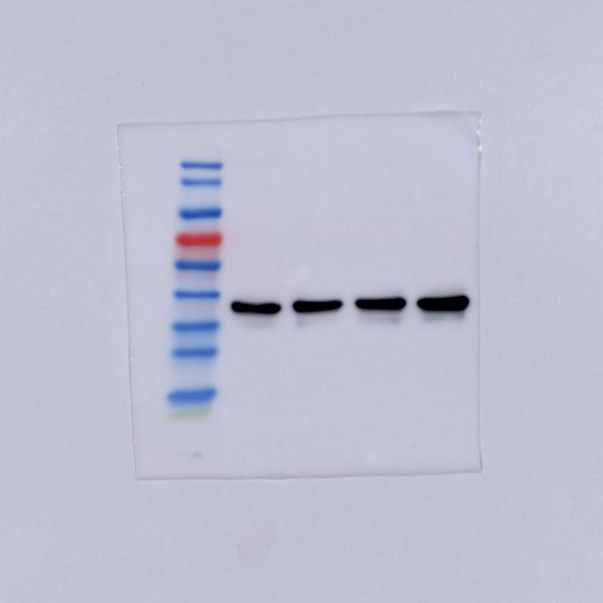


MTDH in Fig. 4i


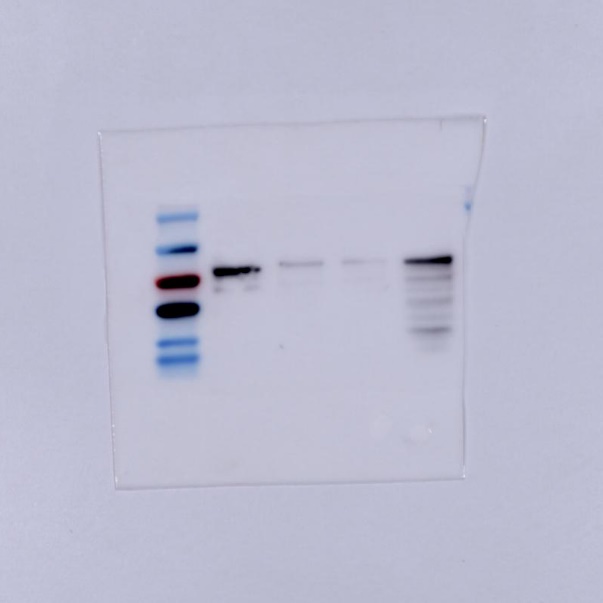


GAPDH in Fig. 4i


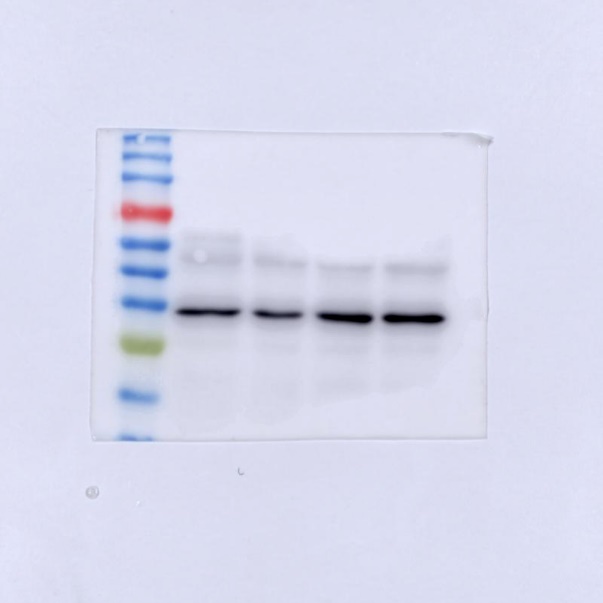


MTDH in Fig. 5e


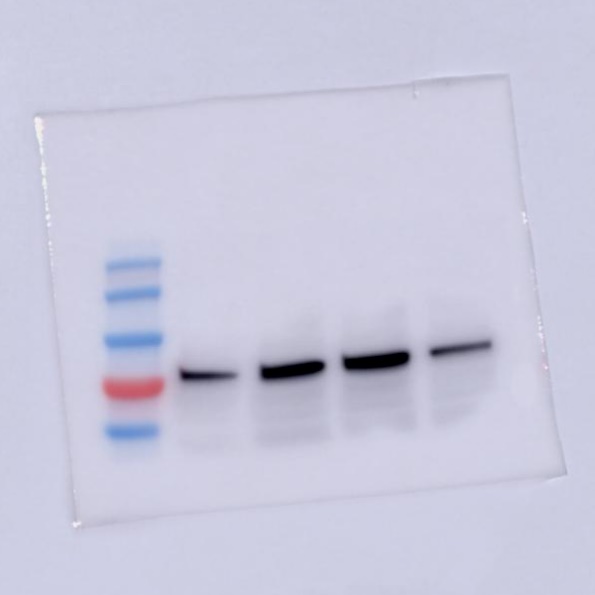


p38 MAPK in Fig. 5e


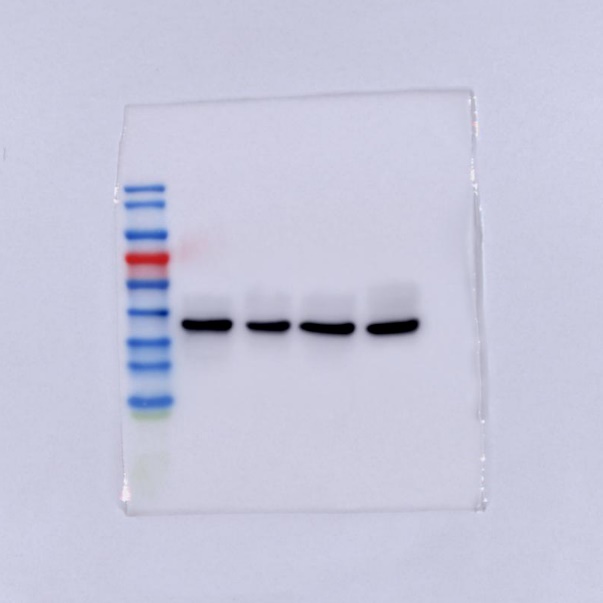


phospho-p38 MAPK in Fig. 5e


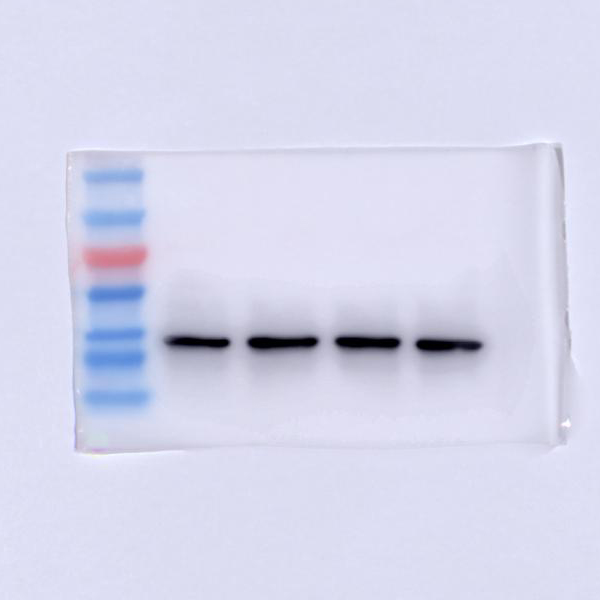


AKT in Fig. 5e


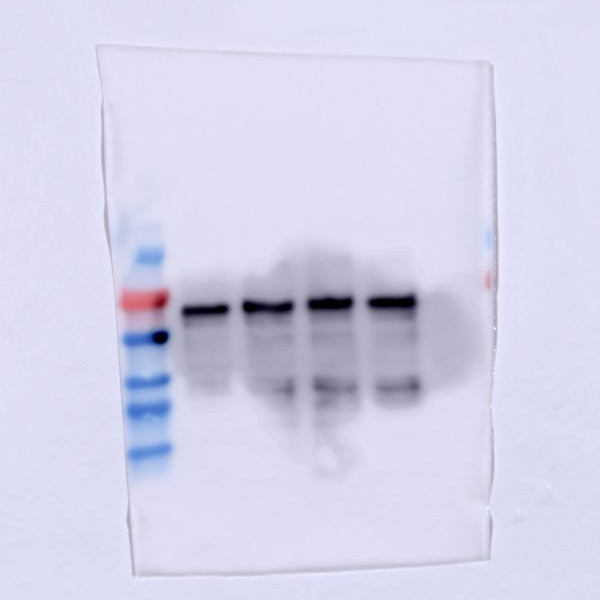


phospho-AKT in Fig. 5e


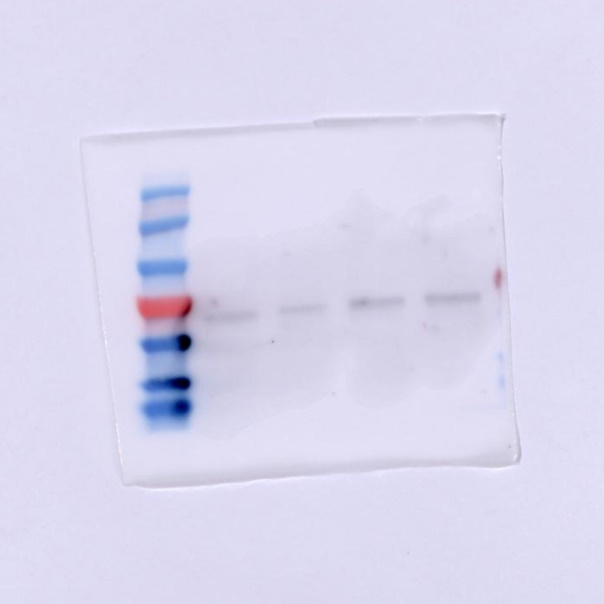


NF-κB in Fig. 5e


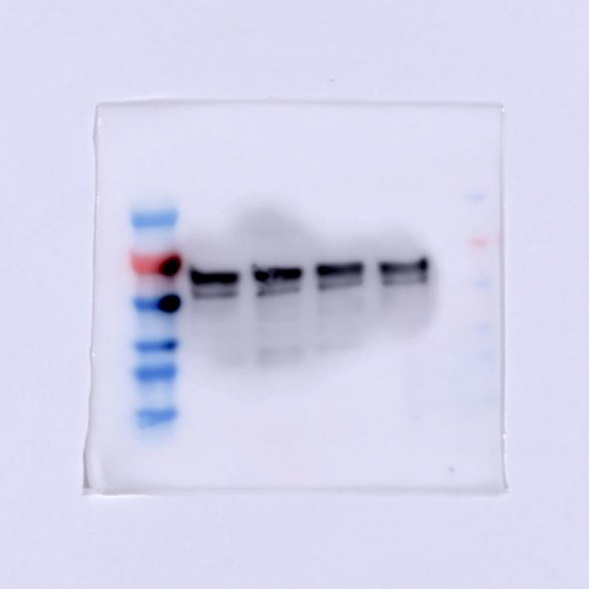


phospho- NF-κB in Fig. 5e


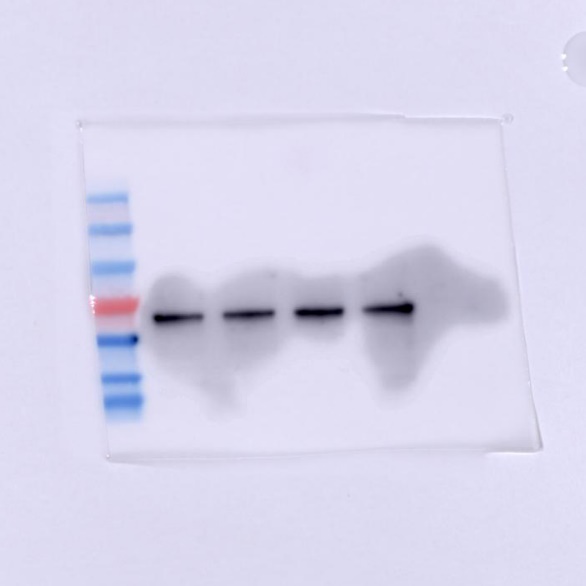


β-catenin in Fig. 5e


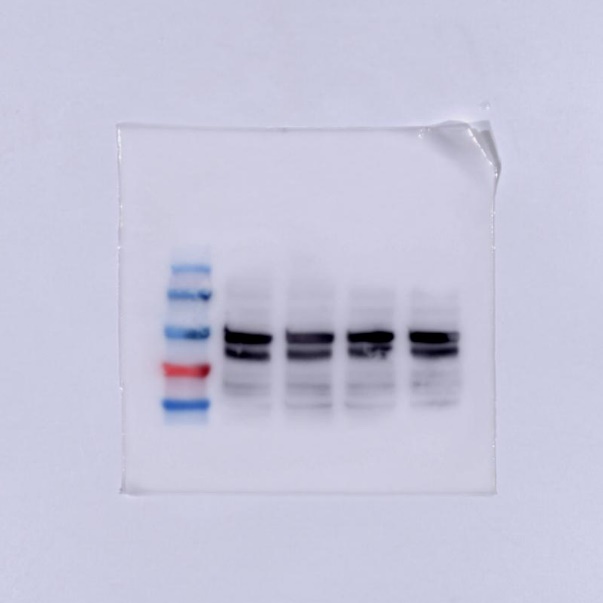


phospho-β-catenin in Fig. 5e


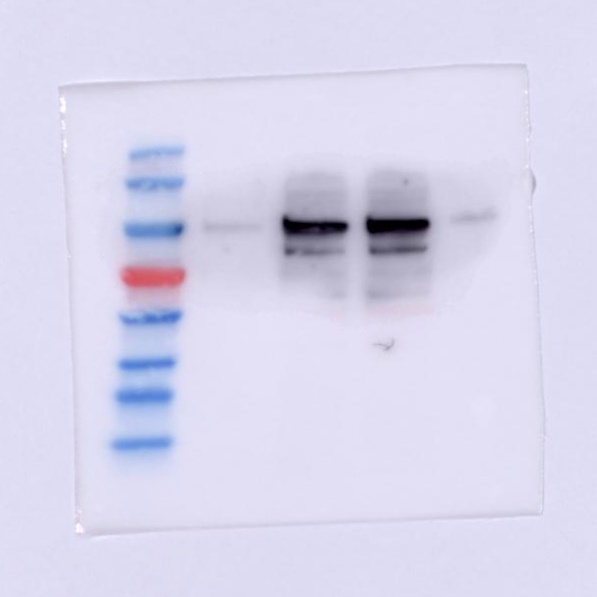


GAPDH in Fig. 5e


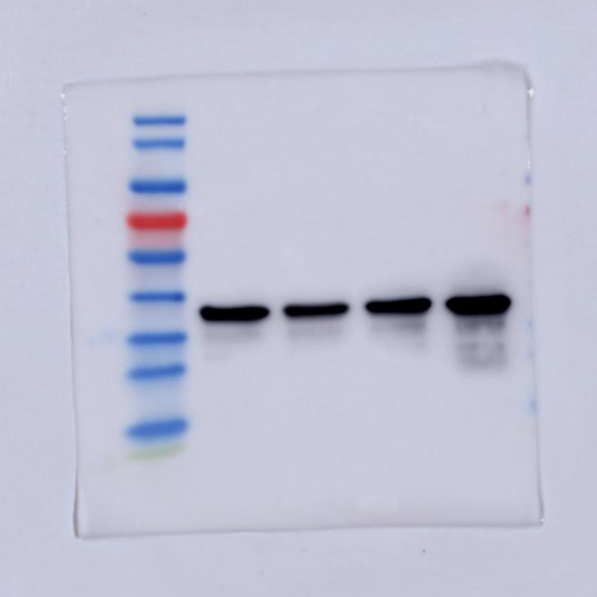


β-catenin in Fig. 5f


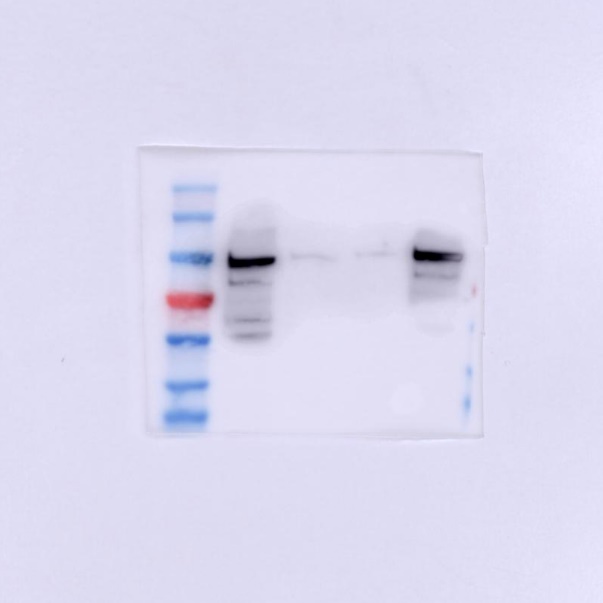


GAPDH in Fig. 5f


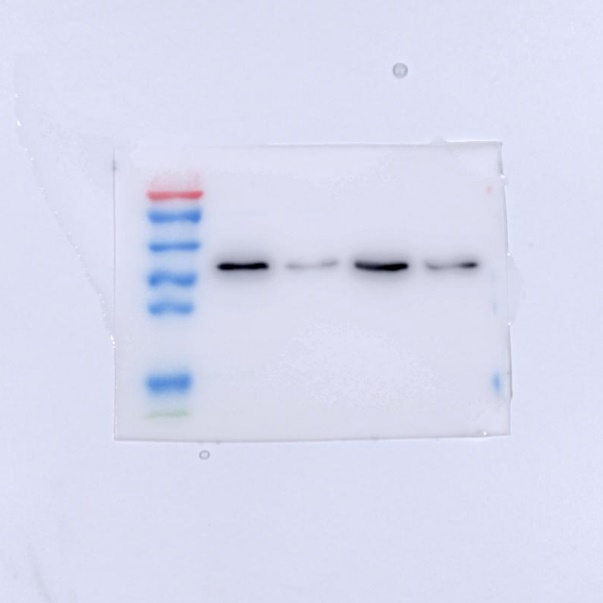


Histone H3 in Fig. 5f


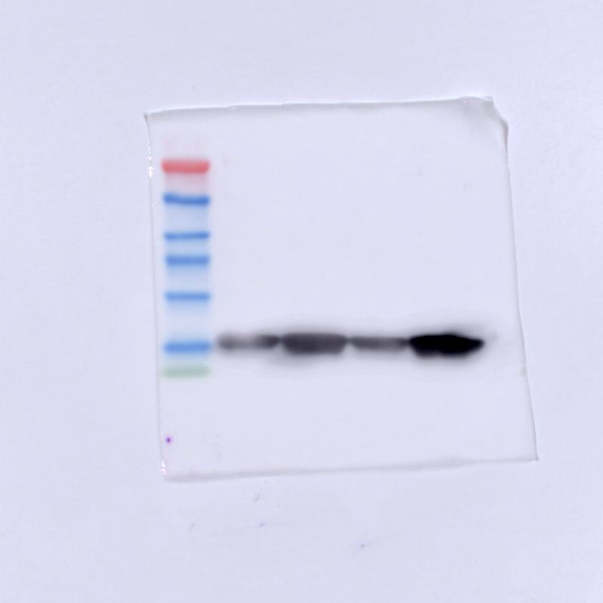


E-cadherin in KYSE30/CDDP-R in Fig. 6c


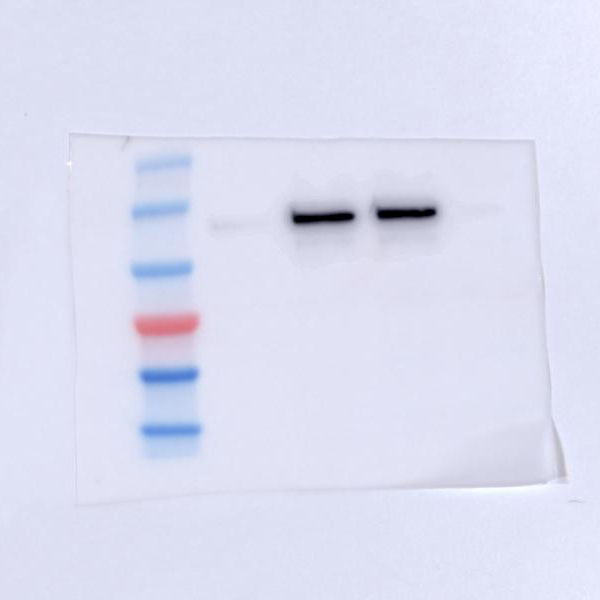


N-cadherin in KYSE30/CDDP-R in Fig. 6c


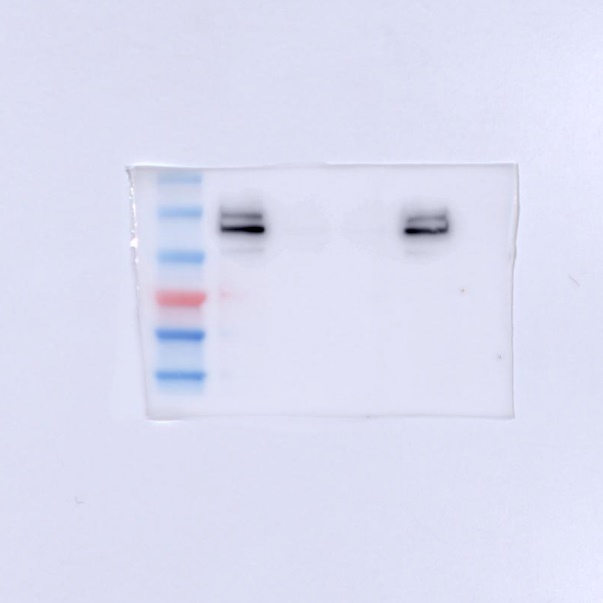


MMP9 in KYSE30/CDDP-R in Fig. 6c


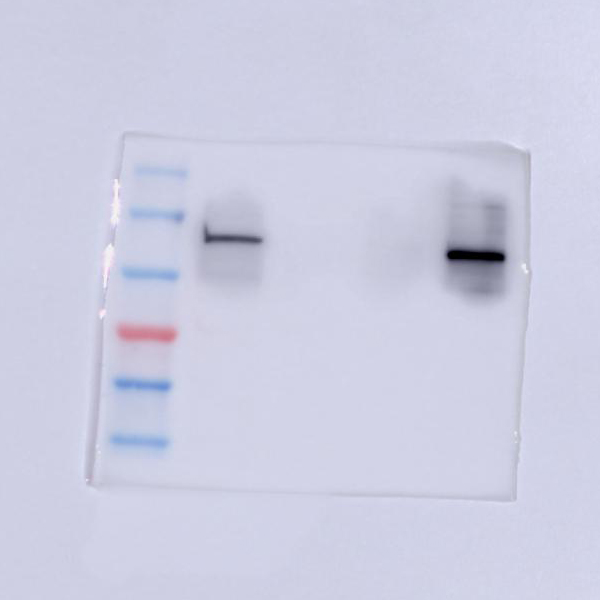


GAPDH in KYSE30/CDDP-R in Fig. 6c


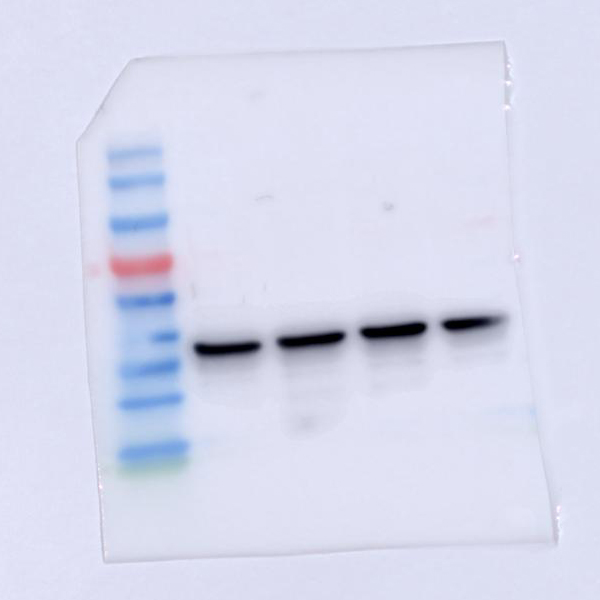


E-cadherin in KYSE30 in Fig. 6c


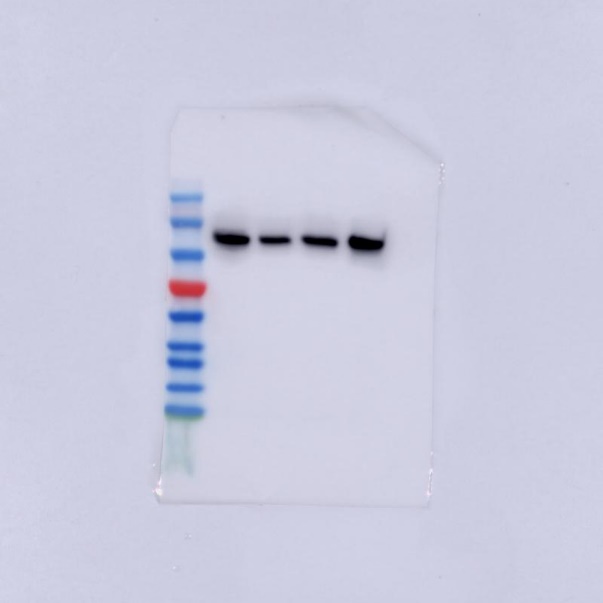


N-cadherin in KYSE30 in Fig. 6c


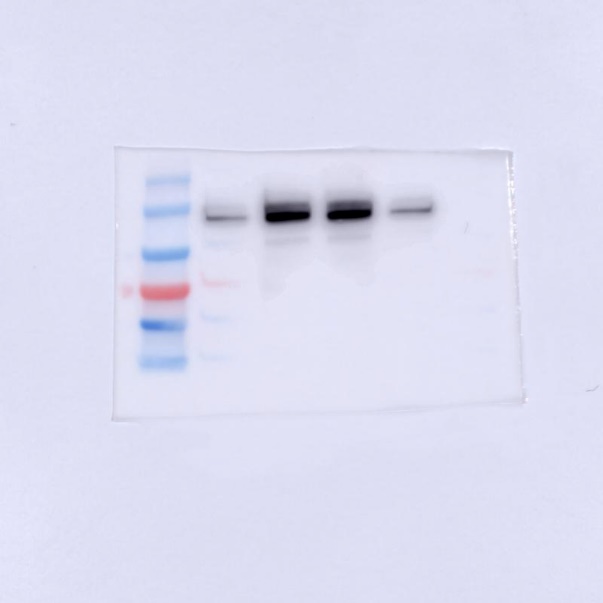


MMP9 in KYSE30 in Fig. 6c


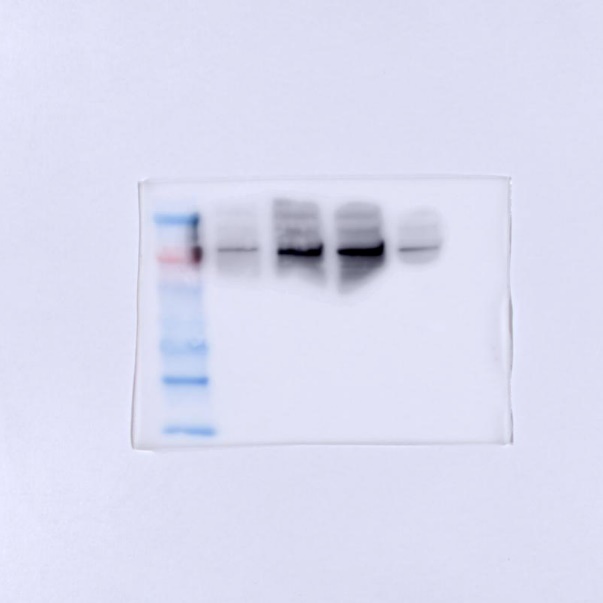


GAPDH in KYSE30 in Fig. 6c


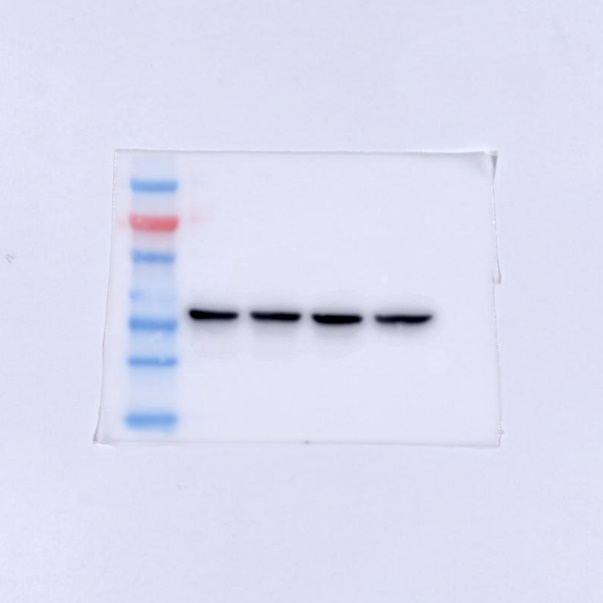


γH2AX of CDDP-treated group in Fig. S4h


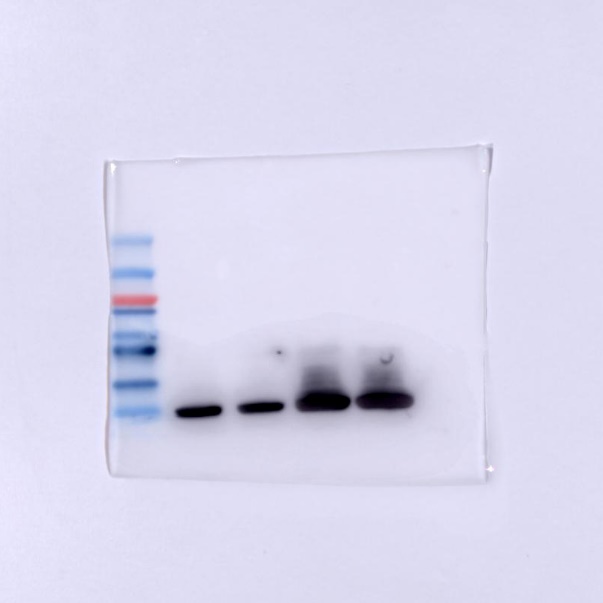


caspase-3 of CDDP-treated group in Fig. S4h


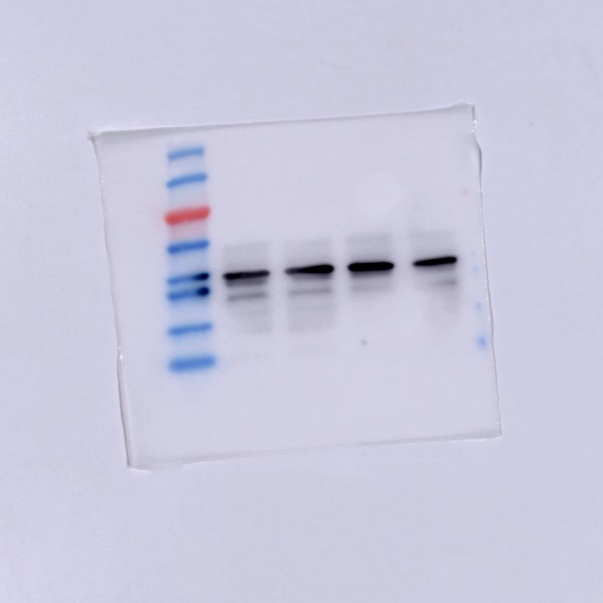


cleaved caspase-3 of CDDP-treated group in Fig. S4h


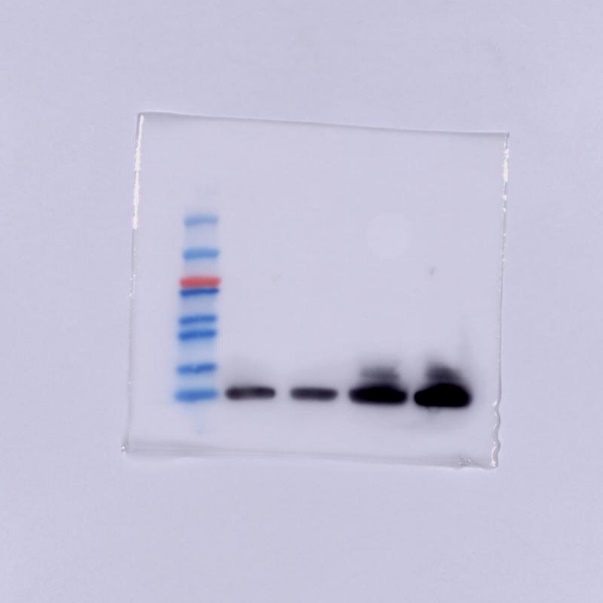


GAPDH of CDDP-treated group in Fig. S4h


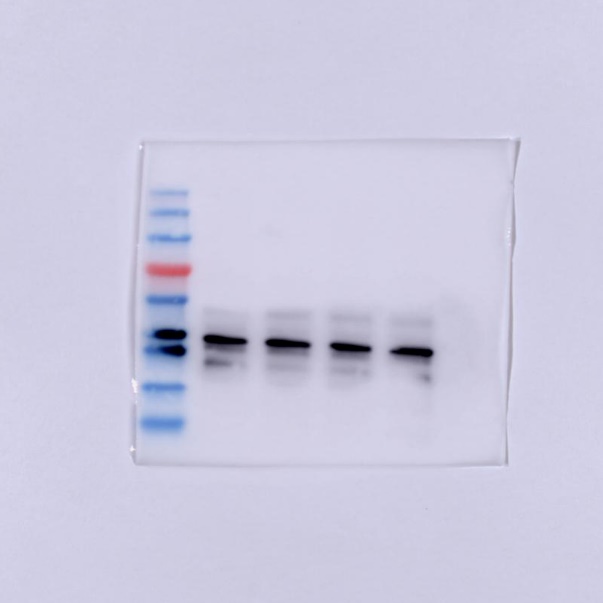


γH2AX of PBS-treated group in Fig. S4h


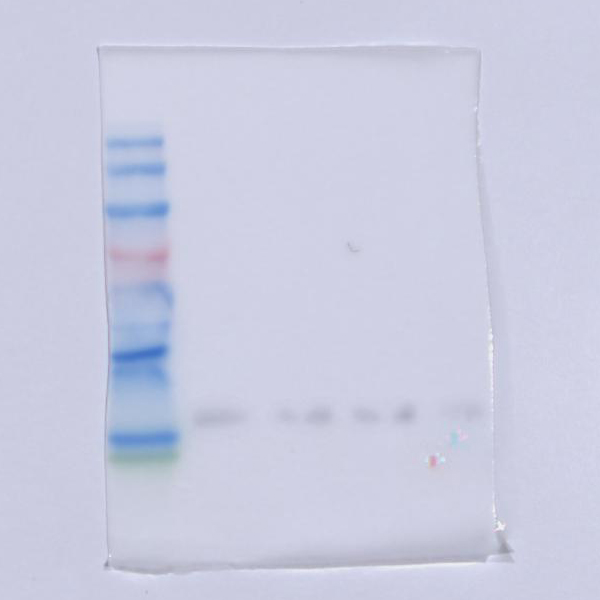


caspase-3 of PBS-treated group in Fig. S4h


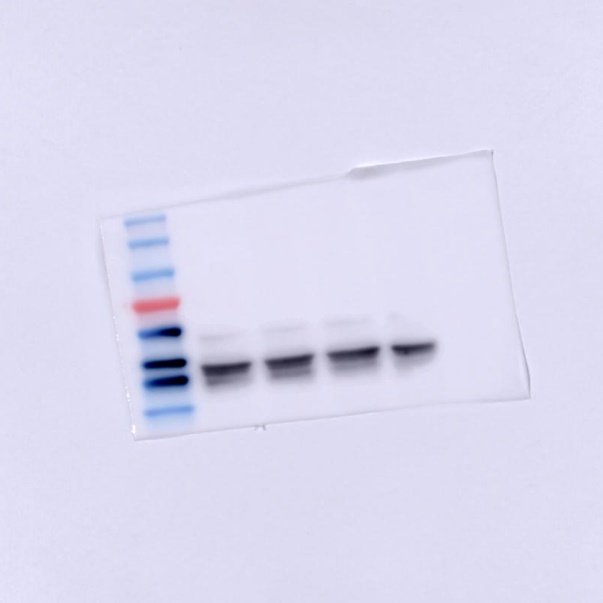


cleaved caspase-3 of PBS-treated group in Fig. S4h


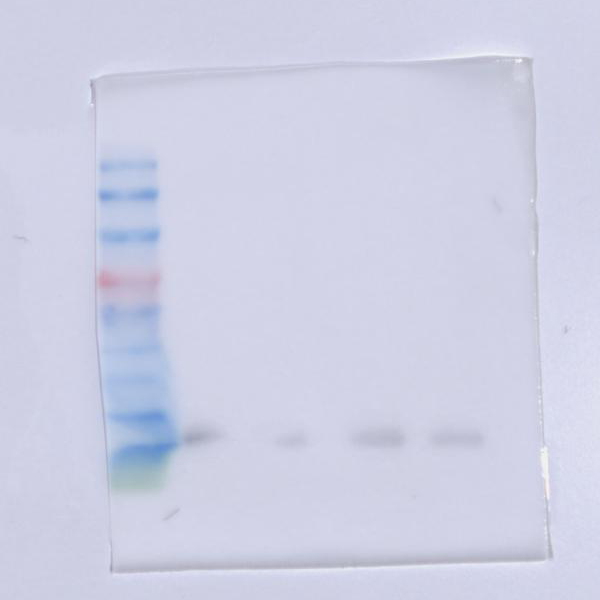


GAPDH of PBS-treated group in Fig. S4h


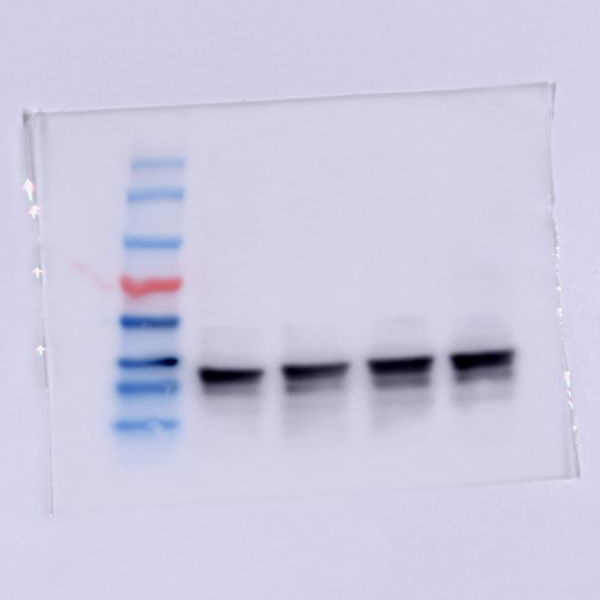


γH2AX of CDDP-treated group in Fig. S4i


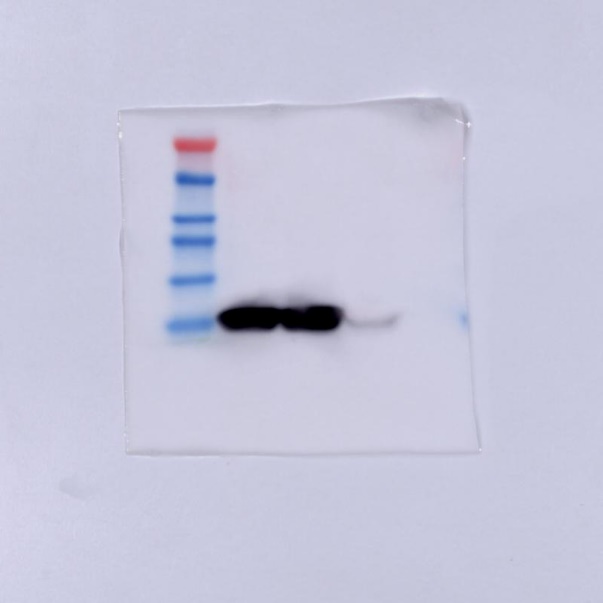


cleaved caspase-3 of CDDP-treated group in Fig. S4i


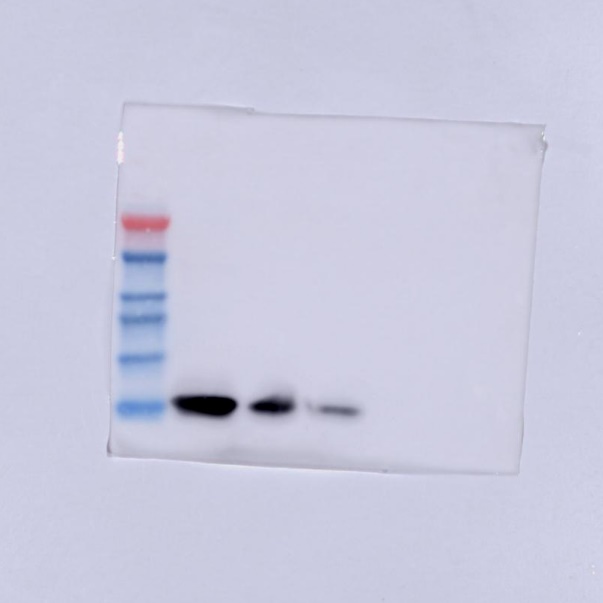


MTDH in Fig. S5e


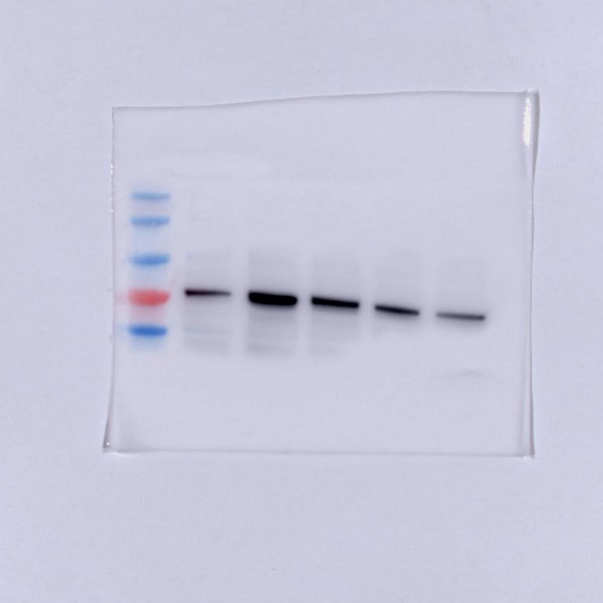


GAPDH in Fig. S5e


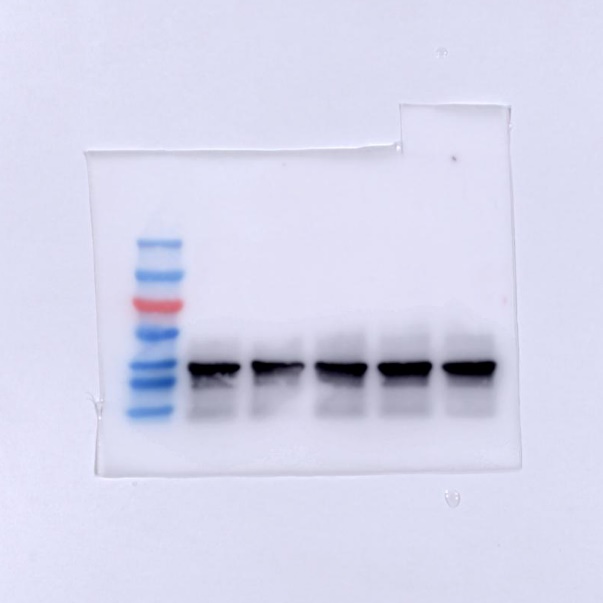


MTDH in Fig. S5f


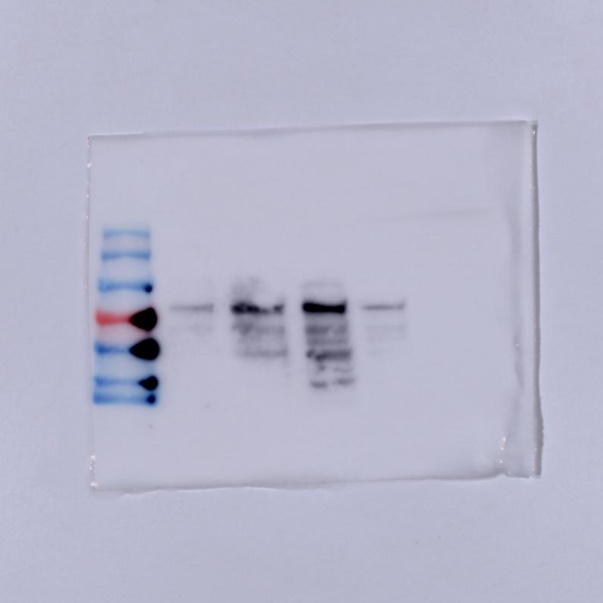


GAPDH in Fig. S5f


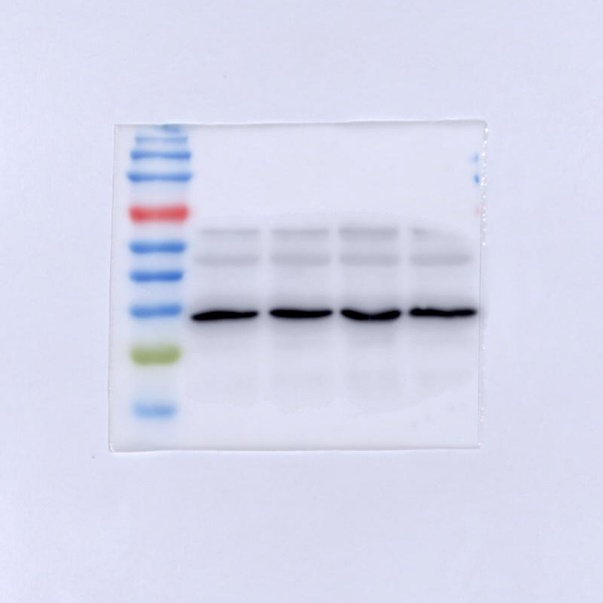


MTDH in Fig. S5g


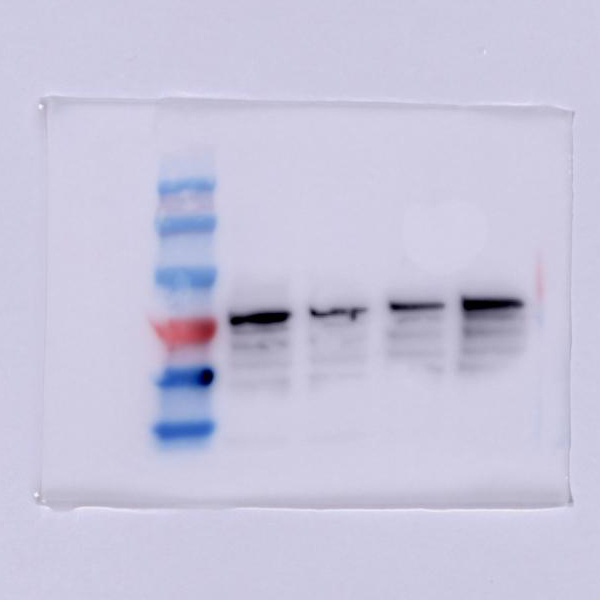


GAPDH in Fig. S5g


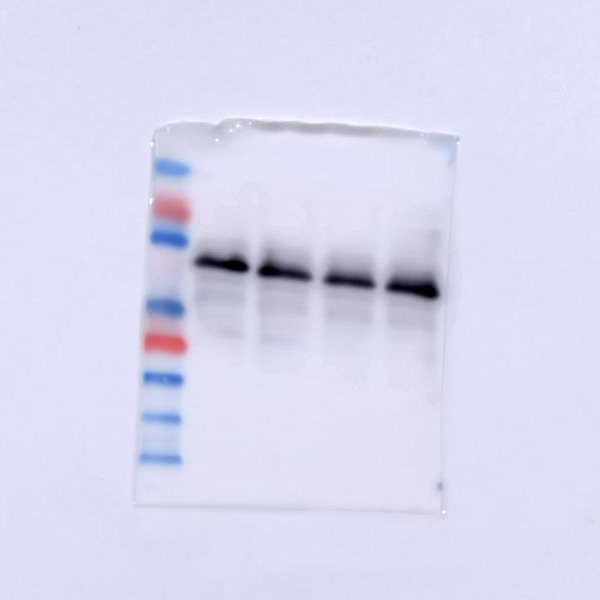


MTDH in Fig. S7e


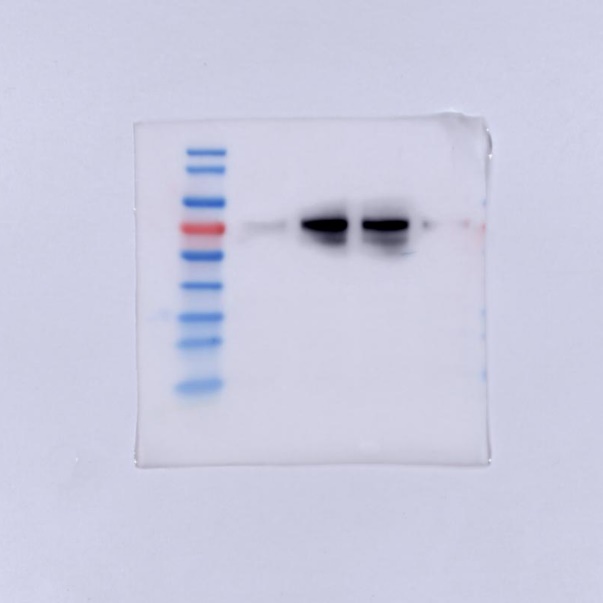


p38-MAPK in Fig. S7e


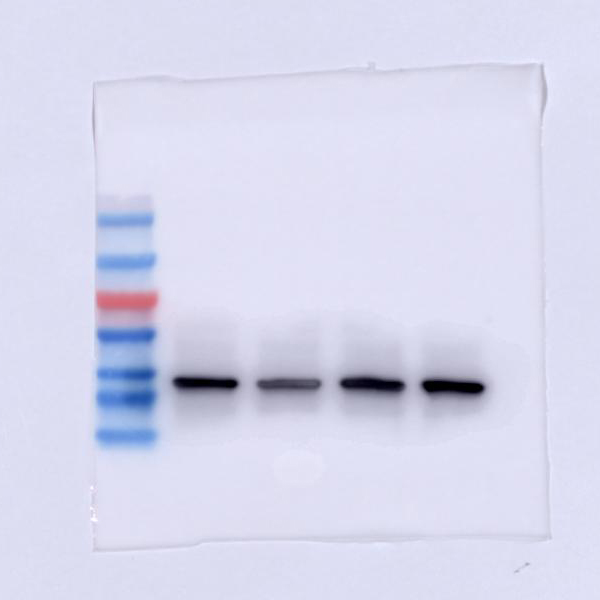


phospho-p38-MAPK in Fig. S7e


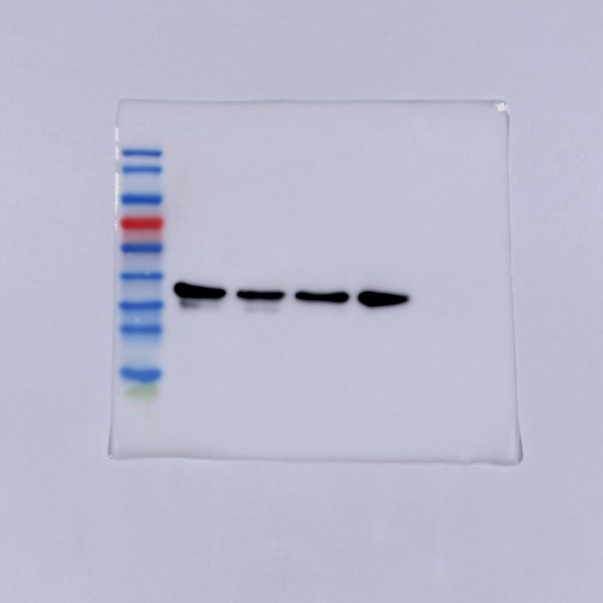


AKT in Fig. S7e


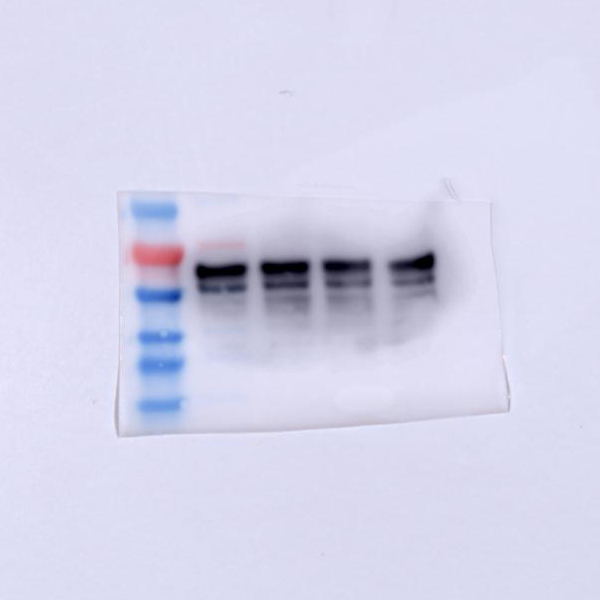


phospho-AKT in Fig. S7e


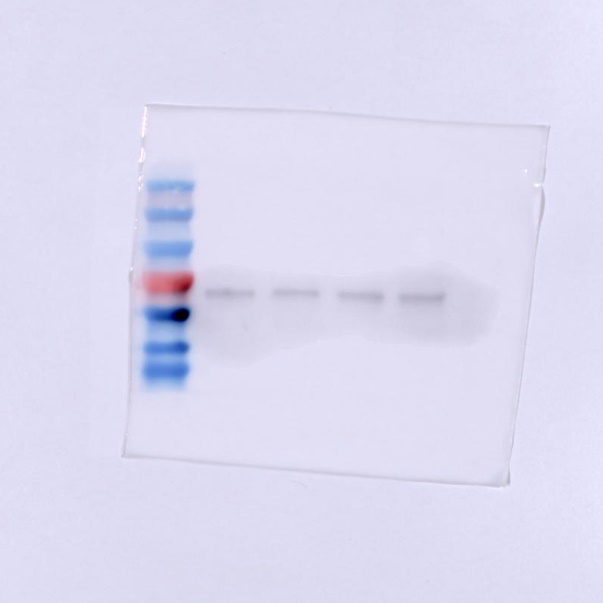


NF-κB in Fig. S7e


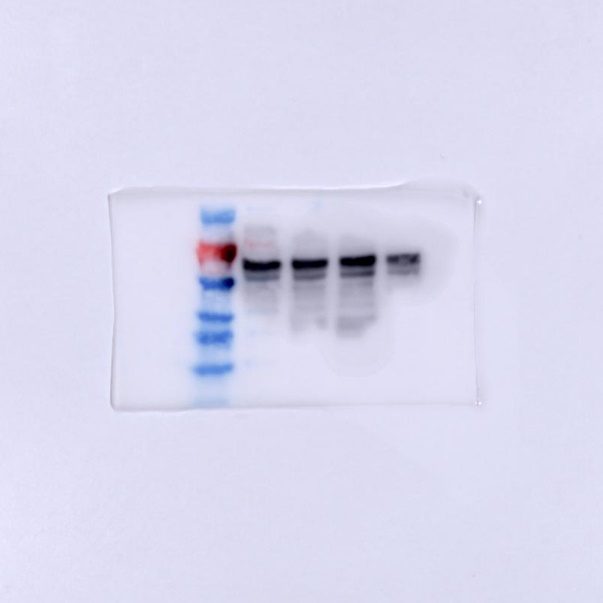


phospho- NF-κB in Fig. S7e


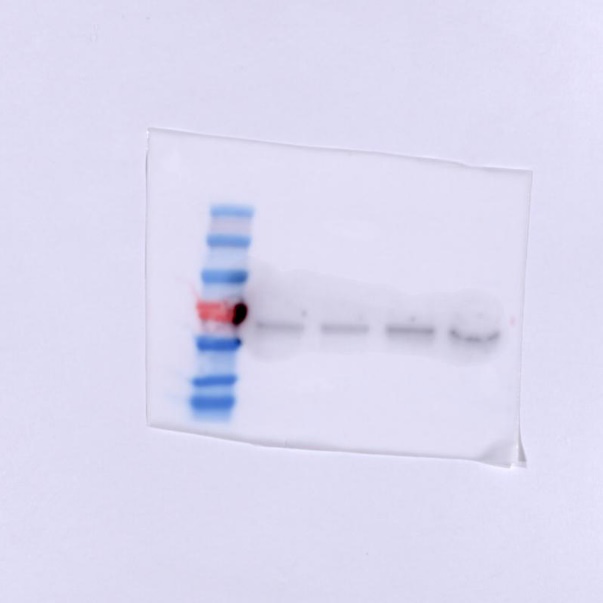


β-catenin in Fig. S7e


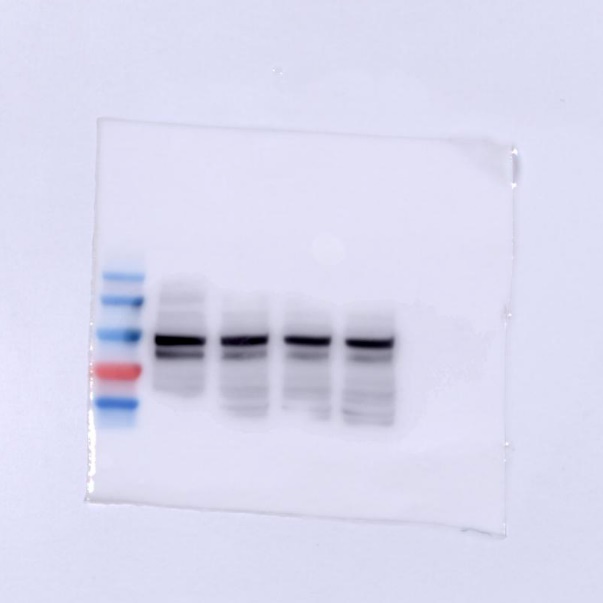


phospho-β-catenin in Fig. S7e


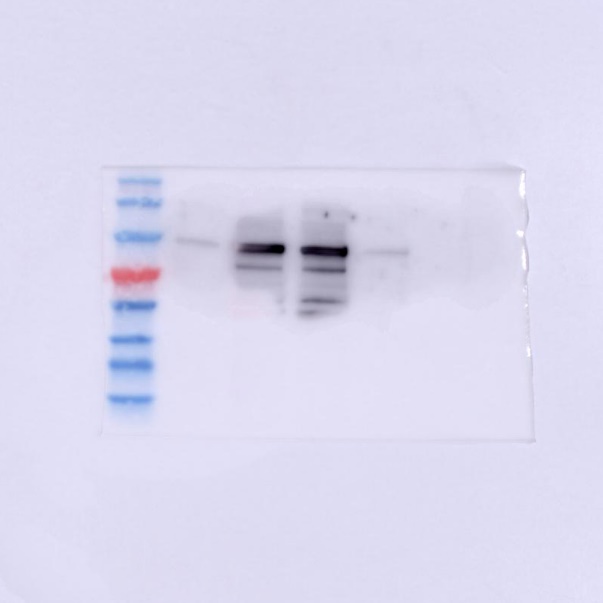


GAPDH in Fig. S7e


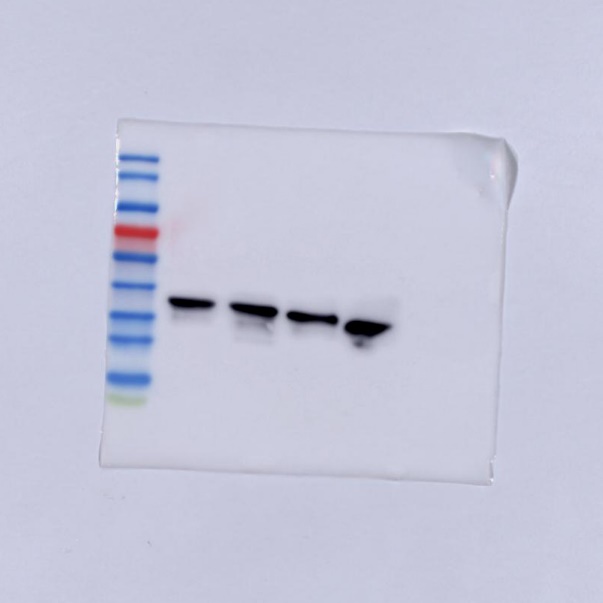


β-catenin in Fig. S7f


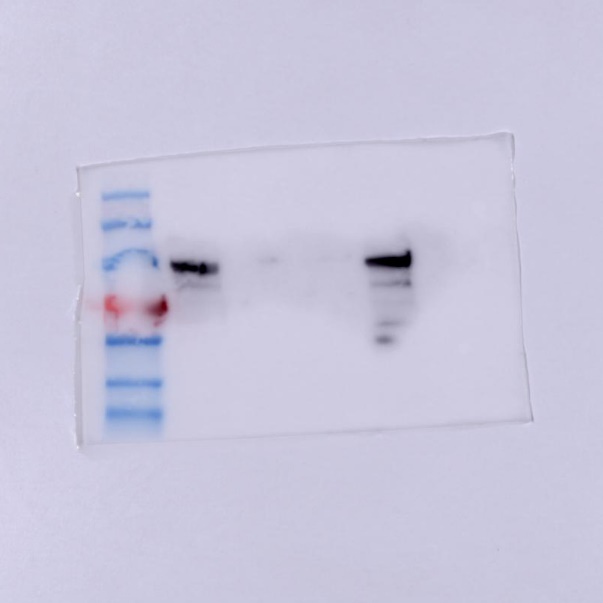


GADPH in Fig. S7f


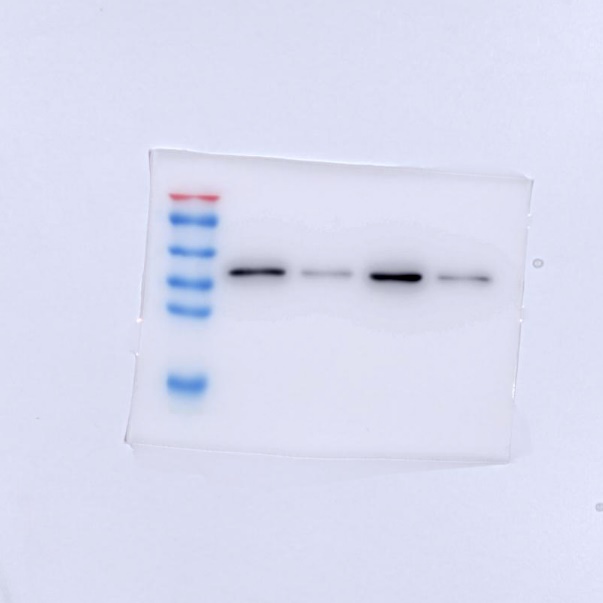


Histone H3 in Fig. S7f


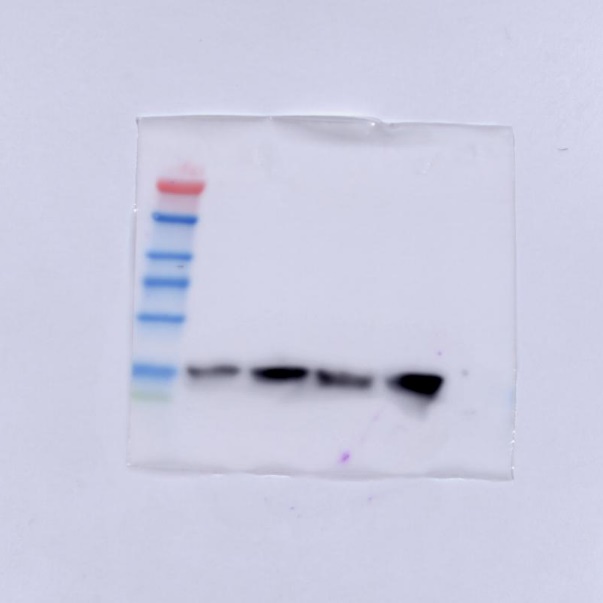


E-cadherin in TE1/CDDP-R in Fig. S8c


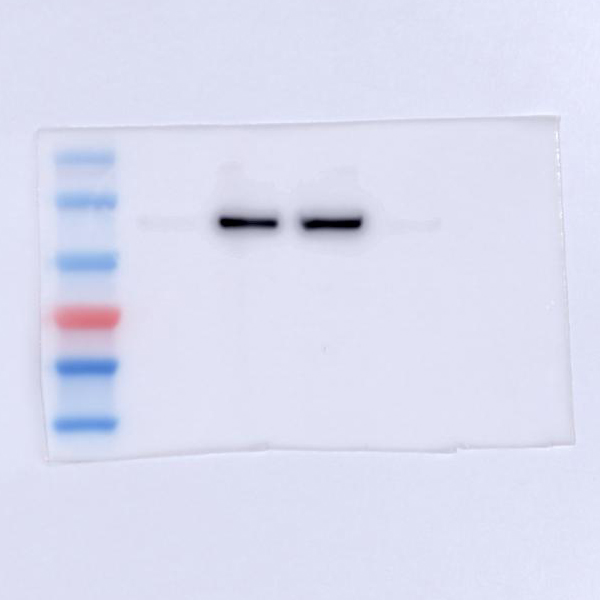


N-cadherin in TE1/CDDP-R in Fig. S8c


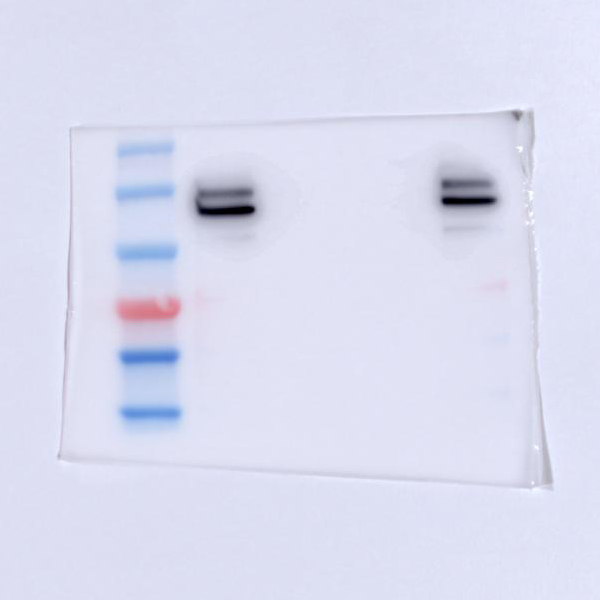


MMP9 in TE1/CDDP-R in Fig. S8c


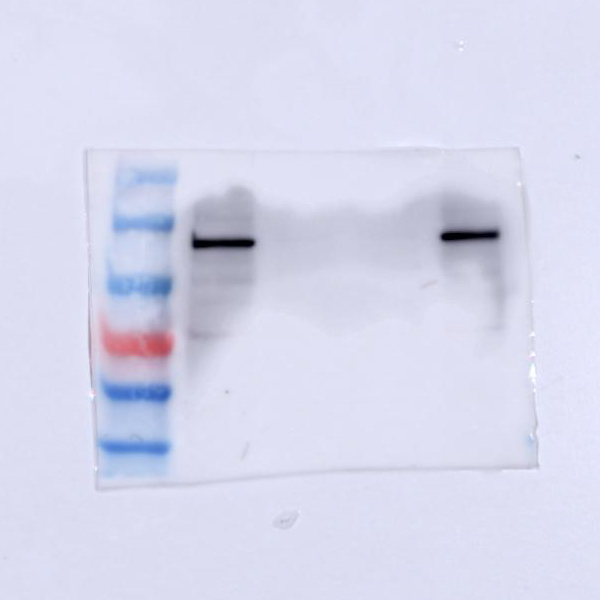


GAPDH in TE1/CDDP-R in Fig. S8c


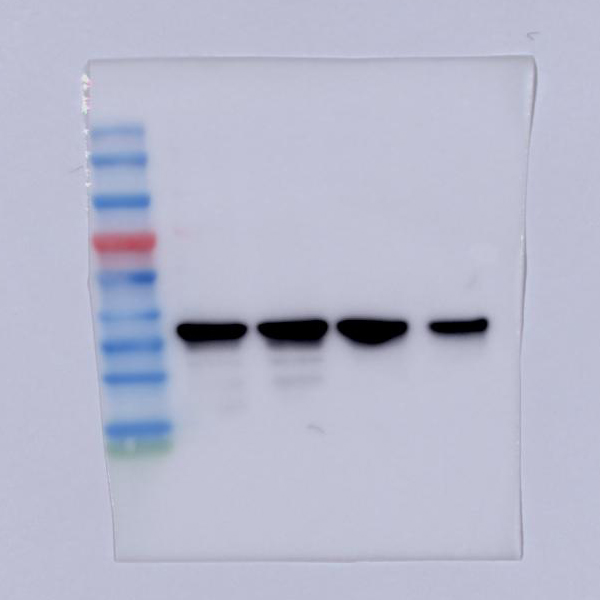


E-cadherin in TE1 in Fig. S8c


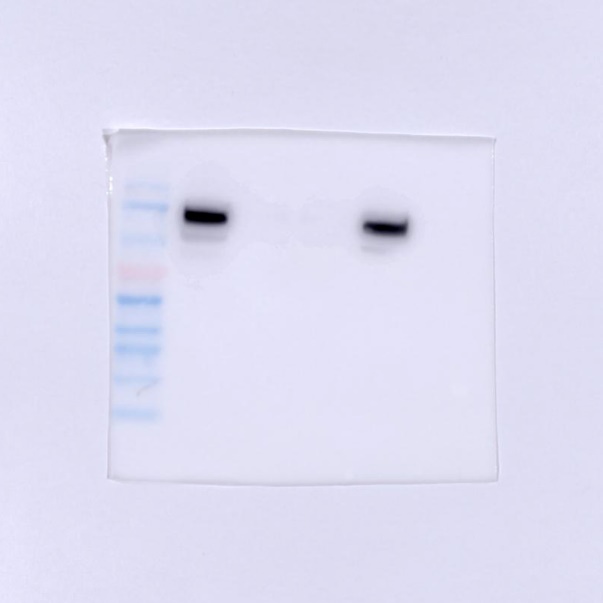


N-cadherin in TE1 in Fig. S8c


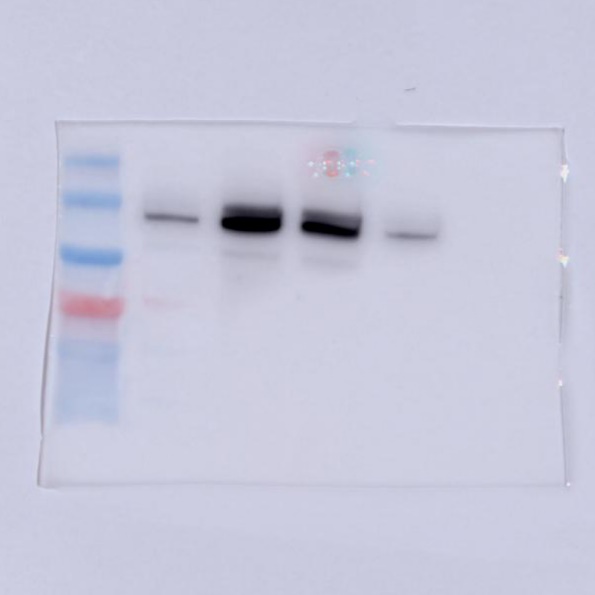


MMP9 in TE1 in Fig. S8c


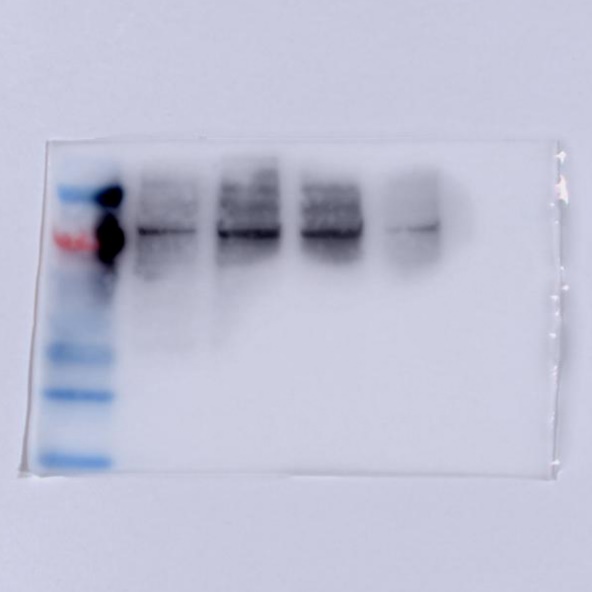


GAPDH in TE1 in Fig. S8c


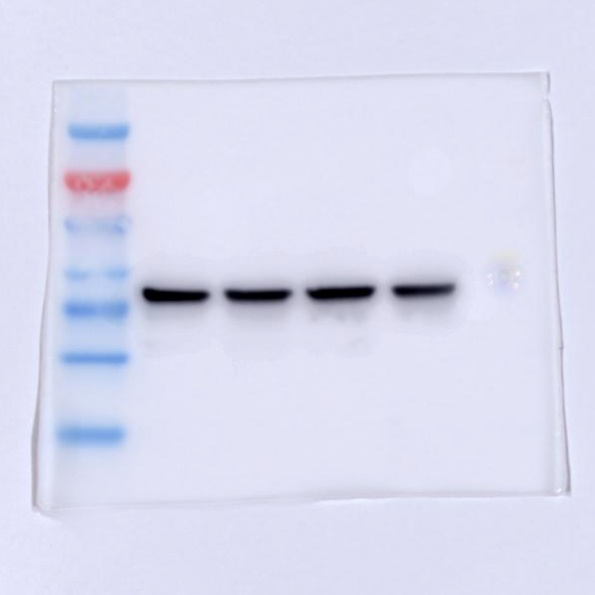

Supplement: Supplementary file 4 — Additional file 4. [file 12943_2021_1455_MOESM4_ESM.docx]
